# Minimizing batch‐to‐batch variability of a live virus vaccine by process analytical technologies

**DOI:** 10.1002/btpr.70037

**Published:** 2025-05-22

**Authors:** Katherine Forrester, Thomas R. Blanda, Marena Trauger, Rachel Thompson, Neil Templeton

**Affiliations:** ^1^ Process Research & Development Merck & Co., Inc. Rahway New Jersey USA; ^2^ Analytical Research & Development Merck & Co., Inc. Rahway New Jersey USA

**Keywords:** bioprocess, bioreactor, capacitance, dissolved oxygen, harvest, lytic virus, mammalian cell culture, microcarrier, oxygen uptake rate

## Abstract

For bioprocesses producing live virus, such as enterovirus Coxsackievirus A21, viral titer (infectivity basis) decay rates can exceed 30% within a day. Consequently, harvest timing is paramount. To optimize titer at harvest, a continuous viral product titer model was generated to elucidate kinetics. The model leveraged experimentally determined viable cell density, cell‐specific viral productivity, and viral specific decay rates. Next, three separate online process analytical technology (PAT) harvest triggers were developed to predict maximal viral titer. Finally, the PAT harvest triggers were tested alongside traditional time‐based harvests. The harvest triggers utilized common bioprocessing tools – dissolved oxygen (DO) and capacitance probes – to track DO and viable cell volume (VCV) and derived a third parameter, cell‐specific oxygen uptake rate. Harvesting with PAT triggers allowed for significantly improved batch‐to‐batch consistency. The standard deviation of harvest yield was reduced by 41% (DO), 56% (OUR) and 71% (capacitance) as compared to the industry standard time‐based harvest. Even when a process deviation in inoculated cell density occurred, causing a significant shift in viral titer kinetics, the PAT harvest triggers yielded greater than 87% of peak titer. By comparison, the time‐based harvest yielded 16%.

**Abbreviations**
ANOVA: analysis of variance
CHO: Chinese hamster ovary
Cp: capacitance
CVA21: coxsackievirus A21
DF: degrees of freedom
DO: dissolved oxygen
DPI: days post infection
ED50: effective dose 50%
EMEM: eagle's minimum essential media
FDA: food and drug administration
FTIR: fourier‐transform infrared spectroscopy
MOI: multiplicity of infection
MRC5: medical research council cell strain 5
OPC: open platform communication
OUR: oxygen uptake rate
PAT: process analytical technology
SK‐MEL‐28: human melanoma cell line
SSE: specific productivity
TCID50: tissue culture infectious dose 50%
VCD: viable cell density
VCV: viable cell volume
VII: viral imaging of infectivity
WMEM: William's E medium

## 1 INTRODUCTION

Process analytical technology (PAT) has been encouraged by the FDA since the early 2000s to increase process understanding through data‐rich measurement, ensure product quality, and facilitate manufacturing control.^1^, ^2^ When implemented early, this technology enables better process understanding over a longer time to ensure quality by design.^3^ Online bioprocess monitoring allows for improved process control, process optimization, and process understanding in real time. This reduces process deviations or staffing needs and enables automation, which can optimize yield. Implementation of in‐situ PAT in biohazardous processes may limit the requirements for offline sample handling, thereby reducing the risk to operators. Harvest viral titer filtrate is measured via cell‐based assays, a time‐intensive activity commonly requiring 1–2 weeks of time. This reduces the ability to rapidly iterate and develop a process, including harvest timing optimization. Alternatively, process monitoring offers near real‐time results. It includes various additional probes—such as capacitance, RAMAN, and FTIR—or may include probes that are already present in many bioreactors, such as a dissolved oxygen or pH probe.^4^, ^5^

For a live virus bioprocess, parameters such as cell viability, cell density at key process points, and others are critical to process success.^6^, ^7^ Similarly, viral kinetics are key: rates of viral production inside the cell, secretion to media/product build‐up in host cell, as well as viral decay rate all contribute to the measured infectious titer. Decay rate in particular is unique to live viruses‐it is commonly negated in monoclonal antibody production. Given the complexities of the system, balancing viral production and degradation rates to harvest at maximum infectious titer is key to ensure highest possible bioreactor productivity at harvest. This can drastically decrease the cost of goods, as greater yields from each batch can reduce the overall number of batches necessary.^8^

Viral titer decay rates, as they impact infectivity, are typically host‐cell/virus specific and may be impacted by a variety of process changes (culture media/buffer, temperature, pH) and should be considered when determining optimal harvest time.^9^, ^10^, ^11^ To investigate harvest timing for the desired bioprocess, three PAT parameters (dielectric spectroscopy, oxygen uptake rate, and dissolved oxygen) were utilized to build time‐predictive models to determine when peak titer occurred across both baseline and deviant batches. The methodologies used here for live virus production in mammalian cell culture have the potential to be broadly applicable to other industrial bioprocesses.

Dielectric spectroscopy, or capacitance, is commonly used in the biopharmaceutical industry as an indicator for viable cell density or viable cell volume. The basics of dielectric spectroscopy are that the in‐situ probe produces an alternating electric field that polarizes the membrane of intact cells, allowing for them to act as capacitors.^12^ The response—dielectric permittivity of the culture population in that field—is subsequently measured by the in‐situ probe. Cells with faults in membranes or broken membranes are not polarized. Changes to cell properties occur as a result of the infection (e.g., morphology, size, metabolic state) and this can subsequently impact the dielectric properties of the cell population.^13^, ^14^ For this reason, correlations are typically done to viable cell volume rather than viable cell density, accounting for shifts in cell size over the course of a process.

Despite this knowledge, inconsistencies between viable cell volume (VCV) and dielectric spectroscopy measurements are still observed when attempting to build simple linear regressions.^15^, ^16^ The main challenge often lies in the offline cell counting methodology, which is built for high‐throughput cell counting and is insensitive to changes in cell morphology, unlike microscopy‐based cell counting methods. Often, a Cole‐Cole model or partial least squares regression is used.^16^, ^17^, ^18^, ^19^ In the literature, there are various methods that have been described to correct for this difference, such as a continuous correction factor modeling the high and low viability process phases separately.^20^, ^21^, ^22^, ^23^ For the purpose of this work, a linear regression was done to minimize sum squared error with timepoint‐specific variance across the entire batch. Given that the model agreed with the VCV determined by the cell counting assay (the model did not differ in a statistically meaningful way from the experiment result), as well as the general simplicity of the approach, this was deemed an acceptable model for the use case. That said, correction for deviation should be considered if looking to implement in other systems.

The two commonly used commercialized probes for dielectric spectroscopy are from Aber Instruments^24^ and Hamilton.^25^ This technology has been heavily implemented with suspension mammalian cells,^16^, ^17^, ^18^, ^19^ especially Chinese hamster ovary (CHO) cell cultures,^17^, ^26^, ^27^ as well as use in adherent mammalian/microcarrier processes.^27^, ^28^, ^29^, ^30^, ^31^, ^32^, ^33^ One such application also investigated the harvest timing window, using peak permittivity to predict optimal harvest timing in a microcarrier/live virus platform.^27^

Oxygen Uptake Rate (OUR) measures the rate of oxygen consumption by the culture during a bioprocess. Oxygen supply is catered to oxygen demand. Oxygen supply is a function of process conditions that impact mass transfer (temperature, buffer capacity, gassing scheme, etc.). Oxygen demand is a function of the physiological and metabolic state of the cultured cells.^34^ For a process with defined controls, mass transfer coefficients can be measured experimentally. This allows for a comparison of batch‐to‐batch OUR, dependent only on culture performance. OUR can be measured on a volumetric basis (such as vessel working volume) and can also be specific to the viable cell concentration (such as viable cell density or viable cell volume).

For microbial fermentation processes, especially in cases of aerobic fermentation where oxygen transfer can be limiting, gas analyzers are used.^34^ The same is not typically true for mammalian cell culture, as there is a significantly lower OUR and consequentially less oxygen mass transfer supply required.^35^, ^36^, ^37^, ^38^ Mass spectrometers may be cost‐prohibitive, but their increased sensitivity has allowed for limited applications of OUR determination in mammalian cell culture.^39^, ^40^, ^41^ Instead, the calculation of OUR in mammalian bioprocesses is typically done via dissolved oxygen (DO) probes in situ with experimental or calculated k_L_a and assumed solubility of oxygen in culture media.^35^, ^42^ A review of methods for oxygen uptake rate determination in mammalian cell culture has been published.^38^

OUR is very commonly used as an indicator for metabolic state or culture density.^36^, ^37^, ^38^, ^43^ This includes instances where increased oxidative metabolism (requiring oxygen as an input) has also been associated with higher antibody production.^44^ Increased oxygen transfer has been shown to increase protein production in some expression systems.^45^, ^46^, ^47^ For these reasons, OUR is considered an indicator for viral production as a large cellular demand of oxygen is observed in the production of viral proteins.^42^, ^48^, ^49^ Previously, work has been published for live virus/microcarrier infection looking at the relation between cytopathic effect (typically an indicator of infection) and OUR.^32^, ^50^

Dissolved oxygen (DO) probes are routinely used in industrial fermentation and cell culture processes to allow for measurement and process control. In the production of a live virus in cell culture, cell health, viable cell density, and viability often decline in the late stages of infection.^27^ As a result, oxygen consumption (see above) decreases and causes a subsequent rise in the DO concentration. This applies to many cell culture processes where only one‐sided DO control is used to maintain a minimum DO concentration.

This paper explores a cross‐comparison between multiple industry relevant methods for process monitoring and decisions. Specifically, this work explores three approaches for harvest triggers, including: capacitance, dissolved oxygen, and oxygen uptake rate. Methods are compared based on the specific application, use case, or available resources.

## 2 MATERIALS AND METHODS

### 2.1 Bioreactor operation

Live Coxsackievirus A21 (CVA21) was produced using glass Eppendorf 3 L BioFlo vessels operated through BioFlo 320 Eppendorf bioreactor control towers. MRC5 cells were expanded in Corning Cells Stacks and then cultured on Cytodex‐1 microcarriers in William's E Medium (WMEM—GIBCO) supplemented with 10% Bovine Calf Serum, 0.1% P188, 4 mM L‐glutamine, and 20 mM glucose. After approximately 3 days post inoculation, once the cells had reached 80% or greater confluency (as determined visually through a microscope), an 80% media exchange occurred into serum‐free medium. Before media exchange, the bioreactor was controlled at a constant temperature of 37°C, a DO of >40%, and a pH of 7.2 ± 0.05. Temperature was controlled with a water jacket while pH was controlled using 1 M sodium carbonate and sparged carbon dioxide gas. After the media exchange, the temperature was decreased to 34°C while the pH setpoint was maintained. Throughout the entire process, 2 mL/min of air was sparged into the culture while 280 mL/min air was supplied to the headspace of the vessel – the exchange volume of the headspace was approximately every 7 min. Following the temperature shift, the bioreactor was inoculated using a CVA21 virus stock at a multiplicity of infection of 0.05 infectious virions per viable cell. The infection progressed for approximately four days until 95% or greater cellular lysis was observed microscopically. Samples were taken 1–2 times per day during the infection phase. Quantification of cellular density, viability, and cellular volume were accomplished through the Nucleocounter200 (Chemometec) A100 + B with Via‐Cassette assay and infectivity was assessed via a high‐throughput infectivity assay.^51^

### 2.2 Viral decay rate

**FIGURE 1:**
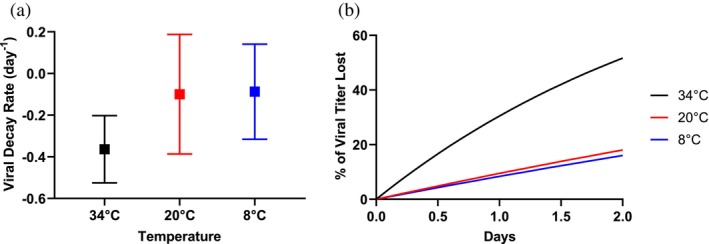
The decay of Coxsackievirus A21 over time at several different temperature conditions. Production temperature (34°C, *N* = 8 batches) in black, approximate room temperature (20°C, *N* = 8 batches) in red, and a refrigerated temperature (8°C, *N* = 7 batches) in blue. (a) Average viral decay rates are plotted with one standard deviation. (b) Mean product loss over time from calculated decay rates, leveraging Equation (1). At Day 0, 100% of viral titer remains, meaning 0% of viral titer has been lost. Welch's ANOVA test determined a significant difference amongst the means with a *p* > 0.05. A post‐hoc Dunnett's T3 multiple comparisons test indicates a significant difference comparing the 34°C condition to both 20°C or 8°C (*p* < 0.001). There was not a significant difference between the decay rate at 20 and 8°C conditions (*p* = 0.99).

To determine the viral decay rate, after complete cellular lysis—as determined via brightfield microscope visual and Nucleocounter 200 assay viability—temperatures were shifted to their cognate setpoints (34, 20, and 8°C) and samples were taken over a two‐day period for each of the different temperature conditions. During this two‐day period, all other production conditions were unchanged. Samples were analyzed through a high‐throughput infectivity assay (VII) and the viral decay rate was calculated using Equation (1). Given that complete cellular lysis had occurred, it was assumed that the Q_P_X term was negligible in calculating k_d_. Once the viral decay rate was determined, Equation (1) was used to determine virus decay over time, reported in Figure 1.

(1) $$\frac{\mathrm{dP}}{\mathrm{dt}}=-k_{d}P+Q_{p}X$$

The viral decay rate (k_d_) determined from changes in viral infectivity (P) values as determined by the VII assay over time (t). Changes in viral infectivity over time is also influenced by the specific productivity (Q_p_) of the virus and the viable cell volume (X).

### 2.3 Dissolved oxygen (DO) data collection and conversion

The optical DO probes used for experimentation reported values as a percent saturation (%) (with ambient air considered 100%) in the culture media. The dissolved oxygen concentration was maintained using sparged oxygen through a one‐sided controller. For baseline condition batches, the minimum set point was not reached (*N* = 13); therefore, the inlet of air (through overlay and sparge) to the bioreactor remained constant and any decline in DO levels in the bioreactor is due to cellular demand for oxygen. To determine the oxygen consumption rate, the percent DO unit was converted to μM oxygen in the culture. This was accomplished using the osmolarity of the culture (a selected constant from measured offline values), the temperature of the culture (a controlled setpoint), and the percent variable DO (percent air) value. Pressure was assumed to be constant and variation in pressure was considered to be negligible in the system. After referencing a conversion table for mg/L of dissolved oxygen at varying osmolarities/temperatures, the oxygen concentration in the culture at each time point was determined.^52^

### 2.4 Viable cell volume (VCV) conversion

Viable cell density (VCD) and cell diameter were determined using the Nucleocounter‐200 A100 and B with Via cassettes assay. Capacitance (Cp) was collected using the in‐situ Aber Futura system (Aber Part numbers: 2330‐00, 2801‐00, and 6532‐52/PG) at a 580 kHz measuring frequency with 15,650 kHz background correction and at a 30‐s trailing interval in the Futura Tool software to generate a corrected capacitance measurement (Cp_580,15,650_). Viable cell volume (VCV) was calculated using Equation (2), resulting in units of μm^3^* cells/mL. Linear regression was performed to relate a capacitance measurement to VCV. Sum squared error (SSE) in VCV was minimized to generate a best fit regressing 76 experimental VCV and Cp_580,15,650_ measurements taken from 18 independent full‐length batches. When minimizing the SSE of the model and experimental data, model fit was weighted by the variability of each VCV measurement, represented by a standard error term. Conversely, variation in Cp_580,15,650_ was considered negligible (± 3% of the reading^53^) as it tended to vary considerably less (when represented as coefficient of variance) than VCV.

(2) $$X=\frac{1}{6}\pi D^{3}$$

The viable cell volume (X) is determined from the viable cell density (D) with the assumption that the cells are spherical.

Correlating Cp to VCD was considered as an alternative to VCV. Ultimately, since MRC5 cell diameter was determined to grow following infection, VCV proved to correlate better than VCD with Cp, since biocapacitance is affected by both cell size and cell density.^23^ Subsequently, observed oxygen consumption rates likewise consider cell size and density.

### 2.5 Oxygen uptake rate (OUR) equation derivation

The derivation of the OUR equation started from a mass balance for oxygen in the bioreactor system. The mass balance was based on the generic equation:

(3) $$\text{ACCUMULATION}=\begin{array}{l} \text{INPUT}+\text{GENERATION}-\text{OUTPUT}\\ -\;\text{CONSUMPTION} \end{array}$$

Generic mass balance equation.

For this system, the accumulation of oxygen in the bioreactor can be tracked in the DO percentage measured by the optical DO probe (see above section for DO calculation). The inputs of oxygen to the bioreactor come from overlay and sparge gassing. To properly determine oxygen delivery, the k_L_a values for the given media type, agitation speed, and superficial gas velocity were determined experimentally. Neither oxygen input nor output terms were solved for independently. By including VCV (see above), the oxygen uptake rate can be calculated on a specific cell volume basis, giving further insight into the metabolic state of the culture. Please see Supplemental Equation (1), solved for cell‐specific OUR (q).

(4) $$\frac{\mathrm{dC}}{\mathrm{dt}}=-\mathrm{qX}+k_{L}a_{o,\mathrm{air}}\left(C_{o,\mathrm{air}}-C_{v}\right)+k_{L}a_{s,\mathrm{air}}\left(C_{s,\mathrm{air}}-C_{v}\right)+k_{L}a_{s,O_{2}}\left(C_{s,O_{2}}-C_{v}\right)$$

Mass balance of oxygen for the bioreactor system. The concentration of oxygen is denoted by uppercase C with the subscripts referring to either the vessel (v) obtained via optical DO readings, or overlay (o)/sparge (s) gassing inputs of pure oxygen (O_2_) or air (air). The oxygen uptake rate on a cellular volume basis is denoted as q, which is multiplied by the viable cell volume (X) as determined from capacitance probe readings.

### 2.6 Growth rate calculation

Growth rate was calculated from continuous online capacitance data. To negate any potential noise from the probe, the growth rate was determined by calculating the slope of a semi‐log plot of VCV over the course of approximately one hour. Due to the slow growth rates observed in mammalian cell culture, the viable cell volume did not change significantly over any hour‐long period.

(5) $$\frac{\mathrm{dX}}{\mathrm{dt}}=\mathrm{\mu X}$$

Growth rate (μ) was calculated using the above equation, factoring in the viable cell volume (X) as determined through online capacitance probe readings over time (t). Practically, this number was determined through the slope of a semi‐log plot over the course of approximately an hour.

### 2.7 High throughput infectivity assay

Viral titer measurements for Coxsackievirus A21 were determined by Viral Imaging of Infectivity (VII), an infectivity assay that uses protein expression to measure viral infection.^51^ SK‐MEL‐28 cells sourced from Merck & Co., Inc., (Rahway, NJ, USA) cell banks were seeded in EMEM (2% FBS, 1% Pen Strep) at 16,000 cells per well in 384‐well poly‐D‐lysine tissue culture microplates. Cells were incubated at 37°C, 5% CO2, >90% relative humidity and allowed to attach overnight to form a confluent cell layer. SK‐MEL‐28 cells were infected with Coxsackievirus A21 for 8 h and then plates were fixed. Cells were then permeabilized, stained with 1 μg/mL Hoechst 33342 (cat. H3570; Invitrogen™) for nuclear DNA, and immunostained with a 2.5 μg/mL Purified Rabbit Anti‐CVA21 pAb and labeled with a 1:200 diluted Alexa Fluor® 488 AffiniPure Donkey Anti‐Rabbit IgG (cat. 711‐545‐152; Jackson ImmunoResearch Inc., PA, USA) detection antibody. Cells were imaged for stained nuclei and the fluorescently tagged viral protein on a BioTek Cytation™ 3 or Cytation™ 5 reader (Agilent Technologies, CA, USA). Images were analyzed using the reader's software to calculate the % infected ratio from the infected (tagged viral protein) to total (Hoechst) cells. A four‐parameter logistical fit is calculated for the dilution series. Relative viral titer is calculated by the ratio of sample ED50, dilution at which 50% of cells are infected, to reference ED50 and reported as a percent response. Viral titer (in TCID50/mL) for samples was calculated using the VII reference standard (titered in TCID50).

### 2.8 Specific productivity and theoretical titer calculation

Specific productivity (Q_p_) was calculated using Equation (1). Specific productivity gives insight into the amount of virus an individual cell (or in this case a certain cellular volume) can produce independent of the number of cells in the culture. This calculation was performed at each sampling time point during the infection portion of the process.

Derived from the specific productivity calculation using Equation (1), an iterative formula was derived to calculate the infectious titer continuously (see Supplemental Equation 2). To provide estimations for the specific productivity between discrete titer sampling time points, various interpolation methods were tested: step functions, linear functions, and combinations of the two. Ultimately, a step function was used to estimate specific productivity between time points to feed into the iterative formula and calculate estimations of viral titer in the culture. The time between infection and the first viral titer sample also used a step function to estimate Q_p_, which does not account for the early lag in virus production that is observed in most live‐virus processes.

## 3 STATISTICAL ANALYSES

### 3.1 Product decay rate (Figure 1)

A Welch's one‐way ANOVA (*p* < 0.05, DFn = 2, DFd = 119.5, W = 40.34) was completed in GraphPad Prism on the experimentally determined decay constants and a significant difference between means was identified (*p* < 0.0001). A post‐hoc Dunnett's T3 multiple comparison test (*p* < 0.0001) was used to compare product decay rates between groups. No significant difference was found between the mean of all decay rates at 20°C (*N* = 8 batches) versus 8°C (*N* = 7 batches) with an adjusted *p* value of 0.9908 (DF = 122.8), but significant differences were observed between 34°C (*N* = 8 batches) versus 20 and 34°C versus 8°C with adjusted *p* values <0.0001 (DF = 103.1, 109.5, respectively).

### 3.2 Correlating capacitance to VCV

Linear regression was performed to relate a capacitance measurement to Nucleocounter determined VCV. Sum squared error (SSE) in VCV was minimized to generate a best fit regressing 76 experimental VCV values (*N* = 3 per timepoint) and Cp_580,15,650_ measurement (*N* = 1 per timepoint) taken from *N* = 18 independent full‐length batches. When minimizing the (sum‐squared error) SSE of the model and experimental data, model fit was weighted by the variability of each VCV measurement, represented by a standard error term. Conversely, variation in Cp_580,15,650_ was considered negligible as it tended to vary considerably less, in relative terms, than VCV. A Χ^2^ approach was used to compare the model for VCV to that of the Nucleocounter assay (DF = 74, Χ^2^ = 91.96, *p* = 0.08).

### 3.3 Χ^2^ comparison of theoretical titer to assay titer

**FIGURE 2:**
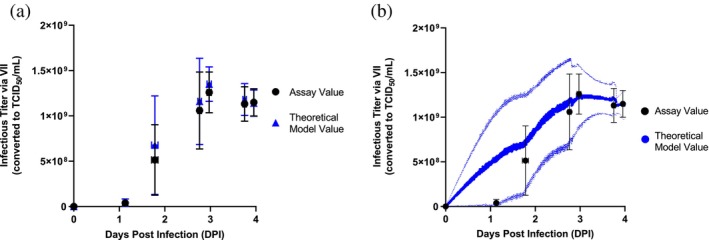
Theoretical model comparison to assay‐measured titer data. *N* = 25 batches are included in the overall dataset however, the timepoints have different numbers of samples. The timepoints from left to right include *N* = 25, *N* = 4, *N* = 25, *N* = 24, *N* = 17, *N* = 20 and *N* = 5 datapoints. The horizontal error bars represent the standard deviation of the time at which the sample was taken for the assay. The vertical error bars represent the standard deviation of the infectious titer value—assay or theoretical—respectively. (a) Includes only the timepoint of the sample from the continuous model. At data points with fewer than the total number of batches, only corresponding theoretical model values are represented. (b) Includes the entire continuous model of viral titer with the light blue lines representing the standard deviation over time. A Χ2 test was performed on the two discrete data sets in Figure 2a to determine if the theoretical predicted value is independent of the assay value. The *p* value of 0.73 supports that the theoretical predicted values are not statistically different from the assay values.

A Χ^2^ test was performed to evaluate if the theoretical continuous titer trend was statistically different from the assay‐determined values. For this analysis, *N* = 25 batches with *N* = 99 individual data points were considered (Figure 2). For an accurate comparison, the continuous titer value at the timing of the assay values were pulled for comparison. From an independent data set, the assay coefficient of variance was determined as 20%. The coefficient of variance for the assay was used to determine the standard error of the assay values. Taking the degrees of freedom (DF = 97) and the summed square error (Χ^2^ = 87.93), the determined *p* value was 0.74, which was greater than the significance level of 0.05.

### 3.4 Brown‐forsythe test for the equality of variance

To compare the variance in predictive capacity of the different harvest triggers, a Brown‐Forsythe Test was performed (Figure 4). The percent of maximum overall titer for all *N* = 13 baseline batches was analyzed for each harvest trigger (time harvest, DO based mathematical trigger, VCV based mathematical trigger and OUR based mathematical trigger). Due to the non‐Gaussian distribution of the data sets—as determined by a Shapiro–Wilk's test on the residuals—the Brown‐Forsythe test was utilized for its robustness with non‐normal data (DFn = 3, DFd = 48, F = 3.99, *p* = 0.01, significance level = 0.05).

## 4 RESULTS

When testing the stability of the virus (see Figure 1) at various temperatures (34, 20, 8°C), the decay rate of infectious virus was found to be substantial at the production temperature (34°C). At this production temperature, approximately 30% of the infectious particles can be lost within one day; however, the decay rate can be attenuated with reduced temperature. At room temperature (20°C), only approximately 9% of the viral product is lost within one day, while at refrigerated temperature (8°C), only approximately 6% is lost over a one‐day period.

Three continuous online parameters were investigated as potential correlative measures for the timing of peak viral titer: dissolved oxygen (DO), viable cell volume (VCV), and oxygen uptake rate (OUR). Due to difficulties in building statistically significant models that correlate by‐minute online parameters to discrete data points, a theoretical continuous infectious titer model was derived and implemented using Supplemental Equation 2. Figure 2 represents the model's ability to predict viral titer at discrete time points to determine if the model could be used for intermediate time points that were not quantitated by an offline viral titer assay. For all time points, the average theoretical continuous titer value fell within 1 standard deviation for the average assay determined values. In Figure 2a, from the *N* = 4 batches with data points at 1DPI, the data indicates there is little to no viral production within the first day. However, for the *N* = 21 batches without an early data point, the specific productivity calculated from the viral titer at 2DPI yields a falsely high viral titer in the early time frame. This results in a high error for the model in the early time frame.

**FIGURE 3:**
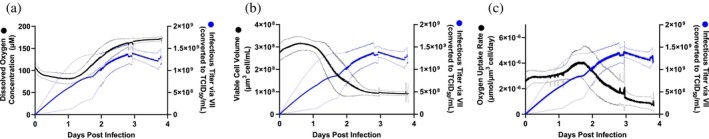
Online PAT parameters and viral titer measurements. (a–c) The blue data represents the theoretical continuous viral titer over the production phase averaged across *N* = 13 baseline batches. The dotted lines represent one standard deviation of the measurement at each time point. The black data represents average dissolved oxygen, viable cell volume, and oxygen uptake rate parameters across *N* = 13 batches. Dotted black lines represent one standard deviation of averaged parameters.

In Figure 3, the three real‐time parameters are plotted against infectious titer for all baseline batches (*N* = 13). The effects of viral infection on a host cell's metabolic state can be leveraged through various mathematical relationships to predict the optimal time to harvest.

From the biological correlation between the online parameters and continuous viral titer, mathematical criteria were developed to indicate optimal harvest timing. These PAT harvest triggers were a function of the following: time, parameter value, and parameter derivative in respect to time. To determine optimal criteria for the harvest trigger for each parameter, a computational approach was developed to address three different phases of production (Supplemental Table 1) and applied to existing batch data. For DO: (1) when the DO levels begin to rise, (2) when the DO is steadily increasing, and (3) when the DO levels begin to asymptote. For VCV and OUR: (1) where the trend begins to drop, (2) when the trend is steadily declining, and (3) when the trend begins to asymptote. To optimize harvest timing, the trigger provides an alert prior to peak yield, offering an early warning. This increases manufacturing ease. All triggers were compared by their ability to predict peak viral titer timing consistently (precision of prediction) and to predict peak titer timing (accuracy of prediction). Table 1 shows the optimal criteria determined for each harvest trigger. A comparison of the different criteria considered can be seen in Supplemental Table 2.

**TABLE 1:** Mathematical criteria to trigger harvest. All criteria have been met to trigger harvest.

	Mathematical trigger criteria	Units	Description
DO harvest trigger	DPI>1 $\frac{d\left(\mathrm{DO}\right)}{\mathrm{dt}}>25$ $\mathrm{DO}>{\mathrm{DO}}_{\min}+0.45\left({\mathrm{DO}}_{\max}-{\mathrm{DO}}_{\min}\right)$ Offset trigger → harvest: 0.96 days	day $\frac{\mathrm{\mu M}}{\mathrm{day}}$ μM	The timing in the batch must be greater than 1 day post infection The rise in DO/time is greater than 25 $\frac{\mathrm{\mu M}}{\mathrm{day}}$ The dissolved oxygen concentration must have risen 45% with respect to the maximum, from the DO minimum Once the trigger has occurred, harvest in 0.96 days.
VCV harvest trigger	1 DPI>0.5 2 $\frac{d\left(\mathrm{VCV}\right)}{\mathrm{dt}}<0$ 2 $|\frac{d\left(\mathrm{VCV}\right)}{\mathrm{dt}}|>1.5x10^{8}$ 3 $\mathrm{VCV}<{\mathrm{VCV}}_{\max}-0.1\left({\mathrm{VCV}}_{\max}-{\mathrm{VCV}}_{\min}\right)$ Offset trigger → harvest: 1.33 days	day $\frac{{\mathrm{\mu m}}^{3}\;\text{cell}}{\mathrm{mL}\times \mathrm{day}}$ $\frac{{\mathrm{\mu m}}^{3}\;\text{cell}}{\mathrm{mL}}$	1 The timing in the batch must be greater than 0.5 days post infection 2 VCV must be decreasing with time 2 The VCV/time is greater in magnitude than 1.5 $\times 10^{8}\frac{{\mathrm{\mu m}}^{3}\;\text{cell}}{\mathrm{mL}\times \mathrm{day}}$ 3 The VCV must have dropped 10% from the VCV maximum threshold point Once the trigger has occurred, harvest in 1.33 days.
OUR harvest trigger	1 DPI>1 2 $\frac{d\left(\mathrm{OUR}\right)}{\mathrm{dt}}<0$ 2 $|\frac{d\left(\mathrm{OUR}\right)}{\mathrm{dt}}|>3e^{-6}$ 3 $\mathrm{OUR}<{\mathrm{OUR}}_{\max}-0.55\left({\mathrm{OUR}}_{\max}-{\mathrm{OUR}}_{\min}\right)$ Offset trigger → harvest: 0.49 days	day $\frac{\text{μmol}\;O_{2}}{{\mathrm{\mu m}}^{3}\;\text{cell}\times \mathrm{day}}$ $\frac{\text{μmol}\;O_{2}}{{\mathrm{\mu m}}^{3}\;\text{cell}}$	1 The timing in the batch must be greater than 1 day post infection 2 OUR must be decreasing with time 2 The OUR/time is greater in magnitude than $3e^{-6}\;\frac{\text{μmol}}{{\mathrm{\mu m}}^{3}\;\text{cell}\times \mathrm{day}}$ 3 The OUR must have dropped 55% from the OUR maximum value, with respect to the minimum. Once the trigger has occurred, harvest in 0.49 days.

**FIGURE 4:**
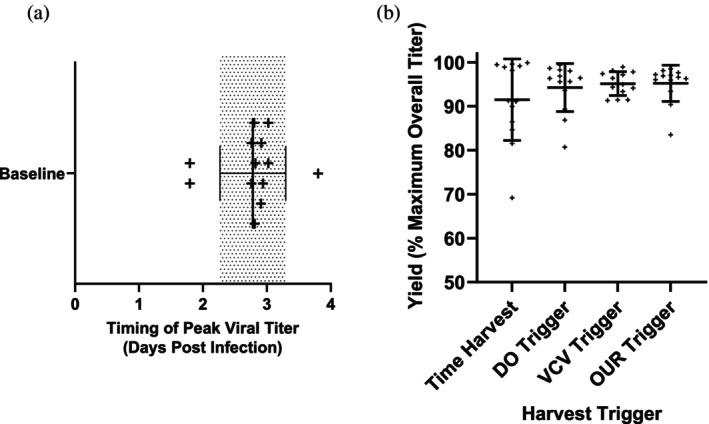
Harvest trigger evaluation for baseline condition batches. (a) The timing of peak viral titer for the *N* = 13 baseline batches is plotted – with one plus sign representing each batch. The mean is represented by the middle line with the standard deviation represented by the horizontal error bars and grayed region. (b) The three PAT harvest triggers are compared to the standard time based harvest–at the “optimal” time (average timing of peak viral titer). Each dot represents one batch with the horizontal lines representing the average and the vertical error bars representing the standard deviation. The four harvest triggers have significantly different standard deviations as assessed through a Brown‐Forsythe test of variance (*p* = 0.0128).

The three harvest triggers from each online PAT parameter were selected based on their ability to secure the highest yield (percentage of maximum titer) and batch‐to‐batch theoretical consistency. Theoretical consistency was determined by having the lowest standard deviation of time offset from maximum titer in the baseline batches (Supplemental Table 2). Figure 4a displays the variation in peak viral titer timing for *N* = 13 baseline batches—average timing was 2.78 days with a standard deviation of ±0.49 days. To initially evaluate the harvest triggers, the predicted yield based on each respective harvest trigger was compared to the yield based on the average timing of peak titer observed across all 13 baseline batches. The performance of the online harvest triggers relative to a time‐based harvest is shown in Figure 4b. Yield consistency, as reflected by the standard deviation of the yield plotted in Figure 4b, improved considerably with the three PAT harvest triggers. Time harvest had a standard deviation of 8.9% of maximum theoretical yield, while the PAT harvest triggers have standard deviations of 5.2% (DO), 2.6% (VCV) and 4.0% (OUR).

**FIGURE 5:**
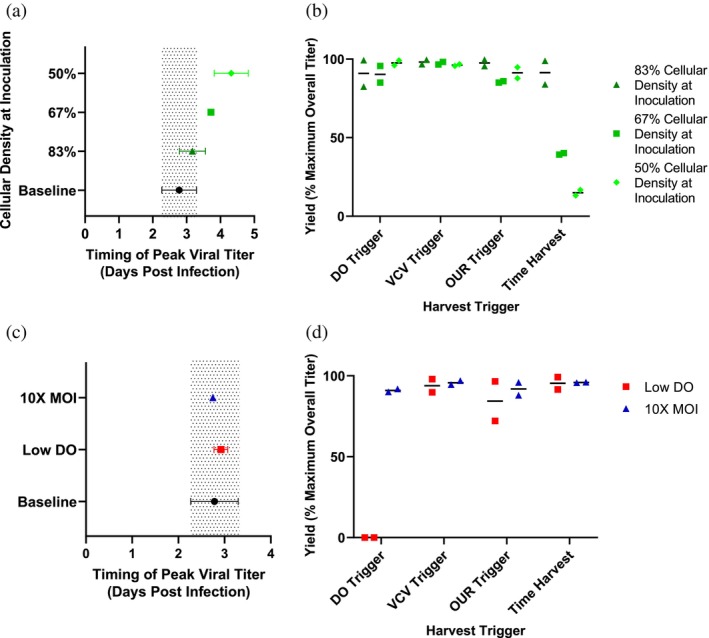
Case studies of deviant batches. (a) The average timing of peak viral titer is plotted with the error bars representing the standard deviation for each data set. The baseline group of *N* = 13 batches from Figure 4a (labeled “Baseline”) are plotted in black on the bottom and the gray region represents the range of the standard deviation showing where the timing of peak viral titer would occur for those batches. The other conditions represent *N* = 2 batches from an experiment investigating how varying cellular density at inoculation impacted cellular growth and subsequent viral progression. (b) Predicted percent yield of maximum titer for each condition is plotted for each harvest trigger in comparison with a time‐based harvest. Horizontal black lines represent the average of each condition. (c) The average timing of peak viral titer is plotted with the error bars representing the standard deviation for each data set. The deviant conditions represent two separate experiments investigating low DO (gassing N2 to force a low oxygen environment) for *N* = 2 bioreactors and tenfold increased multiplicity of infection (MOI) for *N* = 2 bioreactors. The same baseline bioreactors as Figure 5a are also plotted. (d) The predicted percent yield of maximum titer is plotted for both deviant conditions to compare the harvest triggers to a time‐based harvest. Horizontal black lines represent the average of each condition.

The second evaluation of the harvest triggers utilized three case studies of deviant batches: one in which cellular inoculation density on microcarriers was varied stepwise down to 50% of the baseline process specification (Figure 5a,b), a tenfold increase in multiplicity of infection (MOI) at the time of infection, and an alternative gassing strategy that forced dissolved oxygen to be maintained at a low oxygen set point (20% of saturated air dissolved in solution) throughout the experiment with two‐sided DO control (Figure 5c,d). As previously mentioned, baseline conditions (and all other conditions besides the low DO condition) employ one‐sided control to maintain DO levels above 40% at all times. All case study conditions were run in duplicate. The mathematical criteria determined using control batches were applied to these three case studies to assess harvest trigger predictive capacity under deviant conditions.

## 5 DISCUSSION

Maximizing yield for a viral vaccine is critical to meeting global market demands and keeping cost of goods low. This is primarily accomplished through cell line evaluation, media development, and process parameter exploration (pH, DO, agitation, etc). Development and optimization must occur while being mindful of various risks within the manufacturing setting to create an optimized system that is also suitable for a GMP environment. Understanding potential differences between varying scales is crucial in live virus production where the product is subject to rapid decay rates. For live virus production of CVA21, around 30% of the virus can decay within a day at production temperature (Figure 1). A data‐driven and scalable approach using online data can enable better yields to be obtained from each batch and prevent valuable product loss. Additionally, online data can increase process understanding to the benefit of process development and future manufacturing. Two of the three PAT harvest triggers proposed by this work are scale‐independent, a technical advantage that offers flexibility for manufacture. Cell‐specific OUR‐based harvest triggers, while scale independent, require scale‐dependent k_L_a to be determined experimentally.

Canonically, offline assays that measure infectious viral titer via plaque or TCID50 are used to build an understanding of viral kinetics; however, these techniques are time‐intensive. Even with the development of high‐throughput infectivity assays, such as a fluorescence‐based proxy assay, the time necessary for assay readout does not allow for real‐time process insights, nor does it permit high‐resolution sampling without significant personnel time cost. An alternative to post‐batch viral assays is building a regression model to trend viral production in real time. Unfortunately, univariate models are often insufficient to build reliable models for virus production, making modeling and trending virus production in real time an elusive task. Sophisticated analytical tools, multivariate empirical models or theoretical models, and complicated data communication protocols for third‐party sensor integration (e.g., OPC or Modbus) are required. We approached this problem by utilizing common bioprocessing tools —DO and capacitance probes—and derived a third parameter (OUR) utilizing the latter two, that all have theoretical correlative relationships with viral production^42^, ^48^, ^49^ to understand viral replication dynamics. Furthermore, this method for calculating OUR increases accessibility to the measurement without the need for additional equipment (such as an off‐gas analyzer or a mass spectrometer). With a more accessible method for measuring OUR, shifts in metabolic states can be more readily assessed in real time and different process control techniques can be employed, such as OUR‐based feed strategies.

These online parameters were shown to have a relative quantitative relationship with increasing viral titer in our bioreactor cultures (see Figure 3a–c), but simple univariate linear regression to correlate infectious titer and online DO, VCV, and OUR values was not predictive. The concept of utilizing these parameters to predict timing, rather than absolute value, of infectious titer required higher resolution information on viral kinetics. To our knowledge, this was the first continuous TCID50 model generated by measured cell‐specific production determined over discrete time periods (see Figure 2a,b). With this approach, we were able to computationally determine trigger criteria that targeted various biological phenomena occurring in the culture. These biological phenomena included: (1) peak virus production within infected cells and beginning of lysis, (2) peak cell lysis and realized viral titer, and (3) complete release of viral particles (see Supplemental Table 1). The optimal criteria for DO and OUR occurred during the steady increase (DO) or decrease (OUR) of the trend whereas the optimal criteria for VCV occurred when the VCV began to decline. The offset between the PAT harvest trigger and recommended harvest timing ranged from 0.5 to 1.3 days. This intentional offset allows for preparation and operational planning like a traditional time‐based harvest, while having the advantage of being adaptable to variable viral kinetics. Combined with the ability to automate temperature changes with online criteria, the PAT harvest trigger could decrease the temperature of the tank, reducing the loss of viral titer until staff are prepared to harvest. A simple reduction in temperature from 34 to 20°C enabled less than 10% of the product to be lost over a day period, rather than a 30% loss (see Figure 1). For more labile viruses, the benefit would be even greater.

Harvest triggers minimize batch variability and manage process deviations by leveraging process understanding. For control batches, the average peak viral titer was observed at 2.78 days post infection with a standard deviation of 0.49 days (Figure 4a). Harvesting with an online harvest trigger rather than a time‐based harvest method allowed for significantly improved batch consistency (see Figure 4a,b). The DO based harvest trigger reduced the standard deviation of percent maximum yield at harvest by 41%, the OUR based harvest trigger reduced it by 56% and the VCV based harvest trigger reduced it by 71%. On average, the time‐based harvest method triggered harvest when there was 91.4% of peak viral titer in the tank. The PAT harvest triggers increased the average percent of peak viral titer at harvest to 94%–95.5%. While this was not a statistically significant improvement in percent of peak viral titer yield for the baseline batches by Kruskal‐Wallis test and Dunn's multiple comparison test, the reduction in the standard deviation is significant as indicated by the Brown‐Forsythe test of variance. The reduction in harvest titer variability is a significant asset in bioprocess. Predictable batch yields decrease the need for unscheduled and unforeseen batches. Confidence in batch yield and meeting manufacturing timelines increase as batch variability decreases. PAT‐enabled harvest automation has the potential to reduce staffing requirements without risking product loss.

For deviant batches, PAT‐based harvest triggers have the potential to save a batch which would otherwise have too low infectious titer yield to be otherwise used. Furthermore, these triggers have the potential to detect changes in process dynamics with minimal training or optimization. By varying the cell plant density, the timing of peak viral titer can be greatly impacted, with the lowest cell plant density having an average peak viral titer at 4.31 days post infection – over a day and a half past the average peak viral titer timing. A time‐based harvest yielded only 16% of peak possible titer. However, all PAT harvest triggers indicated harvest at or above 87% of peak titer for this condition (average DO: 97.5%, average VCV: 96%, and average OUR: 91%). The implication that inoculating the bioreactor with a variable number of cells could have significant impact on viral kinetics and optimal harvest timing supports the usage of a PAT harvest trigger to avoid a potential 84% product loss (see Figure 5a,b). For the variable cell plant conditions, the harvest triggers successfully predicted harvest timing when the time‐based harvest method did not. Whether due to operational or equipment errors, target inoculation densities can be missed; even in the absence of errors, actual inoculation densities always vary from one batch to the next. Process setpoint ranges, such as inoculation density, can be set narrowly to ensure a given product quality profile, or broadly to ensure process deviations are avoided. Alternatively, PAT‐based harvest triggers can enable process flexibility, as reported by Figure 5b.

When MOI or gassing control strategy was varied, predicted yields of greater than 70% on average for all triggers were still realized–both PAT‐ and time‐based (Figure 5d). This excludes the DO trigger in the alternate gassing control strategy condition, which failed as expected as it relies upon a rise in DO. For these deviant conditions where the timing of peak viral titer was not impacted, the PAT methods were comparable to the time‐based harvest method. The low DO condition's unique gassing strategy caused issues for the DO harvest trigger as it is gassing strategy‐dependent. The DO harvest method requires a consistent gassing strategy, control loop, and scale to be viable from batch to batch. Fortunately, this is unlikely to be a problem for most established manufacturing processes. For more stringent processes requiring two‐sided DO control (upper and lower bound), or during process scale‐up, a DO trigger will be less straightforward. While the DO‐based harvest trigger may not be as widely applicable as the VCV and OUR options, the common availability and usage of DO probes make it ideal for processes that have compatible gassing strategies.

Notably, both the VCV‐ and OUR‐based triggers correctly predicted harvest timing despite a DO control scheme process aberration, providing evidence that significant process deviations can be managed by these tools. Peak viral titer yield varied more for the OUR‐based trigger than for the VCV‐based trigger for the low DO condition. While VCV determination was not directly impacted by the DO control strategy, OUR determination was. Since OUR is calculated by leveraging DO slope (change in DO over time), and DO slope is minimized as it is constantly forced to 20% by the control loop, trigger accuracy suffered. Furthermore, signal to noise was lesser as the DO setpoint was reduced to 20%. Peak density and net growth rate were insensitive to low DO, suggesting that oxygen availability was not rate‐limiting at the observed cell density. Predictably, given the lytic nature of CVA21, the kinetics of cell death correlated well with peak viral titer (and consequently harvest timing).

The tools developed and the approach described to monitor viral kinetics and make mathematically driven harvest decisions may be adaptable to other processes, especially other lytic virus processes. There is potential to adapt the concepts of these triggers to non‐lytic viral hosts by monitoring cell‐specific oxygen uptake rates during the production phase^43^, ^54^ or by monitoring specific dielectric spectroscopic parameters that relate to cell membrane changes associated with viral budding.^55^, ^56^ Indeed, a decrease in cell‐specific OUR or changes in cell membrane properties during the production phase could be used to determine when the cell population has reached peak production or when the cell population is no longer capable of producing functional virus. The DO probe is found in almost all bioreactors, and capacitance is becoming increasingly common within the bioprocess industry.^57^ Where both probes exist, cell‐specific OUR can be determined. The mechanisms that connect these online parameters are applicable to many virus‐mammalian cell culture processes and could be developed/utilized to minimize variability per batch. Table 2 provides a practical summary of the different harvest triggers.

**TABLE 2:** A summary of the pros and cons of each harvest trigger method.

	Pros	Cons
DO trigger‐based harvest	Common probe readily available in most bioreactors No modeling needed to generate online data	Dependent on gassing strategy Scale‐dependent due to differences in surface area to volume ratios of different scale bioreactors
VCV trigger‐based harvest	On‐line cellular volume data has many process applications in growth and viral production Not impacted by gassing/other process parameters Scale independent	Necessity of developing correlative model between off‐line cell counting assays and on‐line capacitance signal
OUR trigger‐based harvest	Able to monitor oxygen consumption on a cellular basis Scale independent Potential insights into oxidative state of cells that otherwise may not be captured	Necessity of determining kLa's experimentally, the viable cellular volume model, and performing relatively complex calculation
Time‐based harvest	Simplest approach No necessary models or probes	Unable to detect lot‐to‐lot variability Unable to react to deviations

## 6 CONCLUSIONS

The ability to track viral kinetics and peak viral titer through dissolved oxygen concentration, viable cell volume, and oxygen uptake rate significantly increased harvest yield consistency. Furthermore, it optimized harvest yield. Increasing harvest titers is one of the most effective means of decreasing cost of goods.^8^ Unfortunately, the analytical time required to support frequent sampling to determine viral kinetics is preclusive. With a few control batches (i.e., minimal analytical support), one can interpolate observed specific productivity and calculate titer to not only provide minute‐by‐minute information, but also develop robust PAT harvest triggers. These harvest triggers not only offer direct value to manufacturing—they also offer valuable process understanding. Additionally, the deviant batch case studies, especially the low inoculation density case study, elucidated the advantages of a harvest trigger over a time‐based harvest. The ubiquity of DO probes theoretically makes the use of harvest triggers accessible to any bioreactor system. For systems where the gassing strategy makes DO‐dependent triggers sub‐optimal or scale independence is desired, the capacitance probe or specific oxygen uptake rate could be employed independently of gassing strategy, giving insight into cell propagation and the viral progression of the culture.

Cell‐specific oxygen uptake rate provided a novel metabolic lens on systems without off‐gas analysis capabilities. Digitization of in‐process data has made process sciences data‐rich and passively collected parameters (e.g., DO) provide ample opportunity to drive in‐process decision making. We propose a trio of online process analytical technologies to maximize the yield and consistency of each batch and further inform process understanding critical to process development.

**NOMENCLATURE**
C: concentration of oxygen
D: viable cell density
k_d_
: viral decay rate
k_L_a: mass transfer coefficient
O: overlay contribution
P: viral infectivity
Q_P_
: specific productivity
q: volumetric OUR
S: sparge contribution
t: time
v: vessel contribution
V: viable cell volume
μ: growth rate

## AUTHOR CONTRIBUTIONS

Neil Templeton and Katherine Forrester conceptualized the work. Katherine Forrester, Marena Trauger, and Neil Templeton performed experiments. Katherine Forrester performed analysis of DO and OUR correlations. Marena Trauger correlated capacitance to VCV, which was further expanded by Thomas R. Blanda. Thomas R. Blanda performed computational modeling for specific productivity, viral titer, and harvest trigger development. Rachel Thompson performed HT viral infectivity assay and wrote up the associated method. Katherine Forrester, Thomas R. Blanda, Marena Trauger, and Neil Templeton wrote the manuscript, which was read, edited, and approved by all authors.

## CONFLICT OF INTEREST STATEMENT

All authors were employees of Merck Sharp & Dohme LLC, a subsidiary of Merck & Co., Inc., Rahway, NJ, USA at the time of study execution. All authors are current or former employees of Merck Sharp & Dohme LLC, a subsidiary of Merck & Co., Inc., Rahway, NJ, USA and may own stock or hold stock options in Merck & Co., Inc., Rahway, NJ, USA.

## Supporting information


**Data S1.** Supporting Information.

**Data S2.** Supporting Information.

**Data S3.** Supporting Information.

## Data Availability

The data that supports the findings of this study are available in the supplementary material of this article.
