# Supplementary material for: Minimizing batch‐to‐batch variability of a live virus vaccine by process analytical technologies
Source: Biotechnol Prog. 2025 May 22;41(5):e70037. doi: 10.1002/btpr.70037 (PMC12531925; doi:10.1002/btpr.70037)
Supplement: Supplementary file 1 — Data S1. Supporting Information. [file BTPR-41-e70037-s003.docx]

**Supplemental**

$$q=\frac{k_{L}a_{o}\left( C_{o,air}{-C}_{v} \right)+k_{L}a_{s,O_{2}}\left( C_{s,O_{2}}-C_{L} \right)+k_{L}a_{s,air}{(C}_{s,air}-C_{v})-\left( \pm e^{-\left( {k_{L}a_{o}+k}_{L}a_{s,O2}+k_{L}a_{s,air} \right)*\left( t-t_{0} \right)} \right)*(k_{L}a_{o}(C_{o,air}-C_{v,0})+k_{L}a_{s,O2}{(C}_{s,O2}-C_{v,0})+k_{L}a_{s,air}(C_{s,air}-C_{v,0}))}{-X\left( \pm e^{-\left( {k_{L}a_{o}+k}_{L}a_{s,O2}+k_{L}a_{s,air} \right)*\left( t-t_{0} \right)}-1 \right)}$$

Supplemental Equation 1: Solved Equation 4 for cellular specific oxygen uptake rate.

$$P=\frac{Q_{p}X_{0}\left( e^{\mu\left( t_{1}-t_{0} \right)}-e^{-k_{d}\left( t_{1}-t_{0} \right)} \right)}{\mu+k_{d}}+P_{0}e^{-k_{d}\left( t_{1}-t_{0} \right)}$$

Supplemental Equation 2: Solved Equation 1 for productivity. The specific productivity (Q_p_) of the culture over periods between infectious titer (P) was calculated using the above equation, utilizing specific productivity (Q_p_), continuous growth rate (µ), a constant decay rate (k_d_), and continuous viable cell volume (X) over time (t).

|  | 1 | 2 | 3 |
| --- | --- | --- | --- |
|  | 1. $DPI>0.5 days$ 2. $\frac{d\left( DO \right)}{dt}>y\frac{\mu M}{day}$   $y=$0,5,15,25,30   1. $DO> {DO}_{min}+x({DO}_{max}-{DO}_{min})$   $x=10, 15, 20, 25, 30, 35, 40\%$ | 1. $DPI>1 day$ 2. $\frac{d(DO)}{dt}>y\frac{\mu M}{day}$   $y=$20,25,35,45,50   1. $DO> {DO}_{min}+x({DO}_{max}-{DO}_{min})$   $x=30, 35, 40, 45, 50, 55,$  $60\%$ | 1. $DPI>1.5 days$ 2. $\frac{d(DO)}{dt}<y\frac{\mu M}{day}$   $y=$10,15,30,35,40   1. $DO> {DO}_{max}(1-x)$   $x=15, 20, 25\%$ |
|  | 1. $DPI>0.5 days$ 2. $\frac{d\left( VCV \right)}{dt}<0$ 3. $\vert\frac{d\left( VCV \right)}{dt}\vert>y\frac{{\mu m}^{3} cell}{mL \times day}$   $y= 2e8,1.5e8,8e7,5e7,1e7$   1. $VCV< {VCV}_{max}-x({VCV}_{max}-{VCV}_{min})$   $x=10, 15, 20, 25, 30, 35, 40\%$ | 1. $DPI>1 day$ 2. $\frac{d\left( VCV \right)}{dt}<0$ 3. $\vert\frac{d\left( VCV \right)}{dt}\vert>y\frac{{\mu m}^{3} cell}{mL \times day}$   $y= 1.5e8,1e8,8e7,$5e7,1e7   1. $VCV< {VCV}_{max}-x({VCV}_{max}-{VCV}_{min})$   $x=30, 35, 40, 45, 50, 55, 60\%$ | 1. $DPI>1.5 days$ 2. $\frac{d\left( VCV \right)}{dt}>y\frac{{\mu m}^{3} cell}{mL \times day}$   $y=$  $-2e8,-1.5e8,-8e7,$  $-5e7,-1e7$   1. $VCV< {VCV}_{min}+x\left( {VCV}_{max}-{VCV}_{min} \right)$   $x=15, 20, 25\%$ |
|  | 1. $DPI>0.5 days$ 2. $\frac{d(OUR)}{dt}<0$ 3. \|$\frac{d\left( OUR \right)}{dt}\vert>y\frac{\mu mol}{{\mu m}^{3} cell \times day}$   $y=1.5e-,1e-6,$  $5e-6,5e-7,8e-6$   1. $OUR< {OUR}_{max}-x({OUR}_{max}-{OUR}_{min})$   $x=10, 15, 20, 25, 30, 35, 40\%$ | 1. $DPI>1 day$ 2. $\frac{d(OUR)}{dt}<0$ 3. \|$\frac{d\left( OUR \right)}{dt}\vert>y\frac{\mu mol}{{\mu m}^{3} cell \times day}$   $y=1e-5,2e-6,$  $3e-6,5e-6,8e-6$   1. $OUR< {OUR}_{max}-x({OUR}_{max}-{OUR}_{min})$   $x=30, 35, 40, 45, 50, 55, 60\%$ | 1. $DPI>1.5 days$ 2. $\frac{d\left( OUR \right)}{dt}>y\frac{\mu mol}{{\mu m}^{3} cell \times day}$   $y=-1e-6,-2e-6,$  -4e-7,-5e-7,-8e-7   1. $OUR< {OUR}_{min}+x({OUR}_{max}-{OUR}_{min})$ 2. $x=15, 20, 25\%$ |

Supplemental Table 1: For each trend, three key areas were identified for mathematical determination and comparison to peak viral potency to determine the best harvest trigger. Figures and tables above show the three trends – DO, VCV, and OUR with the three targeted trigger regions and the trend specific trigger criteria that was tested in a table to the right of the trend.

**Description of colored regions:**

1. GREEN - indicates the beginning of major cellular lysis where DO begins to increase and VCV and OUR begin to decrease,
2. BLUE - indicates the main cell lysis period where virus is being released and DO is increasing at a steady rate while VCV and OUR are decreasing at a steady rate, and
3. PINK - indicates the period where the cells have generally lysed and finished releasing virus causing all trends to level out.

For the x and y values for each trigger, variable values were tested to ensure optimal criteria was tested. For each criteria listed above each x value was tested mathematically with each y value.

*DO*

| 1 | |
| --- | --- |
| Average Peak Potency at Harvest Trigger | Standard Deviation of Trigger |
| \| \%  Slope \| 10 \| 15 \| 20 \| 25 \| 30 \| 35 \| 40 \| \| --- \| --- \| --- \| --- \| --- \| --- \| --- \| --- \| \| 0 \| 81.7 \| 81 \| 82 \| 82.9 \| 87.9 \| 88.1 \| 93.2 \| \| 5 \| 83.5 \| 82.5 \| 82 \| 82.8 \| 87.9 \| 88.1 \| 93.2 \| \| 15 \| 85 \| 84 \| 84.3 \| 84.4 \| 88 \| 88.1 \| 93.2 \| \| 25 \| 84.6 \| 83.8 \| 84.8 \| 86.8 \| 88.7 \| 91.2 \| 93.4 \| \| 30 \| 85.7 \| 85 \| 87.2 \| 89.5 \| 88.5 \| 92.3 \| 94.3 \| | \| \%  Slope \| 10 \| 15 \| 20 \| 25 \| 30 \| 35 \| 40 \| \| --- \| --- \| --- \| --- \| --- \| --- \| --- \| --- \| \| 0 \| 12.7 \| 13.1 \| 12.7 \| 13.4 \| 13.3 \| 14.1 \| 7.34 \| \| 5 \| 11.8 \| 12.2 \| 12.9 \| 13.7 \| 13.4 \| 14.3 \| 7.34 \| \| 15 \| 12.1 \| 12.5 \| 12.4 \| 11.9 \| 14.1 \| 14.3 \| 7.34 \| \| 25 \| 14.5 \| 15.1 \| 15 \| 15.3 \| 15.8 \| 8.17 \| 7.12 \| \| 30 \| 15.6 \| 16.2 \| 16.1 \| 16.6 \| 17 \| 8.17 \| 4.96 \| |
| 2 | |
| Average Percent Peak Potency at Harvest Trigger | Standard Deviation of Trigger |
| \| \%  Slope \| 30 \| 35 \| 40 \| 45 \| 50 \| 55 \| 60 \| \| --- \| --- \| --- \| --- \| --- \| --- \| --- \| --- \| \| 20 \| 91.4 \| 91.8 \| 93.3 \| 94 \| 94.7 \| 95.2 \| 95.2 \| \| 25 \| 91.4 \| 91.8 \| 93.4 \| **94** \| 95.1 \| 95.2 \| 95.2 \| \| 35 \| 92.3 \| 93.8 \| 94.5 \| 93.9 \| 95.3 \| 95.2 \| 95.2 \| \| 45 \| 94.1 \| 94.8 \| 95 \| 95.2 \| 95.7 \| 88 \| 88.1 \| \| 50 \| 93.9 \| 94.5 \| 88.3 \| 85 \| 84.9 \| 81.5 \| 81.5 \| | \| \%  Slope \| 30 \| 35 \| 40 \| 45 \| 50 \| 55 \| 60 \| \| --- \| --- \| --- \| --- \| --- \| --- \| --- \| --- \| \| 20 \| 9.69 \| 8.23 \| 7.21 \| 5.12 \| 4.9 \| 4.02 \| 4.27 \| \| 25 \| 9.66 \| 8.23 \| 7.12 \| **5.17** \| 3.72 \| 4.02 \| 4.13 \| \| 35 \| 8.16 \| 7.7 \| 5.17 \| 5.85 \| 3.89 \| 4.3 \| 4.38 \| \| 45 \| 5.03 \| 5.19 \| 5.14 \| 5.44 \| 4.2 \| 24.8 \| 24.9 \| \| 50 \| 5.65 \| 5.3 \| 24.7 \| 24.7 \| 24.7 \| 24.7 \| 24.6 \| |
| 3 | |
| Average Percent Peak Potency at Harvest Trigger | Standard Deviation of Trigger |
| \| \%  Slope \| 10 \| 15 \| 20 \| 25 \| 30 \| 35 \| \| --- \| --- \| --- \| --- \| --- \| --- \| --- \| \| 10 \| 94.3 \| 93.5 \| 88.8 \| 87.7 \| 86.5 \| 82.7 \| \| 15 \| 95.1 \| 94.1 \| 89.7 \| 88.1 \| 86.1 \| 83.5 \| \| 30 \| 94.8 \| 93.2 \| 91.2 \| 88.7 \| 87.2 \| 86.7 \| \| 35 \| 94.4 \| 94.1 \| 91.9 \| 89.5 \| 88.1 \| 87.3 \| \| 40 \| 94.2 \| 94.7 \| 93.3 \| 91.4 \| 89.7 \| 89.3 \| | \| \%  Slope \| 10 \| 15 \| 20 \| 25 \| 30 \| 35 \| \| --- \| --- \| --- \| --- \| --- \| --- \| --- \| \| 10 \| 5.51 \| 9.74 \| 14.6 \| 14.2 \| 17 \| 16.3 \| \| 15 \| 6.26 \| 7.5 \| 13.1 \| 13.9 \| 15.1 \| 13.7 \| \| 30 \| 5.73 \| 7.12 \| 10.7 \| 11.2 \| 12 \| 11.7 \| \| 35 \| 5.37 \| 6.69 \| 9.71 \| 10.5 \| 11.1 \| 11.3 \| \| 40 \| 5.34 \| 5.99 \| 9.11 \| 9.1 \| 9.72 \| 10.5 \| |

*VCV*

| 1 | |
| --- | --- |
| Average Peak Potency at Harvest Trigger | Standard Deviation of Trigger |
| \| \%  Slope \| 10 \| 15 \| 20 \| 25 \| 30 \| 35 \| 40 \| \| --- \| --- \| --- \| --- \| --- \| --- \| --- \| --- \| \| -2.0E+8 \| 94.5 \| 94.7 \| 94.7 \| 94.7 \| 94.8 \| 94.8 \| 94.7 \| \| -1.5E+8 \| **95.3** \| 95.2 \| 95.0 \| 95.0 \| 94.9 \| 94.8 \| 94.7 \| \| -8.0E+7 \| 94.8 \| 95.0 \| 95.1 \| 95.0 \| 94.9 \| 94.8 \| 94.7 \| \| -5.0E+7 \| 94.8 \| 95.0 \| 95.1 \| 95.0 \| 94.9 \| 94.8 \| 94.7 \| \| -1.0E+7 \| 94.9 \| 95.0 \| 95.1 \| 95.0 \| 94.9 \| 94.8 \| 94.7 \| | \| \%  Slope \| 10 \| 15 \| 20 \| 25 \| 30 \| 35 \| 40 \| \| --- \| --- \| --- \| --- \| --- \| --- \| --- \| --- \| \| -2.0E+8 \| 0.41 \| 0.39 \| 0.39 \| 0.38 \| 0.38 \| 0.38 \| 0.38 \| \| -1.5E+8 \| **0.37** \| 0.38 \| 0.38 \| 0.38 \| 0.38 \| 0.38 \| 0.38 \| \| -8.0E+7 \| 0.41 \| 0.39 \| 0.38 \| 0.37 \| 0.38 \| 0.38 \| 0.38 \| \| -5.0E+7 \| 0.41 \| 0.39 \| 0.38 \| 0.37 \| 0.38 \| 0.38 \| 0.38 \| \| -1.0E+7 \| 0.40 \| 0.39 \| 0.38 \| 0.37 \| 0.38 \| 0.38 \| 0.38 \| |
| 2 | |
| Average Percent Peak Potency at Harvest Trigger | Standard Deviation of Trigger |
| \| \%  Slope \| 30 \| 35 \| 40 \| 45 \| 50 \| 55 \| 60 \| \| --- \| --- \| --- \| --- \| --- \| --- \| --- \| --- \| \| -1.5E+8 \| 94.9 \| 94.8 \| 94.7 \| 94.6 \| 94.5 \| 94.5 \| 94.4 \| \| -1.0E+8 \| 94.9 \| 94.8 \| 94.7 \| 94.6 \| 94.5 \| 94.5 \| 94.4 \| \| -8.0E+7 \| 94.9 \| 94.8 \| 94.7 \| 94.6 \| 94.5 \| 94.5 \| 94.4 \| \| -5.0E+7 \| 94.9 \| 94.8 \| 94.7 \| 94.6 \| 94.5 \| 94.5 \| 94.4 \| \| -1.0E+7 \| 94.9 \| 94.8 \| 94.7 \| 94.6 \| 94.5 \| 94.5 \| 94.4 \| | \| \%  Slope \| 30 \| 35 \| 40 \| 45 \| 50 \| 55 \| 60 \| \| --- \| --- \| --- \| --- \| --- \| --- \| --- \| --- \| \| -1.5E+8 \| 0.38 \| 0.38 \| 0.38 \| 0.38 \| 0.39 \| 0.39 \| 0.39 \| \| -1.0E+8 \| 0.38 \| 0.38 \| 0.38 \| 0.38 \| 0.39 \| 0.39 \| 0.39 \| \| -8.0E+7 \| 0.38 \| 0.38 \| 0.38 \| 0.38 \| 0.39 \| 0.39 \| 0.39 \| \| -5.0E+7 \| 0.38 \| 0.38 \| 0.38 \| 0.38 \| 0.39 \| 0.39 \| 0.39 \| \| -1.0E+7 \| 0.38 \| 0.38 \| 0.38 \| 0.38 \| 0.39 \| 0.39 \| 0.39 \| |
| 3 | |
| Average Percent Peak Potency at Harvest Trigger | Standard Deviation of Trigger |
| \| \%  Slope \| 10 \| 15 \| 20 \| 25 \| 30 \| 35 \| \| --- \| --- \| --- \| --- \| --- \| --- \| --- \| \| -2.0E+8 \| 93.0 \| 93.9 \| 94.0 \| 94.1 \| 94.1 \| 94.1 \| \| -1.5E+8 \| 92.9 \| 94.1 \| 94.1 \| 94.1 \| 94.1 \| 94.1 \| \| -8.0E+7 \| 92.6 \| 93.7 \| 93.7 \| 93.7 \| 93.7 \| 93.7 \| \| -5.0E+7 \| 92.8 \| 94.0 \| 94.0 \| 94.0 \| 94.0 \| 94.0 \| \| -1.0E+7 \| 92.0 \| 92.8 \| 92.8 \| 92.8 \| 92.8 \| 92.8 \| | \| \%  Slope \| 10 \| 15 \| 20 \| 25 \| 30 \| 35 \| \| --- \| --- \| --- \| --- \| --- \| --- \| --- \| \| -2.0E+8 \| 0.42 \| 0.41 \| 0.41 \| 0.40 \| 0.40 \| 0.40 \| \| -1.5E+8 \| 0.42 \| 0.41 \| 0.41 \| 0.41 \| 0.41 \| 0.41 \| \| -8.0E+7 \| 0.43 \| 0.42 \| 0.42 \| 0.42 \| 0.42 \| 0.42 \| \| -5.0E+7 \| 0.42 \| 0.42 \| 0.42 \| 0.42 \| 0.42 \| 0.42 \| \| -1.0E+7 \| 0.46 \| 0.46 \| 0.46 \| 0.46 \| 0.46 \| 0.46 \| |

*OUR*

| 1 | |
| --- | --- |
| Average Peak Potency at Harvest Trigger | Standard Deviation of Trigger |
| \| \%  Slope \| 10 \| 15 \| 20 \| 25 \| 30 \| 35 \| 40 \| \| --- \| --- \| --- \| --- \| --- \| --- \| --- \| --- \| \| -8.0E-6 \| 79.2 \| 79.3 \| 79.3 \| 79.3 \| 79.3 \| 80.8 \| 80.7 \| \| -5.0E-6 \| 89.1 \| 89.2 \| 89.5 \| 89.7 \| 90.0 \| 90.2 \| 90.5 \| \| -1.5E-6 \| 82.4 \| 82.0 \| 81.7 \| 81.3 \| 80.9 \| 80.6 \| 80.3 \| \| -1.0E-6 \| 80.1 \| 79.7 \| 79.4 \| 79.0 \| 78.7 \| 78.4 \| 78.1 \| \| 5.0E-7 \| 85.7 \| 85.5 \| 85.3 \| 85.0 \| 84.9 \| 84.7 \| 84.6 \| | \| \%  Slope \| 10 \| 15 \| 20 \| 25 \| 30 \| 35 \| 40 \| \| --- \| --- \| --- \| --- \| --- \| --- \| --- \| --- \| \| -8.0E-6 \| 0.91 \| 0.91 \| 0.91 \| 0.91 \| 0.91 \| 0.91 \| 0.9 \| \| -5.0E-6 \| 0.71 \| 0.71 \| 0.71 \| 0.71 \| 0.71 \| 0.71 \| 0.71 \| \| -1.5E-6 \| 0.73 \| 0.74 \| 0.76 \| 0.77 \| 0.79 \| 0.8 \| 0.82 \| \| -1.0E-6 \| 0.75 \| 0.77 \| 0.78 \| 0.8 \| 0.82 \| 0.83 \| 0.84 \| \| -5.0E-7 \| 0.61 \| 0.61 \| 0.62 \| 0.64 \| 0.65 \| 0.66 \| 0.66 \| |
| 2 | |
| Average Percent Peak Potency at Harvest Trigger | Standard Deviation of Trigger |
| \| \%  Slope \| 30 \| 35 \| 40 \| 45 \| 50 \| 55 \| 60 \| \| --- \| --- \| --- \| --- \| --- \| --- \| --- \| --- \| \| -1.0E-5 \| 66.7 \| 54.3 \| 54.3 \| 54.3 \| 53.4 \| 50 \| 50 \| \| -8.0E-6 \| 79.9 \| 81.3 \| 81.3 \| 81.3 \| 72.8 \| 74.5 \| 68.7 \| \| -5.0E-6 \| 90.1 \| 90.4 \| 90.8 \| 90.3 \| 90.5 \| 90.2 \| 90.6 \| \| -3.0E-6 \| 92.8 \| 92.6 \| 92.4 \| 92.3 \| 92.1 \| **94.7** \| 94.3 \| \| -2.0E-6 \| 81.8 \| 81.3 \| 80.8 \| 80.5 \| 81.2 \| 83 \| 82.3 \| | \| \%  Slope \| 30 \| 35 \| 40 \| 45 \| 50 \| 55 \| 60 \| \| --- \| --- \| --- \| --- \| --- \| --- \| --- \| --- \| \| -1.0E-5 \| 0.92 \| 1.01 \| 1.01 \| 1 \| 1.05 \| 0.95 \| 0.95 \| \| -8.0E-6 \| 0.86 \| 0.86 \| 0.86 \| 0.86 \| 0.92 \| 0.85 \| 0.85 \| \| -5.0E-6 \| 0.67 \| 0.67 \| 0.67 \| 0.67 \| 0.67 \| 0.65 \| 0.64 \| \| -3.0E-6 \| 0.55 \| 0.55 \| 0.55 \| 0.55 \| 0.56 \| **0.48** \| 0.49 \| \| -2.0E-6 \| 0.67 \| 0.69 \| 0.7 \| 0.71 \| 0.73 \| 0.68 \| 0.69 \| |
| 3 | |
| Average Percent Peak Potency at Harvest Trigger | Standard Deviation of Trigger |
| \| \%  Slope \| 10 \| 15 \| 20 \| 25 \| 30 \| 35 \| \| --- \| --- \| --- \| --- \| --- \| --- \| --- \| \| -2.0E-6 \| 51.8 \| 63.4 \| 77.9 \| 84.3 \| 84.7 \| 85 \| \| -1.0E-6 \| 50.8 \| 62.3 \| 77 \| 83.2 \| 83.3 \| 83.4 \| \| -8.0E-7 \| 50.6 \| 62.2 \| 76.6 \| 82.7 \| 82.8 \| 83 \| \| -5.0E-7 \| 49.6 \| 60.8 \| 75.3 \| 81.5 \| 81.5 \| 81.7 \| \| -4.0E-7 \| 49.5 \| 60.7 \| 69.3 \| 81.5 \| 81.5 \| 81.6 \| | \| \%  Slope \| 10 \| 15 \| 20 \| 25 \| 30 \| 35 \| \| --- \| --- \| --- \| --- \| --- \| --- \| --- \| \| -2.0E-6 \| 0.65 \| 0.93 \| 0.68 \| 0.59 \| 0.58 \| 0.57 \| \| -1.0E-6 \| 0.69 \| 0.94 \| 0.7 \| 0.63 \| 0.63 \| 0.63 \| \| -8.0E-7 \| 0.69 \| 0.94 \| 0.7 \| 0.64 \| 0.64 \| 0.63 \| \| -5.0E-7 \| 0.72 \| 0.95 \| 0.72 \| 0.66 \| 0.66 \| 0.66 \| \| -4.0E-7 \| 0.72 \| 0.95 \| 0.7 \| 0.67 \| 0.67 \| 0.67 \| |

Supplemental Table 2: Comparison of each of the trigger criteria to maximize yield and reduce standard deviation in time from the maximum titer.
