# Supplementary material for: Minimizing batch‐to‐batch variability of a live virus vaccine by process analytical technologies
Source: Biotechnol Prog. 2025 May 22;41(5):e70037. doi: 10.1002/btpr.70037 (PMC12531925; doi:10.1002/btpr.70037)
Supplement: Supplementary file 2 — Data S2. Supporting Information. [file BTPR-41-e70037-s001.pdf]

```
rm(list=ls())


#### Loading Necessary Packages ####

library(rstudioapi)

library(readxl)

library(tidyverse)

library(dplyr)

library(janitor)

library(fuzzyjoin)

library(lubridate)

library(readr)

library(writexl)

library(openxlsx)

library(stringr)

library(tidyr)

library(tibble)

library(ggplot2)

library(ggpubr)

library(gganimate)


#### Basic Setup ####

file<-selectFile()

OUR_Data<-read_excel(file,sheet="Sheet1",guess_max=10000)

Batch_IDs<-unique(OUR_Data[,c(3)])

Control_Batch<-Batch_IDs%>%filter(grepl("3L-018 A1|3L-018 A2|3L-020 A1|3L-020
A2|3L-020 A3|3L-020 A4|3L-023 A1|3L-023 A2|3L-023 D1|3L-023 D3|3L-024 A2|3L-024
```

```
B1|3L-024 B2|3L-026 D1|3L-026 D2|3L-026 D3|3L-026 D4",`Batch ID`))

Non_Control_Batch<-Batch_IDs%>%filter(!grepl("3L-018 A1|3L-018 A2|3L-020 A1|3L-020
A2|3L-020 A3|3L-020 A4|3L-023 A1|3L-023 A2|3L-023 D1|3L-023 D2|3L-023 D3|3L-024
A2|3L-024 B1|3L-024 B2|3L-026 D1|3L-026 D2|3L-026 D3|3L-026 D4",`Batch ID`))

Num_Batch_ID<-nrow(Control_Batch)



#### Extend Theoretical VII ####

Death_Rate<-0.36

extendo<-list()

for (z in 1:nrow(Batch_IDs))

{

  h<-OUR_Data%>%filter(`Batch ID`==as.character(Batch_IDs[c(z),]))

  Temp_End<-tail(h,n=1)

  Row_Store<-nrow(h)

  if(z<=2)

  {

    Row_Add<-as.numeric(round((6-Temp_End[,c("DPI")])*24*60/3,0))

    Row_Add<-data.frame(matrix(ncol=ncol(h),nrow=Row_Add))

    Time_Step<-3/24/60

    h<-rbind(h,setNames(Row_Add,names(h)))

    for (r in (Row_Store+1):nrow(h))

    {

      h[c(r),c("DPP","DPI")]<-h[c(r-1),c("DPP","DPI")]+Time_Step

      h[c(r),c("Batch","BR","Batch
ID","Infection?")]<-h[c(r-1),c("Batch","BR","Batch ID","Infection?")]

    }

    Row_Store<-nrow(filter(h,DPI<=tail(filter(h,!is.na(VII))$DPI,n=1)))
```

```
    for (r in (Row_Store+1):nrow(h))

    {

h[c(r),c("Step_100_row_VII")]<-h[c(r-1),c("Step_100_row_VII")]*exp(-Death_Rate*(h[c(
r),c("DPI")]-h[c(r-1),c("DPI")]))

    }

  }else

  {

    Row_Add<-as.numeric(round((6-Temp_End[,c("DPI")])*24*60,0))

    Row_Add<-data.frame(matrix(ncol=ncol(h),nrow=Row_Add))

    Time_Step<-1/24/60

    h<-rbind(h,setNames(Row_Add,names(h)))

    for (r in (Row_Store+1):nrow(h))

    {

      h[c(r),c("DPP","DPI")]<-h[c(r-1),c("DPP","DPI")]+Time_Step

      h[c(r),c("Batch","BR","Batch
ID","Infection?")]<-h[c(r-1),c("Batch","BR","Batch ID","Infection?")]

    }

    Row_Store<-nrow(filter(h,DPI<=tail(filter(h,!is.na(VII))$DPI,n=1)))

    for (r in (Row_Store+1):nrow(h))

    {

h[c(r),c("Step_100_row_VII")]<-as.numeric(h[c(r-1),c("Step_100_row_VII")])*exp(-Deat
h_Rate*(as.numeric(h[c(r),c("DPI")])-as.numeric(h[c(r-1),c("DPI")])))

    }

  }

  Batch<-paste(Batch_IDs[c(z),],"",sep="")
```

```
  extendo[[Batch]]<-h

}


OUR_Data_Extended<-do.call("rbind",extendo)

write_xlsx(OUR_Data_Extended,"OUR_Data_Extended_TRB_07Jun2023.xlsx")


#### VII Percentile Calculations ####

VII_Percentiles<-data.frame(array(c(95,90,85,80,75,70,65)))

colnames(VII_Percentiles)<-c("Percentiles")

Peak_VII<-as.data.frame(matrix(ncol=5))

colnames(Peak_VII)<-c("Peak_Cont_VII_DPI","Peak_Cont_VII","Peak_VII_DPI","Peak_VII",
"Batch_ID")

Percentile_Store<-data.frame(matrix(ncol=1))


for (l in 1:Num_Batch_ID)

{

  h<-filter(OUR_Data_Extended,`Batch
ID`==as.character(Control_Batch[l,])&`Infection?`=="INFECTION")


Temp_Filter_Cont_VII_Full<-h%>%filter(Step_100_row_VII==max(Step_100_row_VII,na.rm=T
RUE))

  Temp_Filter_VII_Full<-h%>%filter(VII==max(VII,na.rm=TRUE))

  Peak_VII[c(l),c(1:2)]<-Temp_Filter_Cont_VII_Full[,c("DPI","Step_100_row_VII")]

  Peak_VII[c(l),c(3:5)]<-Temp_Filter_VII_Full[,c("DPI","VII","Batch ID")]

  if (l==2)

  {

    Blank_rows<-data.frame(matrix(ncol=29,nrow=(nrow(Control_Batch)-1)))
```

```
Percentile_Store<-rbind(Percentile_Store,setNames(Blank_rows,names(Percentile_Store)
))

  }

  for (i in 1:nrow(VII_Percentiles))

  {

Temp_Percentile_Minus<-h%>%filter(Step_100_row_VII<(Peak_VII[c(l),c(2)]-((100-VII_Pe
rcentiles[c(i),])/100)*Peak_VII[c(l),c(2)])&DPI<Peak_VII[c(l),c(1)])

Temp_Percentile_Plus<-h%>%filter(Step_100_row_VII<(Peak_VII[c(l),c(2)]-((100-VII_Per
centiles[c(i),])/100)*Peak_VII[c(l),c(2)])&DPI>Peak_VII[c(l),c(1)])


    if (l==1)

    {

      Varname_Plus_DPI<-paste("+",VII_Percentiles[c(i),],"th"," DPI",sep="")

      Varname_Minus_DPI<-paste("-",VII_Percentiles[c(i),],"th"," DPI",sep="")

      Varname_Plus_Value<-paste("+",VII_Percentiles[c(i),],"th"," Value",sep="")

      Varname_Minus_Value<-paste("-",VII_Percentiles[c(i),],"th"," Value",sep="")

Percentile_Store[[Varname_Minus_DPI]]<-tail(Temp_Percentile_Minus[,c("DPI")],n=1)

Percentile_Store[[Varname_Minus_Value]]<-tail(Temp_Percentile_Minus[,c("Step_100_row
_VII")],n=1)

      Percentile_Store[[Varname_Plus_DPI]]<-Temp_Percentile_Plus[c(1),c("DPI")]

Percentile_Store[[Varname_Plus_Value]]<-Temp_Percentile_Plus[c(1),c("Step_100_row_VI
I")]

    }else

    {

      Varname_Plus_DPI<-paste("+",VII_Percentiles[c(i),],"th"," DPI",sep="")
```

```
      Varname_Minus_DPI<-paste("-",VII_Percentiles[c(i),],"th"," DPI",sep="")

      Varname_Plus_Value<-paste("+",VII_Percentiles[c(i),],"th"," Value",sep="")

      Varname_Minus_Value<-paste("-",VII_Percentiles[c(i),],"th"," Value",sep="")

Percentile_Store[c(l),c(Varname_Minus_DPI)]<-tail(Temp_Percentile_Minus[,c("DPI")],n
=1)

Percentile_Store[c(l),c(Varname_Minus_Value)]<-tail(Temp_Percentile_Minus[,c("Step_1
00_row_VII")],n=1)

Percentile_Store[c(l),c(Varname_Plus_DPI)]<-Temp_Percentile_Plus[c(1),c("DPI")]

Percentile_Store[c(l),c(Varname_Plus_Value)]<-Temp_Percentile_Plus[c(1),c("Step_100_
row_VII")]

    }

  }

}

Peak_VII<-cbind(Peak_VII,Percentile_Store[,-c(1)])

#### Trigger 1 Calculations ####

Value_Trigger<-data.frame(array(c(10,15,20,25,30,35,40)))

colnames(Value_Trigger)<-c("Trigger Value Percent")

DPI_Trigger<-0.5

Derivative_Trigger<-matrix(c(0, -5e7, -5e-7,

                             5, -1e7, -1e-6,

                             15, -2e8, -5e-6,

                             25, -1.5e8, -8e-6,
```

```
                              30, -8e7, -1.5e-6),nrow=5,ncol=3,byrow=TRUE)

colnames(Derivative_Trigger)<-c("DO_Derivative","VCV_Derivative","OUR_Derivative")


DO_x_Data<-as.data.frame(matrix(ncol=Num_Batch_ID))

DO_y_Data<-as.data.frame(matrix(ncol=Num_Batch_ID))

DO_DPI_Trigger_Full_1<-as.data.frame(matrix(ncol=4))

colnames(DO_DPI_Trigger_Full_1)<-c("DO_DPI_Trigger","DO%_Trigger","dDO_Trigger","Bat
ch_ID")


VCV_x_Data<-as.data.frame(matrix(ncol=Num_Batch_ID))

VCV_y_Data<-as.data.frame(matrix(ncol=Num_Batch_ID))

VCV_DPI_Trigger_Full_1<-as.data.frame(matrix(ncol=4))

colnames(VCV_DPI_Trigger_Full_1)<-c("VCV_DPI_Trigger","VCV_Trigger","dVCV_Trigger","
Batch_ID")


OUR_x_Data<-as.data.frame(matrix(ncol=Num_Batch_ID))

OUR_y_Data<-as.data.frame(matrix(ncol=Num_Batch_ID))

OUR_DPI_Trigger_Full_1<-as.data.frame(matrix(ncol=4))

colnames(OUR_DPI_Trigger_Full_1)<-c("OUR_DPI_Trigger","OUR_Trigger","dOUR_Trigger","
Batch_ID")


VII_x_Data<-as.data.frame(matrix(ncol=Num_Batch_ID))

VII_y_Data<-as.data.frame(matrix(ncol=Num_Batch_ID))


for (a in 1:nrow(Value_Trigger))

{

  for (i in 1:nrow(Derivative_Trigger))
```

```
  {
    for (l in 1:Num_Batch_ID)
    {
      h<-filter(OUR_Data_Extended,`Batch
ID`==as.character(Control_Batch[l,])&`Infection?`=="INFECTION")
      f<-filter(OUR_Data_Extended,`Batch ID`==as.character(Control_Batch[l,]))
      if (a==1&&i==1)
      {
        n=nrow(h)
        DO_x_Data[1:n,c(l)]<-h[,"DPI"]
        DO_y_Data[1:n,c(l)]<-h[,"Avg DO hr (%)"]

        VCV_x_Data[1:n,c(l)]<-h[,"DPI"]
        VCV_y_Data[1:n,c(l)]<-h[,"Avg VCV hr (um3 cells)"]

        OUR_x_Data[1:n,c(l)]<-h[,"DPI"]
        OUR_y_Data[1:n,c(l)]<-h[,"Avg OUR hr (/um3 cells) min"]

        VII_x_Data[1:n,c(l)]<-h[,"DPI"]
        VII_y_Data[1:n,c(l)]<-h[,"Step_100_row_VII"]
      }

      #setting up empty matrices and filling them with batch data
      DO_Data_Trim<-as.data.frame(matrix(ncol=3))
      colnames(DO_Data_Trim)<-c("DPI","DO%","dDO%")
```

```
	DO_Data_Trim[1:nrow(f),]<-f[,(c("DPI","Avg DO hr (%)","dDO (%)"))]


	VCV_Data_Trim<-as.data.frame(matrix(ncol=3))

	colnames(VCV_Data_Trim)<-c("DPI","VCV","dVCV")

	VCV_Data_Trim[1:nrow(f),]<-f[,(c("DPI","Avg VCV hr (um3 cells)","dVCV (um3 
cells)"))]


	OUR_Data_Trim<-as.data.frame(matrix(ncol=3))

	colnames(OUR_Data_Trim)<-c("DPI","OUR","dOUR")

	OUR_Data_Trim[1:nrow(f),]<-f[,(c("DPI","Avg OUR hr (/um3 cells) min","dOUR 
(/um3 cells) min" ))]


	#Max and Min DO calculations


Max_DO<-DO_Data_Trim%>%filter(DPI<(-0.5))%>%filter(`DO%`==max(`DO%`,na.rm=TRUE))

	Max_DO<-Max_DO[,c(2)]

	# if (round(Max_DO[,c(1)],0)==(-3))

	# {

	#   Max_DO<-Max_DO[,c(2)]

	# }else

	# {

	# 
Max_DO<-DO_Data_Trim%>%filter(DPI>3&`dDO%`<10)%>%filter(`DO%`==max(`DO%`,na.rm=TRUE)
)

	#   Max_DO<-Max_DO[,c(2)]

	#   if (is_empty(Max_DO))

	#   {

	# 
```

```
Max_DO<-DO_Data_Trim%>%filter(DPI>2)%>%filter(`DO%`==max(`DO%`,na.rm=TRUE))

      #     Max_DO<-Max_DO[,c(2)]

      #   }

      # }

Min_DO<-DO_Data_Trim%>%filter(DPI<(-0.1)&`DO%`>0)%>%filter(`DO%`==min(`DO%`,na.rm=TR
UE))

      Min_DO<-Min_DO[,c(2)]



      #Max and min VCV calculations


Max_VCV<-VCV_Data_Trim%>%filter(DPI<3.5&DPI>0.5)%>%filter(VCV==max(VCV,na.rm=TRUE))

      Max_VCV<-Max_VCV[,c(2)]

      Min_VCV<-VCV_Data_Trim%>%filter(DPI<(-1))%>%filter(VCV==min(VCV,na.rm=TRUE))

      Min_VCV<-Min_VCV[,c(2)]

      # if (round(Min_VCV[,c(1)],0)==(-3))

      # {

      #   Min_VCV<-Min_VCV[,c(2)]

      # }else

      # {

      #
Min_VCV<-VCV_Data_Trim%>%filter(DPI>2.5&dVCV>(-1e7)|DPI<(-1))%>%filter(VCV==min(VCV,
na.rm=TRUE))

      #   Min_VCV<-Min_VCV[,c(2)]

      #   if (is_empty(Min_VCV))

      #   {

      #     Min_VCV<-VCV_Data_Trim%>%filter()

      #   }
```

```
# }

#Max and min OUR calculations

Max_OUR<-OUR_Data_Trim%>%filter(DPI<(-0.5))%>%filter(OUR==max(OUR,na.rm=TRUE))

Max_OUR<-Max_OUR[,c(2)]

# if(round(Max_OUR[,c(1)],1)<3&round(Max_OUR[,c(1)],1)>0)

# {

#   Max_OUR<-Max_OUR[,c(2)]

# }else

# {

# 
Max_OUR<-OUR_Data_Trim%>%filter(DPI>0|DPI<(-0.5))%>%filter(DPI<3&dOUR<1e-8)%>%filter
(OUR==max(OUR,na.rm=TRUE))

#   Max_OUR<-Max_OUR[,c(2)]

#   if(is_empty(Max_OUR))

#   {

#     Max_OUR<-OUR_Data_Trim%>%filter()

#   }

# }

Min_OUR<-OUR_Data_Trim%>%filter(DPI<(-1))%>%filter(OUR==min(OUR,na.rm=TRUE))

Min_OUR<-Min_OUR[,c(2)]

# if (round(Min_OUR[,c(1)],0)==(-3))

# {

#   Min_OUR<-Min_OUR[,c(2)]

# }else

# {
```

```
      #
Min_OUR<-OUR_Data_Trim%>%filter(DPI>2.5&dOUR>(-1e-8)|DPI<(-1))%>%filter(OUR==min(OUR
,na.rm=TRUE))

      #   Min_OUR<-Min_OUR[,c(2)]

      #   if (is_empty(Min_OUR))

      #   {

      #     Min_OUR<-OUR_Data_Trim%>%filter()

      #   }

      # }


      #Filtering for Trigger


Temp_Filter_DO_Full<-DO_Data_Trim%>%filter(DPI>DPI_Trigger)%>%filter(`dDO%`>Derivati
ve_Trigger[i,1])%>%filter(`DO%`>Min_DO+(Max_DO-Min_DO)*(Value_Trigger[c(a),]/100))


Temp_Filter_VCV_Full<-VCV_Data_Trim%>%filter(DPI>DPI_Trigger)%>%filter(dVCV<Derivati
ve_Trigger[i,2])%>%filter(VCV<Max_VCV-(Max_VCV-Min_VCV)*(Value_Trigger[c(a),]/100))


Temp_Filter_OUR_Full<-OUR_Data_Trim%>%filter(DPI>DPI_Trigger)%>%filter(dOUR<Derivati
ve_Trigger[i,3])%>%filter(OUR<Max_OUR-(Max_OUR-Min_OUR)*(Value_Trigger[c(a),]/100))




      #Storing Trigger Data

      if (a==1&&i==1)

      {

        DO_DPI_Trigger_Full_1[c(l),c(1:3)]<-Temp_Filter_DO_Full[c(1),]

        DO_DPI_Trigger_Full_1[c(l),c(4)]<-Control_Batch[l,]


DO_DPI_Trigger_Full_1<-DO_DPI_Trigger_Full_1%>%mutate(DO_Label="DO_DPI_Trigger_1",`T
rigger_Value_%`=Value_Trigger[c(a),],`Slope_Value`=Derivative_Trigger[i,1])
```

```
        DO_row<-nrow(DO_DPI_Trigger_Full_1)


        VCV_DPI_Trigger_Full_1[c(l),c(1:3)]<-Temp_Filter_VCV_Full[c(1),]

        VCV_DPI_Trigger_Full_1[c(l),c(4)]<-Control_Batch[l,]

VCV_DPI_Trigger_Full_1<-VCV_DPI_Trigger_Full_1%>%mutate(VCV_Label="VCV_DPI_Trigger_1
",`Trigger_Value_%`=Value_Trigger[c(a),],`Slope_Value`=Derivative_Trigger[i,2])

        VCV_row<-nrow(VCV_DPI_Trigger_Full_1)


        OUR_DPI_Trigger_Full_1[c(l),c(1:3)]<-Temp_Filter_OUR_Full[c(1),]

        OUR_DPI_Trigger_Full_1[c(l),c(4)]<-Control_Batch[l,]

OUR_DPI_Trigger_Full_1<-OUR_DPI_Trigger_Full_1%>%mutate(OUR_Label="OUR_DPI_Trigger_1
",`Trigger_Value_%`=Value_Trigger[c(a),],`Slope_Value`=Derivative_Trigger[i,3])

        OUR_row<-nrow(OUR_DPI_Trigger_Full_1)


      }else

      {

        DO_DPI_Trigger_Full_1[c(DO_row+l),c(1:3)]<-Temp_Filter_DO_Full[c(1),]

        DO_DPI_Trigger_Full_1[c(DO_row+l),c(4)]<-Control_Batch[l,]

        DO_DPI_Trigger_Full_1[c(DO_row+l),c(5)]<-c("DO_DPI_Trigger_1")

        DO_DPI_Trigger_Full_1[c(DO_row+l),c(6)]<-Value_Trigger[c(a),]

        DO_DPI_Trigger_Full_1[c(DO_row+l),c(7)]<-Derivative_Trigger[i,1]


        VCV_DPI_Trigger_Full_1[c(VCV_row+l),c(1:3)]<-Temp_Filter_VCV_Full[c(1),]

        VCV_DPI_Trigger_Full_1[c(VCV_row+l),c(4)]<-Control_Batch[l,]

        VCV_DPI_Trigger_Full_1[c(VCV_row+l),c(5)]<-c("VCV_DPI_Trigger_1")
```

```
        VCV_DPI_Trigger_Full_1[c(VCV_row+l),c(6)]<-Value_Trigger[c(a),]
        VCV_DPI_Trigger_Full_1[c(VCV_row+l),c(7)]<-Derivative_Trigger[i,2]

        OUR_DPI_Trigger_Full_1[c(OUR_row+l),c(1:3)]<-Temp_Filter_OUR_Full[c(1),]
        OUR_DPI_Trigger_Full_1[c(OUR_row+l),c(4)]<-Control_Batch[l,]
        OUR_DPI_Trigger_Full_1[c(OUR_row+l),c(5)]<-c("OUR_DPI_Trigger_1")
        OUR_DPI_Trigger_Full_1[c(OUR_row+l),c(6)]<-Value_Trigger[c(a),]
        OUR_DPI_Trigger_Full_1[c(OUR_row+l),c(7)]<-Derivative_Trigger[i,3]
      }
    }
    #Row numbers for storing trigger data
    DO_row<-nrow(DO_DPI_Trigger_Full_1)
    VCV_row<-nrow(VCV_DPI_Trigger_Full_1)
    OUR_row<-nrow(OUR_DPI_Trigger_Full_1)
  }
}

#### Creating average trends for DO, VCV, OUR, and VII ####
#Average DO trends
colnames(DO_x_Data)<-c(paste(unlist(transpose(Control_Batch)),'x',sep="_"))
colnames(DO_y_Data)<-c(paste(unlist(transpose(Control_Batch)),'y',sep="_"))
DO_x_Mean<-rowMeans(DO_x_Data[,-c(1:2)],na.rm=TRUE)
DO_y_Mean<-rowMeans(DO_y_Data[,-c(1:2)],na.rm=TRUE)
DO_x_SD<-apply(DO_x_Data[,-c(1:2)],1,sd,na.rm=TRUE)
DO_y_SD<-apply(DO_y_Data[,-c(1:2)],1,sd,na.rm=TRUE)
```

```
DO_x_Data<-cbind(DO_x_Data,DO_x_Mean,DO_x_SD)

DO_y_Data<-cbind(DO_y_Data,DO_y_Mean,DO_y_SD)


Batch_1_DO<-as.data.frame(DO_x_Data[,c(1)])

Batch_1_DO[,c(2)]<-as.data.frame(DO_y_Data[,c(1)])

colnames(Batch_1_DO)<-c("DO_x_Mean","DO_y_Mean")


Batch_2_DO<-as.data.frame(DO_x_Data[,c(2)])

Batch_2_DO[,c(2)]<-as.data.frame(DO_y_Data[,c(2)])

colnames(Batch_2_DO)<-c("DO_x_Mean","DO_y_Mean")


Temp_DO_1<-difference_left_join(DO_x_Data,Batch_1_DO,by=c("DO_x_Mean"),max_dist=1e-4
)

Temp_DO_2<-difference_left_join(DO_x_Data,Batch_2_DO,by=c("DO_x_Mean"),max_dist=1e-4
)


DO_x_Data<-DO_x_Data[,-c(1:2,18:19)]

DO_y_Data<-DO_y_Data[,-c(1:2,18:19)]


DO_x_Data<-add_column(DO_x_Data,Temp_DO_1[,c(20)],.before="3L-020 A1_x")

DO_x_Data<-add_column(DO_x_Data,Temp_DO_2[,c(20)],.before="3L-020 A1_x")

DO_y_Data<-add_column(DO_y_Data,Temp_DO_1[,c(21)],.before="3L-020 A1_y")

DO_y_Data<-add_column(DO_y_Data,Temp_DO_2[,c(21)],.before="3L-020 A1_y")

colnames(DO_x_Data)<-c(paste(unlist(transpose(Control_Batch)),'x',sep="_"))

colnames(DO_y_Data)<-c(paste(unlist(transpose(Control_Batch)),'y',sep="_"))

DO_x_Mean<-rowMeans(DO_x_Data,na.rm=TRUE)
```

```
DO_y_Mean<-rowMeans(DO_y_Data,na.rm=TRUE)

DO_x_SD<-apply(DO_x_Data,1,sd,na.rm=TRUE)

DO_y_SD<-apply(DO_y_Data,1,sd,na.rm=TRUE)

DO_x_Data<-cbind(DO_x_Data,DO_x_Mean,DO_x_SD)

DO_y_Data<-cbind(DO_y_Data,DO_y_Mean,DO_y_SD)

Mean_Merge_DO_Data<-cbind(DO_x_Data[,c(18:19)],DO_y_Data[,c(18:19)])


#Average VCV trends

colnames(VCV_x_Data)<-c(paste(unlist(transpose(Control_Batch)),'x',sep="_"))

colnames(VCV_y_Data)<-c(paste(unlist(transpose(Control_Batch)),'y',sep="_"))

VCV_x_Mean<-rowMeans(VCV_x_Data[,-c(1:2)],na.rm=TRUE)

VCV_y_Mean<-rowMeans(VCV_y_Data[,-c(1:2)],na.rm=TRUE)

VCV_x_SD<-apply(VCV_x_Data[,-c(1:2)],1,sd,na.rm=TRUE)

VCV_y_SD<-apply(VCV_y_Data[,-c(1:2)],1,sd,na.rm=TRUE)

VCV_x_Data<-cbind(VCV_x_Data,VCV_x_Mean,VCV_x_SD)

VCV_y_Data<-cbind(VCV_y_Data,VCV_y_Mean,VCV_y_SD)


Batch_1_VCV<-as.data.frame(VCV_x_Data[,c(1)])

Batch_1_VCV[,c(2)]<-as.data.frame(VCV_y_Data[,c(1)])

colnames(Batch_1_VCV)<-c("VCV_x_Mean","VCV_y_Mean")


Batch_2_VCV<-as.data.frame(VCV_x_Data[,c(2)])

Batch_2_VCV[,c(2)]<-as.data.frame(VCV_y_Data[,c(2)])

colnames(Batch_2_VCV)<-c("VCV_x_Mean","VCV_y_Mean")
```

```
Temp_VCV_1<-difference_left_join(VCV_x_Data,Batch_1_VCV,by=c("VCV_x_Mean"),max_dist=
1e-4)

Temp_VCV_2<-difference_left_join(VCV_x_Data,Batch_2_VCV,by=c("VCV_x_Mean"),max_dist=
1e-4)


VCV_x_Data<-VCV_x_Data[,-c(1:2,18:19)]

VCV_y_Data<-VCV_y_Data[,-c(1:2,18:19)]


VCV_x_Data<-add_column(VCV_x_Data,Temp_VCV_1[,c(20)],.before="3L-020 A1_x")

VCV_x_Data<-add_column(VCV_x_Data,Temp_VCV_2[,c(20)],.before="3L-020 A1_x")

VCV_y_Data<-add_column(VCV_y_Data,Temp_VCV_1[,c(21)],.before="3L-020 A1_y")

VCV_y_Data<-add_column(VCV_y_Data,Temp_VCV_2[,c(21)],.before="3L-020 A1_y")

colnames(VCV_x_Data)<-c(paste(unlist(transpose(Control_Batch)),'x',sep="_"))

colnames(VCV_y_Data)<-c(paste(unlist(transpose(Control_Batch)),'y',sep="_"))

VCV_x_Mean<-rowMeans(VCV_x_Data,na.rm=TRUE)

VCV_y_Mean<-rowMeans(VCV_y_Data,na.rm=TRUE)

VCV_x_SD<-apply(VCV_x_Data,1,sd,na.rm=TRUE)

VCV_y_SD<-apply(VCV_y_Data,1,sd,na.rm=TRUE)

VCV_x_Data<-cbind(VCV_x_Data,VCV_x_Mean,VCV_x_SD)

VCV_y_Data<-cbind(VCV_y_Data,VCV_y_Mean,VCV_y_SD)

Mean_Merge_VCV_Data<-cbind(VCV_x_Data[,c(18:19)],VCV_y_Data[,c(18:19)])


#Average OUR trends

colnames(OUR_x_Data)<-c(paste(unlist(transpose(Control_Batch)),'x',sep="_"))

colnames(OUR_y_Data)<-c(paste(unlist(transpose(Control_Batch)),'y',sep="_"))

OUR_x_Mean<-rowMeans(OUR_x_Data[,-c(1:2)],na.rm=TRUE)
```

```
OUR_y_Mean<-rowMeans(OUR_y_Data[,-c(1:2)],na.rm=TRUE)

OUR_x_SD<-apply(OUR_x_Data[,-c(1:2)],1,sd,na.rm=TRUE)

OUR_y_SD<-apply(OUR_y_Data[,-c(1:2)],1,sd,na.rm=TRUE)

OUR_x_Data<-cbind(OUR_x_Data,OUR_x_Mean,OUR_x_SD)

OUR_y_Data<-cbind(OUR_y_Data,OUR_y_Mean,OUR_y_SD)


Batch_1_OUR<-as.data.frame(OUR_x_Data[,c(1)])

Batch_1_OUR[,c(2)]<-as.data.frame(OUR_y_Data[,c(1)])

colnames(Batch_1_OUR)<-c("OUR_x_Mean","OUR_y_Mean")


Batch_2_OUR<-as.data.frame(OUR_x_Data[,c(2)])

Batch_2_OUR[,c(2)]<-as.data.frame(OUR_y_Data[,c(2)])

colnames(Batch_2_OUR)<-c("OUR_x_Mean","OUR_y_Mean")


Temp_OUR_1<-difference_left_join(OUR_x_Data,Batch_1_OUR,by=c("OUR_x_Mean"),max_dist=
1e-4)

Temp_OUR_2<-difference_left_join(OUR_x_Data,Batch_2_OUR,by=c("OUR_x_Mean"),max_dist=
1e-4)


OUR_x_Data<-OUR_x_Data[,-c(1:2,18:19)]

OUR_y_Data<-OUR_y_Data[,-c(1:2,18:19)]


OUR_x_Data<-add_column(OUR_x_Data,Temp_OUR_1[,c(20)],.before="3L-020 A1_x")

OUR_x_Data<-add_column(OUR_x_Data,Temp_OUR_2[,c(20)],.before="3L-020 A1_x")

OUR_y_Data<-add_column(OUR_y_Data,Temp_OUR_1[,c(21)],.before="3L-020 A1_y")

OUR_y_Data<-add_column(OUR_y_Data,Temp_OUR_2[,c(21)],.before="3L-020 A1_y")
```

```
colnames(OUR_x_Data)<-c(paste(unlist(transpose(Control_Batch)),'x',sep="_"))

colnames(OUR_y_Data)<-c(paste(unlist(transpose(Control_Batch)),'y',sep="_"))

OUR_x_Mean<-rowMeans(OUR_x_Data,na.rm=TRUE)

OUR_y_Mean<-rowMeans(OUR_y_Data,na.rm=TRUE)

OUR_x_SD<-apply(OUR_x_Data,1,sd,na.rm=TRUE)

OUR_y_SD<-apply(OUR_y_Data,1,sd,na.rm=TRUE)

OUR_x_Data<-cbind(OUR_x_Data,OUR_x_Mean,OUR_x_SD)

OUR_y_Data<-cbind(OUR_y_Data,OUR_y_Mean,OUR_y_SD)

Mean_Merge_OUR_Data<-cbind(OUR_x_Data[,c(18:19)],OUR_y_Data[,c(18:19)])


#Average VII Trends

colnames(VII_x_Data)<-c(paste(unlist(transpose(Control_Batch)),'x',sep="_"))

colnames(VII_y_Data)<-c(paste(unlist(transpose(Control_Batch)),'y',sep="_"))

VII_x_Mean<-rowMeans(VII_x_Data[,-c(1:2)],na.rm=TRUE)

VII_y_Mean<-rowMeans(VII_y_Data[,-c(1:2)],na.rm=TRUE)

VII_x_SD<-apply(VII_x_Data[,-c(1:2)],1,sd,na.rm=TRUE)

VII_y_SD<-apply(VII_y_Data[,-c(1:2)],1,sd,na.rm=TRUE)

VII_x_Data<-cbind(VII_x_Data,VII_x_Mean,VII_x_SD)

VII_y_Data<-cbind(VII_y_Data,VII_y_Mean,VII_y_SD)


Batch_1_VII<-as.data.frame(VII_x_Data[,c(1)])

Batch_1_VII[,c(2)]<-as.data.frame(VII_y_Data[,c(1)])

colnames(Batch_1_VII)<-c("VII_x_Mean","VII_y_Mean")


Batch_2_VII<-as.data.frame(VII_x_Data[,c(2)])
```

```
Batch_2_VII[,c(2)]<-as.data.frame(VII_y_Data[,c(2)])

colnames(Batch_2_VII)<-c("VII_x_Mean","VII_y_Mean")


Temp_VII_1<-difference_left_join(VII_x_Data,Batch_1_VII,by=c("VII_x_Mean"),max_dist=
1e-4)

Temp_VII_2<-difference_left_join(VII_x_Data,Batch_2_VII,by=c("VII_x_Mean"),max_dist=
1e-4)


VII_x_Data<-VII_x_Data[,-c(1:2,18:19)]

VII_y_Data<-VII_y_Data[,-c(1:2,18:19)]


VII_x_Data<-add_column(VII_x_Data,Temp_VII_1[,c(20)],.before="3L-020 A1_x")

VII_x_Data<-add_column(VII_x_Data,Temp_VII_2[,c(20)],.before="3L-020 A1_x")

VII_y_Data<-add_column(VII_y_Data,Temp_VII_1[,c(21)],.before="3L-020 A1_y")

VII_y_Data<-add_column(VII_y_Data,Temp_VII_2[,c(21)],.before="3L-020 A1_y")

colnames(VII_x_Data)<-c(paste(unlist(transpose(Control_Batch)),'x',sep="_"))

colnames(VII_y_Data)<-c(paste(unlist(transpose(Control_Batch)),'y',sep="_"))

VII_x_Mean<-rowMeans(VII_x_Data,na.rm=TRUE)

VII_y_Mean<-rowMeans(VII_y_Data,na.rm=TRUE)

VII_x_SD<-apply(VII_x_Data,1,sd,na.rm=TRUE)

VII_y_SD<-apply(VII_y_Data,1,sd,na.rm=TRUE)

VII_x_Data<-cbind(VII_x_Data,VII_x_Mean,VII_x_SD)

VII_y_Data<-cbind(VII_y_Data,VII_y_Mean,VII_y_SD)

Mean_Merge_VII_Data<-cbind(VII_x_Data[,c(18:19)],VII_y_Data[,c(18:19)])


#### Trigger 2 Calculations ####
```

```
Value_Trigger_2<-data.frame(array(c(30,35,40,45,50,55,60)))

colnames(Value_Trigger_2)<-c("Trigger Value Percent")

DPI_Trigger_2<-1

Derivative_Trigger_2<-matrix(c(20, -5e7, -3e-6,

                             25, -1e7, -5e-6,

                             35, -1e8, -8e-6,

                             45, -1.5e8, -1e-5,

                             50, -8e7, -2e-6),nrow=5,ncol=3,byrow=TRUE)

colnames(Derivative_Trigger_2)<-c("DO_Derivative","VCV_Derivative","OUR_Derivative")



DO_DPI_Trigger_Full_2<-as.data.frame(matrix(ncol=4))

colnames(DO_DPI_Trigger_Full_2)<-c("DO_DPI_Trigger","DO%_Trigger","dDO_Trigger","Bat
ch_ID")



VCV_DPI_Trigger_Full_2<-as.data.frame(matrix(ncol=4))

colnames(VCV_DPI_Trigger_Full_2)<-c("VCV_DPI_Trigger","VCV_Trigger","dVCV_Trigger","
Batch_ID")



OUR_DPI_Trigger_Full_2<-as.data.frame(matrix(ncol=4))

colnames(OUR_DPI_Trigger_Full_2)<-c("OUR_DPI_Trigger","OUR_Trigger","dOUR_Trigger","
Batch_ID")



for (a in 1:nrow(Value_Trigger_2))

{

  for (i in 1:nrow(Derivative_Trigger_2))

  {
```

```
    for (l in 1:Num_Batch_ID)

    {

      h<-filter(OUR_Data_Extended,`Batch
ID`==as.character(Control_Batch[l,])&`Infection?`=="INFECTION")

      f<-filter(OUR_Data_Extended,`Batch ID`==as.character(Control_Batch[l,]))



      #setting up empty matrices and filling them with batch data

      DO_Data_Trim<-as.data.frame(matrix(ncol=3))

      colnames(DO_Data_Trim)<-c("DPI","DO%","dDO%")

      DO_Data_Trim[1:nrow(f),]<-f[,(c("DPI","Avg DO hr (%)","dDO (%)"))]



      VCV_Data_Trim<-as.data.frame(matrix(ncol=3))

      colnames(VCV_Data_Trim)<-c("DPI","VCV","dVCV")

      VCV_Data_Trim[1:nrow(f),]<-f[,(c("DPI","Avg VCV hr (um3 cells)","dVCV (um3
cells)"))]



      OUR_Data_Trim<-as.data.frame(matrix(ncol=3))

      colnames(OUR_Data_Trim)<-c("DPI","OUR","dOUR")

      OUR_Data_Trim[1:nrow(f),]<-f[,(c("DPI","Avg OUR hr (/um3 cells) min","dOUR
(/um3 cells) min" ))]



      #Max and Min DO calculations


Max_DO<-DO_Data_Trim%>%filter(DPI<(-0.5))%>%filter(`DO%`==max(`DO%`,na.rm=TRUE))

      Max_DO<-Max_DO[,c(2)]

      # if (round(Max_DO[,c(1)],0)==(-3))

      # {
```

```
#   Max_DO<-Max_DO[,c(2)]

# }else

# {

#
Max_DO<-DO_Data_Trim%>%filter(DPI>3&`dDO%`<10)%>%filter(`DO%`==max(`DO%`,na.rm=TRUE)
)

#   Max_DO<-Max_DO[,c(2)]

#   if (is_empty(Max_DO))

#   {

#
Max_DO<-DO_Data_Trim%>%filter(DPI>2)%>%filter(`DO%`==max(`DO%`,na.rm=TRUE))

#     Max_DO<-Max_DO[,c(2)]

#   }

# }

Min_DO<-DO_Data_Trim%>%filter(DPI<(-0.1)&`DO%`>0)%>%filter(`DO%`==min(`DO%`,na.rm=TR
UE))

Min_DO<-Min_DO[,c(2)]



#Max and min VCV calculations

Max_VCV<-VCV_Data_Trim%>%filter(DPI<3.5&DPI>0.5)%>%filter(VCV==max(VCV,na.rm=TRUE))

Max_VCV<-Max_VCV[,c(2)]

Min_VCV<-VCV_Data_Trim%>%filter(DPI<(-1))%>%filter(VCV==min(VCV,na.rm=TRUE))

Min_VCV<-Min_VCV[,c(2)]

# if (round(Min_VCV[,c(1)],0)==(-3))

# {

#   Min_VCV<-Min_VCV[,c(2)]
```

```
      # }else
      # {
      #
Min_VCV<-VCV_Data_Trim%>%filter(DPI>2.5&dVCV>(-1e7)|DPI<(-1))%>%filter(VCV==min(VCV,
na.rm=TRUE))
      #   Min_VCV<-Min_VCV[,c(2)]
      #   if (is_empty(Min_VCV))
      #   {
      #     Min_VCV<-VCV_Data_Trim%>%filter()
      #   }
      # }


      #Max and min OUR calculations
      Max_OUR<-OUR_Data_Trim%>%filter(DPI<(-0.5))%>%filter(OUR==max(OUR,na.rm=TRUE))
      Max_OUR<-Max_OUR[,c(2)]
      # if(round(Max_OUR[,c(1)],1)<3&round(Max_OUR[,c(1)],1)>0)
      # {
      #   Max_OUR<-Max_OUR[,c(2)]
      # }else
      # {
      #
Max_OUR<-OUR_Data_Trim%>%filter(DPI>0|DPI<(-0.5))%>%filter(DPI<3&dOUR<1e-8)%>%filter
(OUR==max(OUR,na.rm=TRUE))
      #   Max_OUR<-Max_OUR[,c(2)]
      #   if(is_empty(Max_OUR))
      #   {
      #     Max_OUR<-OUR_Data_Trim%>%filter()
```

```
    #   }
    # }
    Min_OUR<-OUR_Data_Trim%>%filter(DPI<(-1))%>%filter(OUR==min(OUR,na.rm=TRUE))
    Min_OUR<-Min_OUR[,c(2)]
    # if (round(Min_OUR[,c(1)],0)==(-3))
    # {
    #   Min_OUR<-Min_OUR[,c(2)]
    # }else
    # {
    #
Min_OUR<-OUR_Data_Trim%>%filter(DPI>2.5&dOUR>(-1e-8)|DPI<(-1))%>%filter(OUR==min(OUR
,na.rm=TRUE))
    #   Min_OUR<-Min_OUR[,c(2)]
    #   if (is_empty(Min_OUR))
    #   {
    #     Min_OUR<-OUR_Data_Trim%>%filter()
    #   }
    # }

    #Filtering for Trigger

Temp_Filter_DO_Full<-DO_Data_Trim%>%filter(DPI>DPI_Trigger_2)%>%filter(`dDO%`>Deriva
tive_Trigger_2[i,1])%>%filter(`DO%`>Min_DO+(Max_DO-Min_DO)*(Value_Trigger_2[c(a),]/1
00))

Temp_Filter_VCV_Full<-VCV_Data_Trim%>%filter(DPI>DPI_Trigger_2)%>%filter(dVCV<Deriva
tive_Trigger_2[i,2])%>%filter(VCV<Max_VCV-(Max_VCV-Min_VCV)*(Value_Trigger_2[c(a),]/
100))

Temp_Filter_OUR_Full<-OUR_Data_Trim%>%filter(DPI>DPI_Trigger_2)%>%filter(dOUR<Deriva
```

```
tive_Trigger_2[i,3])%>%filter(OUR<Max_OUR-(Max_OUR-Min_OUR)*(Value_Trigger_2[c(a),]/
100))



      #Storing Trigger Data

      if (a==1&i==1)

      {

        DO_DPI_Trigger_Full_2[c(l),c(1:3)]<-Temp_Filter_DO_Full[c(1),]

        DO_DPI_Trigger_Full_2[c(l),c(4)]<-Control_Batch[l,]

DO_DPI_Trigger_Full_2<-DO_DPI_Trigger_Full_2%>%mutate(DO_Label="DO_DPI_Trigger_2",`T
rigger_Value_%`=Value_Trigger_2[c(a),],`Slope_Value`=Derivative_Trigger_2[i,1])

        DO_row<-nrow(DO_DPI_Trigger_Full_2)


        VCV_DPI_Trigger_Full_2[c(l),c(1:3)]<-Temp_Filter_VCV_Full[c(1),]

        VCV_DPI_Trigger_Full_2[c(l),c(4)]<-Control_Batch[l,]

VCV_DPI_Trigger_Full_2<-VCV_DPI_Trigger_Full_2%>%mutate(VCV_Label="VCV_DPI_Trigger_2
",`Trigger_Value_%`=Value_Trigger_2[c(a),],`Slope_Value`=Derivative_Trigger_2[i,2])

        VCV_row<-nrow(VCV_DPI_Trigger_Full_2)


        OUR_DPI_Trigger_Full_2[c(l),c(1:3)]<-Temp_Filter_OUR_Full[c(1),]

        OUR_DPI_Trigger_Full_2[c(l),c(4)]<-Control_Batch[l,]

OUR_DPI_Trigger_Full_2<-OUR_DPI_Trigger_Full_2%>%mutate(OUR_Label="OUR_DPI_Trigger_2
",`Trigger_Value_%`=Value_Trigger_2[c(a),],`Slope_Value`=Derivative_Trigger_2[i,3])

        OUR_row<-nrow(OUR_DPI_Trigger_Full_2)


      }else
```

```
      {
        DO_DPI_Trigger_Full_2[c(DO_row+l),c(1:3)]<-Temp_Filter_DO_Full[c(1),]
        DO_DPI_Trigger_Full_2[c(DO_row+l),c(4)]<-Control_Batch[l,]
        DO_DPI_Trigger_Full_2[c(DO_row+l),c(5)]<-c("DO_DPI_Trigger_2")
        DO_DPI_Trigger_Full_2[c(DO_row+l),c(6)]<-Value_Trigger_2[c(a),]
        DO_DPI_Trigger_Full_2[c(DO_row+l),c(7)]<-Derivative_Trigger_2[i,1]

        VCV_DPI_Trigger_Full_2[c(VCV_row+l),c(1:3)]<-Temp_Filter_VCV_Full[c(1),]
        VCV_DPI_Trigger_Full_2[c(VCV_row+l),c(4)]<-Control_Batch[l,]
        VCV_DPI_Trigger_Full_2[c(VCV_row+l),c(5)]<-c("VCV_DPI_Trigger_2")
        VCV_DPI_Trigger_Full_2[c(VCV_row+l),c(6)]<-Value_Trigger_2[c(a),]
        VCV_DPI_Trigger_Full_2[c(VCV_row+l),c(7)]<-Derivative_Trigger_2[i,2]

        OUR_DPI_Trigger_Full_2[c(OUR_row+l),c(1:3)]<-Temp_Filter_OUR_Full[c(1),]
        OUR_DPI_Trigger_Full_2[c(OUR_row+l),c(4)]<-Control_Batch[l,]
        OUR_DPI_Trigger_Full_2[c(OUR_row+l),c(5)]<-c("OUR_DPI_Trigger_2")
        OUR_DPI_Trigger_Full_2[c(OUR_row+l),c(6)]<-Value_Trigger_2[c(a),]
        OUR_DPI_Trigger_Full_2[c(OUR_row+l),c(7)]<-Derivative_Trigger_2[i,3]
      }
    }
    #Row numbers for storing trigger data
    DO_row<-nrow(DO_DPI_Trigger_Full_2)
    VCV_row<-nrow(VCV_DPI_Trigger_Full_2)
    OUR_row<-nrow(OUR_DPI_Trigger_Full_2)
  }
```

```
}

#### Trigger 3 Calculations ####

Value_Trigger_3<-data.frame(array(c(10,15,20,25,30,35)))

colnames(Value_Trigger_3)<-c("Trigger Value Percent")

DPI_Trigger_3<-1.5

Derivative_Trigger_3<-matrix(c(10, -5e7, -5e-7,

                               15, -1e7, -2e-6,

                               30, -2e8, -1e-6,

                               35, -1.5e8, -4e-7,

                               40, -8e7, -8e-7),nrow=5,ncol=3,byrow=TRUE)

colnames(Derivative_Trigger_3)<-c("DO_Derivative","VCV_Derivative","OUR_Derivative")


DO_DPI_Trigger_Full_3<-as.data.frame(matrix(ncol=4))

colnames(DO_DPI_Trigger_Full_3)<-c("DO_DPI_Trigger","DO%_Trigger","dDO_Trigger","Bat
ch_ID")


VCV_DPI_Trigger_Full_3<-as.data.frame(matrix(ncol=4))

colnames(VCV_DPI_Trigger_Full_3)<-c("VCV_DPI_Trigger","VCV_Trigger","dVCV_Trigger","
Batch_ID")


OUR_DPI_Trigger_Full_3<-as.data.frame(matrix(ncol=4))

colnames(OUR_DPI_Trigger_Full_3)<-c("OUR_DPI_Trigger","OUR_Trigger","dOUR_Trigger","
Batch_ID")


for (a in 1:nrow(Value_Trigger_3))
```

```
{
  for (i in 1:nrow(Derivative_Trigger_3))
  {
    for (l in 1:Num_Batch_ID)
    {
      h<-filter(OUR_Data_Extended,`Batch
ID`==as.character(Control_Batch[l,])&`Infection?`=="INFECTION")
      f<-filter(OUR_Data_Extended,`Batch ID`==as.character(Control_Batch[l,]))


      #setting up empty matrices and filling them with batch data
      DO_Data_Trim<-as.data.frame(matrix(ncol=3))
      colnames(DO_Data_Trim)<-c("DPI","DO%","dDO%")
      DO_Data_Trim[1:nrow(f),]<-f[,(c("DPI","Avg DO hr (%)","dDO (%)"))]


      VCV_Data_Trim<-as.data.frame(matrix(ncol=3))
      colnames(VCV_Data_Trim)<-c("DPI","VCV","dVCV")
      VCV_Data_Trim[1:nrow(f),]<-f[,(c("DPI","Avg VCV hr (um3 cells)","dVCV (um3
cells)"))]


      OUR_Data_Trim<-as.data.frame(matrix(ncol=3))
      colnames(OUR_Data_Trim)<-c("DPI","OUR","dOUR")
      OUR_Data_Trim[1:nrow(f),]<-f[,(c("DPI","Avg OUR hr (/um3 cells) min","dOUR
(/um3 cells) min" ))]


      #Max and Min DO calculations

Max_DO<-DO_Data_Trim%>%filter(DPI<(-0.5))%>%filter(`DO%`==max(`DO%`,na.rm=TRUE))
```

```
      Max_DO<-Max_DO[,c(2)]

      # if (round(Max_DO[,c(1)],0)==(-3))

      # {

      #   Max_DO<-Max_DO[,c(2)]

      # }else

      # {

      #
Max_DO<-DO_Data_Trim%>%filter(DPI>3&`dDO%`<10)%>%filter(`DO%`==max(`DO%`,na.rm=TRUE)
)

      #   Max_DO<-Max_DO[,c(2)]

      #   if (is_empty(Max_DO))

      #   {

      #
Max_DO<-DO_Data_Trim%>%filter(DPI>2)%>%filter(`DO%`==max(`DO%`,na.rm=TRUE))

      #     Max_DO<-Max_DO[,c(2)]

      #   }

      # }


Min_DO<-DO_Data_Trim%>%filter(DPI<(-0.1)&`DO%`>0)%>%filter(`DO%`==min(`DO%`,na.rm=TR
UE))

      Min_DO<-Min_DO[,c(2)]



      #Max and min VCV calculations


Max_VCV<-VCV_Data_Trim%>%filter(DPI<3.5&DPI>0.5)%>%filter(VCV==max(VCV,na.rm=TRUE))

      Max_VCV<-Max_VCV[,c(2)]

      Min_VCV<-VCV_Data_Trim%>%filter(DPI<(-1))%>%filter(VCV==min(VCV,na.rm=TRUE))

      Min_VCV<-Min_VCV[,c(2)]
```

```
# if (round(Min_VCV[,c(1)],0)==(-3))

# {

#   Min_VCV<-Min_VCV[,c(2)]

# }else

# {

#
Min_VCV<-VCV_Data_Trim%>%filter(DPI>2.5&dVCV>(-1e7)|DPI<(-1))%>%filter(VCV==min(VCV,
na.rm=TRUE))

#   Min_VCV<-Min_VCV[,c(2)]

#   if (is_empty(Min_VCV))

#   {

#     Min_VCV<-VCV_Data_Trim%>%filter()

#   }

# }


#Max and min OUR calculations

Max_OUR<-OUR_Data_Trim%>%filter(DPI<(-0.5))%>%filter(OUR==max(OUR,na.rm=TRUE))

Max_OUR<-Max_OUR[,c(2)]

# if(round(Max_OUR[,c(1)],1)<3&round(Max_OUR[,c(1)],1)>0)

# {

#   Max_OUR<-Max_OUR[,c(2)]

# }else

# {

#
Max_OUR<-OUR_Data_Trim%>%filter(DPI>0|DPI<(-0.5))%>%filter(DPI<3&dOUR<1e-8)%>%filter
(OUR==max(OUR,na.rm=TRUE))

#   Max_OUR<-Max_OUR[,c(2)]
```

```
    #   if(is_empty(Max_OUR))
    #   {
    #     Max_OUR<-OUR_Data_Trim%>%filter()
    #   }
    # }
    Min_OUR<-OUR_Data_Trim%>%filter(DPI<(-1))%>%filter(OUR==min(OUR,na.rm=TRUE))
    Min_OUR<-Min_OUR[,c(2)]
    # if (round(Min_OUR[,c(1)],0)==(-3))
    # {
    #   Min_OUR<-Min_OUR[,c(2)]
    # }else
    # {
    #
Min_OUR<-OUR_Data_Trim%>%filter(DPI>2.5&dOUR>(-1e-8)|DPI<(-1))%>%filter(OUR==min(OUR
,na.rm=TRUE))
    #   Min_OUR<-Min_OUR[,c(2)]
    #   if (is_empty(Min_OUR))
    #   {
    #     Min_OUR<-OUR_Data_Trim%>%filter()
    #   }
    # }


    #Filtering for Trigger

Temp_Filter_DO_Full<-DO_Data_Trim%>%filter(DPI>DPI_Trigger_3)%>%filter(`dDO%`<Deriva
tive_Trigger_3[i,1])%>%filter(`DO%`>Max_DO*(1-(Value_Trigger_3[c(a),]/100)))

Temp_Filter_VCV_Full<-VCV_Data_Trim%>%filter(DPI>DPI_Trigger_3)%>%filter(dVCV>Deriva
```

```
tive_Trigger_3[i,2])%>%filter(VCV<Min_VCV+(Max_VCV-Min_VCV)*(Value_Trigger_3[c(a),]/
100))

Temp_Filter_OUR_Full<-OUR_Data_Trim%>%filter(DPI>DPI_Trigger_3)%>%filter(dOUR>Deriva
tive_Trigger_3[i,3])%>%filter(OUR<Min_OUR+(Max_OUR-Min_OUR)*(Value_Trigger_3[c(a),]/
100))



      #Storing Trigger Data

      if (a==1&i==1)

      {

        DO_DPI_Trigger_Full_3[c(l),c(1:3)]<-Temp_Filter_DO_Full[c(1),]

        DO_DPI_Trigger_Full_3[c(l),c(4)]<-Control_Batch[l,]

DO_DPI_Trigger_Full_3<-DO_DPI_Trigger_Full_3%>%mutate(DO_Label="DO_DPI_Trigger_3",`T
rigger_Value_%`=Value_Trigger_3[c(a),],`Slope_Value`=Derivative_Trigger_3[i,1])

        DO_row<-nrow(DO_DPI_Trigger_Full_3)


        VCV_DPI_Trigger_Full_3[c(l),c(1:3)]<-Temp_Filter_VCV_Full[c(1),]

        VCV_DPI_Trigger_Full_3[c(l),c(4)]<-Control_Batch[l,]

VCV_DPI_Trigger_Full_3<-VCV_DPI_Trigger_Full_3%>%mutate(VCV_Label="VCV_DPI_Trigger_3
",`Trigger_Value_%`=Value_Trigger_3[c(a),],`Slope_Value`=Derivative_Trigger_3[i,2])

        VCV_row<-nrow(VCV_DPI_Trigger_Full_3)


        OUR_DPI_Trigger_Full_3[c(l),c(1:3)]<-Temp_Filter_OUR_Full[c(1),]

        OUR_DPI_Trigger_Full_3[c(l),c(4)]<-Control_Batch[l,]

OUR_DPI_Trigger_Full_3<-OUR_DPI_Trigger_Full_3%>%mutate(OUR_Label="OUR_DPI_Trigger_3
",`Trigger_Value_%`=Value_Trigger_3[c(a),],`Slope_Value`=Derivative_Trigger_3[i,3])
```

```
    OUR_row<-nrow(OUR_DPI_Trigger_Full_3)


  }else
  {
    DO_DPI_Trigger_Full_3[c(DO_row+l),c(1:3)]<-Temp_Filter_DO_Full[c(1),]
    DO_DPI_Trigger_Full_3[c(DO_row+l),c(4)]<-Control_Batch[l,]
    DO_DPI_Trigger_Full_3[c(DO_row+l),c(5)]<-c("DO_DPI_Trigger_3")
    DO_DPI_Trigger_Full_3[c(DO_row+l),c(6)]<-Value_Trigger_3[c(a),]
    DO_DPI_Trigger_Full_3[c(DO_row+l),c(7)]<-Derivative_Trigger_3[i,1]

    VCV_DPI_Trigger_Full_3[c(VCV_row+l),c(1:3)]<-Temp_Filter_VCV_Full[c(1),]
    VCV_DPI_Trigger_Full_3[c(VCV_row+l),c(4)]<-Control_Batch[l,]
    VCV_DPI_Trigger_Full_3[c(VCV_row+l),c(5)]<-c("VCV_DPI_Trigger_3")
    VCV_DPI_Trigger_Full_3[c(VCV_row+l),c(6)]<-Value_Trigger_3[c(a),]
    VCV_DPI_Trigger_Full_3[c(VCV_row+l),c(7)]<-Derivative_Trigger_3[i,2]

    OUR_DPI_Trigger_Full_3[c(OUR_row+l),c(1:3)]<-Temp_Filter_OUR_Full[c(1),]
    OUR_DPI_Trigger_Full_3[c(OUR_row+l),c(4)]<-Control_Batch[l,]
    OUR_DPI_Trigger_Full_3[c(OUR_row+l),c(5)]<-c("OUR_DPI_Trigger_3")
    OUR_DPI_Trigger_Full_3[c(OUR_row+l),c(6)]<-Value_Trigger_3[c(a),]
    OUR_DPI_Trigger_Full_3[c(OUR_row+l),c(7)]<-Derivative_Trigger_3[i,3]
  }
}
#Row numbers for storing trigger data
DO_row<-nrow(DO_DPI_Trigger_Full_3)
```

```
    VCV_row<-nrow(VCV_DPI_Trigger_Full_3)

    OUR_row<-nrow(OUR_DPI_Trigger_Full_3)

  }

}



DO_DPI_Trigger_Full_1<-merge(DO_DPI_Trigger_Full_1,Peak_VII[,c("Peak_Cont_VII_DPI","Peak_Cont_VII","Batch_ID")],by.x="Batch_ID")%>%mutate(DPI_Offset=Peak_Cont_VII_DPI-DO_DPI_Trigger)

DO_DPI_Trigger_Full_2<-merge(DO_DPI_Trigger_Full_2,Peak_VII[,c("Peak_Cont_VII_DPI","Peak_Cont_VII","Batch_ID")],by.x="Batch_ID")%>%mutate(DPI_Offset=Peak_Cont_VII_DPI-DO_DPI_Trigger)

DO_DPI_Trigger_Full_3<-merge(DO_DPI_Trigger_Full_3,Peak_VII[,c("Peak_Cont_VII_DPI","Peak_Cont_VII","Batch_ID")],by.x="Batch_ID")%>%mutate(DPI_Offset=Peak_Cont_VII_DPI-DO_DPI_Trigger)



VCV_DPI_Trigger_Full_1<-merge(VCV_DPI_Trigger_Full_1,Peak_VII[,c("Peak_Cont_VII_DPI","Peak_Cont_VII","Batch_ID")],by.x="Batch_ID")%>%mutate(DPI_Offset=Peak_Cont_VII_DPI-VCV_DPI_Trigger)

VCV_DPI_Trigger_Full_2<-merge(VCV_DPI_Trigger_Full_2,Peak_VII[,c("Peak_Cont_VII_DPI","Peak_Cont_VII","Batch_ID")],by.x="Batch_ID")%>%mutate(DPI_Offset=Peak_Cont_VII_DPI-VCV_DPI_Trigger)

VCV_DPI_Trigger_Full_3<-merge(VCV_DPI_Trigger_Full_3,Peak_VII[,c("Peak_Cont_VII_DPI","Peak_Cont_VII","Batch_ID")],by.x="Batch_ID")%>%mutate(DPI_Offset=Peak_Cont_VII_DPI-VCV_DPI_Trigger)



OUR_DPI_Trigger_Full_1<-merge(OUR_DPI_Trigger_Full_1,Peak_VII[,c("Peak_Cont_VII_DPI","Peak_Cont_VII","Batch_ID")],by.x="Batch_ID")%>%mutate(DPI_Offset=Peak_Cont_VII_DPI-OUR_DPI_Trigger)

OUR_DPI_Trigger_Full_2<-merge(OUR_DPI_Trigger_Full_2,Peak_VII[,c("Peak_Cont_VII_DPI","Peak_Cont_VII","Batch_ID")],by.x="Batch_ID")%>%mutate(DPI_Offset=Peak_Cont_VII_DPI-OUR_DPI_Trigger)

OUR_DPI_Trigger_Full_3<-merge(OUR_DPI_Trigger_Full_3,Peak_VII[,c("Peak_Cont_VII_DPI","Peak_Cont_VII","Batch_ID")],by.x="Batch_ID")%>%mutate(DPI_Offset=Peak_Cont_VII_DPI-OUR_DPI_Trigger)
```

```
#### Further Analyses ####

DO_Trigger_Summary_1<-group_by(DO_DPI_Trigger_Full_1,`Trigger_Value_%`,`Slope_Value`
)%>%summarize(Mean_Trigger_DPI=mean(DO_DPI_Trigger,na.rm=TRUE),SD_DPI_Trigger=sd(DO_
DPI_Trigger,na.rm=TRUE),Mean_DPI_Offset=mean(DPI_Offset,na.rm=TRUE),SD_DPI_Offset=sd
(DPI_Offset,na.rm=TRUE))%>%mutate(Mean_Peak_VII_DPI=mean(Peak_VII[,c(1)]))

DO_Trigger_Summary_2<-group_by(DO_DPI_Trigger_Full_2,`Trigger_Value_%`,`Slope_Value`
)%>%summarize(Mean_Trigger_DPI=mean(DO_DPI_Trigger,na.rm=TRUE),SD_DPI_Trigger=sd(DO_
DPI_Trigger,na.rm=TRUE),Mean_DPI_Offset=mean(DPI_Offset,na.rm=TRUE),SD_DPI_Offset=sd
(DPI_Offset,na.rm=TRUE))%>%mutate(Mean_Peak_VII_DPI=mean(Peak_VII[,c(1)]))

DO_Trigger_Summary_3<-group_by(DO_DPI_Trigger_Full_3,`Trigger_Value_%`,`Slope_Value`
)%>%summarize(Mean_Trigger_DPI=mean(DO_DPI_Trigger,na.rm=TRUE),SD_DPI_Trigger=sd(DO_
DPI_Trigger,na.rm=TRUE),Mean_DPI_Offset=mean(DPI_Offset,na.rm=TRUE),SD_DPI_Offset=sd
(DPI_Offset,na.rm=TRUE))%>%mutate(Mean_Peak_VII_DPI=mean(Peak_VII[,c(1)]))



VCV_Trigger_Summary_1<-group_by(VCV_DPI_Trigger_Full_1,`Trigger_Value_%`,`Slope_Valu
e`)%>%summarize(Mean_Trigger_DPI=mean(VCV_DPI_Trigger,na.rm=TRUE),SD_DPI_Trigger=sd(
VCV_DPI_Trigger,na.rm=TRUE),Mean_DPI_Offset=mean(DPI_Offset,na.rm=TRUE),SD_DPI_Offse
t=sd(DPI_Offset,na.rm=TRUE))%>%mutate(Mean_Peak_VII_DPI=mean(Peak_VII[,c(1)]))

VCV_Trigger_Summary_2<-group_by(VCV_DPI_Trigger_Full_2,`Trigger_Value_%`,`Slope_Valu
e`)%>%summarize(Mean_Trigger_DPI=mean(VCV_DPI_Trigger,na.rm=TRUE),SD_DPI_Trigger=sd(
VCV_DPI_Trigger,na.rm=TRUE),Mean_DPI_Offset=mean(DPI_Offset,na.rm=TRUE),SD_DPI_Offse
t=sd(DPI_Offset,na.rm=TRUE))%>%mutate(Mean_Peak_VII_DPI=mean(Peak_VII[,c(1)]))

VCV_Trigger_Summary_3<-group_by(VCV_DPI_Trigger_Full_3,`Trigger_Value_%`,`Slope_Valu
e`)%>%summarize(Mean_Trigger_DPI=mean(VCV_DPI_Trigger,na.rm=TRUE),SD_DPI_Trigger=sd(
VCV_DPI_Trigger,na.rm=TRUE),Mean_DPI_Offset=mean(DPI_Offset,na.rm=TRUE),SD_DPI_Offse
t=sd(DPI_Offset,na.rm=TRUE))%>%mutate(Mean_Peak_VII_DPI=mean(Peak_VII[,c(1)]))



OUR_Trigger_Summary_1<-group_by(OUR_DPI_Trigger_Full_1,`Trigger_Value_%`,`Slope_Valu
e`)%>%summarize(Mean_Trigger_DPI=mean(OUR_DPI_Trigger,na.rm=TRUE),SD_DPI_Trigger=sd(
OUR_DPI_Trigger,na.rm=TRUE),Mean_DPI_Offset=mean(DPI_Offset,na.rm=TRUE),SD_DPI_Offse
t=sd(DPI_Offset,na.rm=TRUE))%>%mutate(Mean_Peak_VII_DPI=mean(Peak_VII[,c(1)]))

OUR_Trigger_Summary_2<-group_by(OUR_DPI_Trigger_Full_2,`Trigger_Value_%`,`Slope_Valu
e`)%>%summarize(Mean_Trigger_DPI=mean(OUR_DPI_Trigger,na.rm=TRUE),SD_DPI_Trigger=sd(
OUR_DPI_Trigger,na.rm=TRUE),Mean_DPI_Offset=mean(DPI_Offset,na.rm=TRUE),SD_DPI_Offse
t=sd(DPI_Offset,na.rm=TRUE))%>%mutate(Mean_Peak_VII_DPI=mean(Peak_VII[,c(1)]))
```

```
OUR_Trigger_Summary_3<-group_by(OUR_DPI_Trigger_Full_3,`Trigger_Value_%`,`Slope_Valu
e`)%>%summarize(Mean_Trigger_DPI=mean(OUR_DPI_Trigger,na.rm=TRUE),SD_DPI_Trigger=sd(
OUR_DPI_Trigger,na.rm=TRUE),Mean_DPI_Offset=mean(DPI_Offset,na.rm=TRUE),SD_DPI_Offse
t=sd(DPI_Offset,na.rm=TRUE))%>%mutate(Mean_Peak_VII_DPI=mean(Peak_VII[,c(1)]))


DO_DPI_Trigger_Full_1<-merge(DO_DPI_Trigger_Full_1,DO_Trigger_Summary_1,by.x=c("Trig
ger_Value_%","Slope_Value"))

DO_DPI_Trigger_Full_2<-merge(DO_DPI_Trigger_Full_2,DO_Trigger_Summary_2,by.x=c("Trig
ger_Value_%","Slope_Value"))

DO_DPI_Trigger_Full_3<-merge(DO_DPI_Trigger_Full_3,DO_Trigger_Summary_3,by.x=c("Trig
ger_Value_%","Slope_Value"))


VCV_DPI_Trigger_Full_1<-merge(VCV_DPI_Trigger_Full_1,VCV_Trigger_Summary_1,by.x=c("T
rigger_Value_%","Slope_Value"))

VCV_DPI_Trigger_Full_2<-merge(VCV_DPI_Trigger_Full_2,VCV_Trigger_Summary_2,by.x=c("T
rigger_Value_%","Slope_Value"))

VCV_DPI_Trigger_Full_3<-merge(VCV_DPI_Trigger_Full_3,VCV_Trigger_Summary_3,by.x=c("T
rigger_Value_%","Slope_Value"))


OUR_DPI_Trigger_Full_1<-merge(OUR_DPI_Trigger_Full_1,OUR_Trigger_Summary_1,by.x=c("T
rigger_Value_%","Slope_Value"))

OUR_DPI_Trigger_Full_2<-merge(OUR_DPI_Trigger_Full_2,OUR_Trigger_Summary_2,by.x=c("T
rigger_Value_%","Slope_Value"))

OUR_DPI_Trigger_Full_3<-merge(OUR_DPI_Trigger_Full_3,OUR_Trigger_Summary_3,by.x=c("T
rigger_Value_%","Slope_Value"))


DO_DPI_Trigger_Total<-rbind(DO_DPI_Trigger_Full_1,DO_DPI_Trigger_Full_2,DO_DPI_Trigg
er_Full_3)%>%mutate(Predicted_Peak_VII_DPI=DO_DPI_Trigger+Mean_DPI_Offset)

VCV_DPI_Trigger_Total<-rbind(VCV_DPI_Trigger_Full_1,VCV_DPI_Trigger_Full_2,VCV_DPI_T
rigger_Full_3)%>%mutate(Predicted_Peak_VII_DPI=VCV_DPI_Trigger+Mean_DPI_Offset)

OUR_DPI_Trigger_Total<-rbind(OUR_DPI_Trigger_Full_1,OUR_DPI_Trigger_Full_2,OUR_DPI_T
rigger_Full_3)%>%mutate(Predicted_Peak_VII_DPI=OUR_DPI_Trigger+Mean_DPI_Offset)
```

```
DO_Trigger_Count<-data.frame(matrix(nrow=nrow(DO_DPI_Trigger_Total),ncol=9))

VCV_Trigger_Count<-data.frame(matrix(nrow=nrow(VCV_DPI_Trigger_Total),ncol=9))

OUR_Trigger_Count<-data.frame(matrix(nrow=nrow(OUR_DPI_Trigger_Total),ncol=9))



for (n in 1:nrow(DO_Trigger_Count))

{


Temp_Peak_VII<-as.matrix(Peak_VII%>%filter(Batch_ID==DO_DPI_Trigger_Total[c(n),c("Ba
tch_ID")]))

  for (i in 1:nrow(VII_Percentiles))

  {


Percentile_Range<-as.numeric(Temp_Peak_VII[grep(paste(VII_Percentiles[c(i),],"th","
DPI",sep=""),colnames(Temp_Peak_VII))])

    if (is.na(DO_DPI_Trigger_Total[c(n),c("Predicted_Peak_VII_DPI")]))

    {

      DO_Trigger_Count[c(n),c(i)]<-0

      DO_Trigger_Count[c(n),c(8)]<-NA

      DO_Trigger_Count[c(n),c(9)]<-NA

    }else

    {

      if
(DO_DPI_Trigger_Total[c(n),c("Predicted_Peak_VII_DPI")]>Percentile_Range[1]&&DO_DPI_
Trigger_Total[c(n),c("Predicted_Peak_VII_DPI")]<Percentile_Range[2])

      {

        DO_Trigger_Count[c(n),c(i)]<-1

        DO_Trigger_Count[c(n),c(8)]<-OUR_Data_Extended%>%filter(`Batch
ID`==DO_DPI_Trigger_Total[c(n),c("Batch_ID")]&DPI<DO_DPI_Trigger_Total[c(n),c("Predi
cted_Peak_VII_DPI")])%>%select(Step_100_row_VII)%>%tail(.,n=1)
```

```
        DO_Trigger_Count[c(n),c(9)]<-as.numeric(Temp_Peak_VII[2])

      }else

      {

        DO_Trigger_Count[c(n),c(i)]<-0

        DO_Trigger_Count[c(n),c(8)]<-OUR_Data_Extended%>%filter(`Batch 
ID`==DO_DPI_Trigger_Total[c(n),c("Batch_ID")]&DPI<DO_DPI_Trigger_Total[c(n),c("Predi
cted_Peak_VII_DPI")])%>%select(Step_100_row_VII)%>%tail(.,n=1)

        DO_Trigger_Count[c(n),c(9)]<-as.numeric(Temp_Peak_VII[2])

      }

    }

  }

}



for (n in 1:nrow(VCV_Trigger_Count))

{


Temp_Peak_VII<-as.matrix(Peak_VII%>%filter(Batch_ID==VCV_DPI_Trigger_Total[c(n),c("B
atch_ID")]))

  for (i in 1:nrow(VII_Percentiles))

  {


Percentile_Range<-as.numeric(Temp_Peak_VII[grep(paste(VII_Percentiles[c(i),],"th","
DPI",sep=""),colnames(Temp_Peak_VII))])

    if (is.na(VCV_DPI_Trigger_Total[c(n),c("Predicted_Peak_VII_DPI")]))

    {

      VCV_Trigger_Count[c(n),c(i)]<-0

      VCV_Trigger_Count[c(n),c(8)]<-NA

      VCV_Trigger_Count[c(n),c(9)]<-NA
```

```
    }else

    {

      if
(VCV_DPI_Trigger_Total[c(n),c("Predicted_Peak_VII_DPI")]>Percentile_Range[1]&&VCV_DP
I_Trigger_Total[c(n),c("Predicted_Peak_VII_DPI")]<Percentile_Range[2])

      {

        VCV_Trigger_Count[c(n),c(i)]<-1

        VCV_Trigger_Count[c(n),c(8)]<-OUR_Data_Extended%>%filter(`Batch
ID`==VCV_DPI_Trigger_Total[c(n),c("Batch_ID")]&DPI<VCV_DPI_Trigger_Total[c(n),c("Pre
dicted_Peak_VII_DPI")])%>%select(Step_100_row_VII)%>%tail(.,n=1)

        VCV_Trigger_Count[c(n),c(9)]<-as.numeric(Temp_Peak_VII[2])

      }else

      {

        VCV_Trigger_Count[c(n),c(i)]<-0

        VCV_Trigger_Count[c(n),c(8)]<-OUR_Data_Extended%>%filter(`Batch
ID`==VCV_DPI_Trigger_Total[c(n),c("Batch_ID")]&DPI<VCV_DPI_Trigger_Total[c(n),c("Pre
dicted_Peak_VII_DPI")])%>%select(Step_100_row_VII)%>%tail(.,n=1)

        VCV_Trigger_Count[c(n),c(9)]<-as.numeric(Temp_Peak_VII[2])

      }

    }

  }

}



for (n in 1:nrow(OUR_Trigger_Count))

{


Temp_Peak_VII<-as.matrix(Peak_VII%>%filter(Batch_ID==OUR_DPI_Trigger_Total[c(n),c("B
atch_ID")]))

  for (i in 1:nrow(VII_Percentiles))
```

```
  {

Percentile_Range<-as.numeric(Temp_Peak_VII[grep(paste(VII_Percentiles[c(i),],"th","
DPI",sep=""),colnames(Temp_Peak_VII))])

    if (is.na(OUR_DPI_Trigger_Total[c(n),c("Predicted_Peak_VII_DPI")]))

    {

      OUR_Trigger_Count[c(n),c(i)]<-0

      OUR_Trigger_Count[c(n),c(8)]<-NA

      OUR_Trigger_Count[c(n),c(9)]<-NA

    }else

    {

      if
(OUR_DPI_Trigger_Total[c(n),c("Predicted_Peak_VII_DPI")]>Percentile_Range[1]&&OUR_DP
I_Trigger_Total[c(n),c("Predicted_Peak_VII_DPI")]<Percentile_Range[2])

      {

        OUR_Trigger_Count[c(n),c(i)]<-1

        OUR_Trigger_Count[c(n),c(8)]<-OUR_Data_Extended%>%filter(`Batch
ID`==OUR_DPI_Trigger_Total[c(n),c("Batch_ID")]&DPI<OUR_DPI_Trigger_Total[c(n),c("Pre
dicted_Peak_VII_DPI")])%>%select(Step_100_row_VII)%>%tail(.,n=1)

        OUR_Trigger_Count[c(n),c(9)]<-as.numeric(Temp_Peak_VII[2])

      }else

      {

        OUR_Trigger_Count[c(n),c(i)]<-0

        OUR_Trigger_Count[c(n),c(8)]<-OUR_Data_Extended%>%filter(`Batch
ID`==OUR_DPI_Trigger_Total[c(n),c("Batch_ID")]&DPI<OUR_DPI_Trigger_Total[c(n),c("Pre
dicted_Peak_VII_DPI")])%>%select(Step_100_row_VII)%>%tail(.,n=1)

        OUR_Trigger_Count[c(n),c(9)]<-as.numeric(Temp_Peak_VII[2])

      }

    }
```

```
  }

}


colnames(DO_Trigger_Count)<-c(t(VII_Percentiles),"Predicted_Peak_VII","Peak_Cont_VII")

colnames(VCV_Trigger_Count)<-c(t(VII_Percentiles),"Predicted_Peak_VII","Peak_Cont_VII")

colnames(OUR_Trigger_Count)<-c(t(VII_Percentiles),"Predicted_Peak_VII","Peak_Cont_VII")


DO_Trigger_Count<-DO_Trigger_Count%>%mutate(Percent_Peak_Cont_VII=(Predicted_Peak_VII/Peak_Cont_VII)*100)

VCV_Trigger_Count<-VCV_Trigger_Count%>%mutate(Percent_Peak_Cont_VII=(Predicted_Peak_VII/Peak_Cont_VII)*100)

OUR_Trigger_Count<-OUR_Trigger_Count%>%mutate(Percent_Peak_Cont_VII=(Predicted_Peak_VII/Peak_Cont_VII)*100)


DO_Trigger_Count_Summary<-cbind(DO_DPI_Trigger_Total[,c(1,2,7)],DO_Trigger_Count)%>%

mutate(Identifier=paste(paste(`Trigger_Value_%`,"%",sep=""),`Slope_Value`,DO_Label,sep="_"))%>%

  group_by(Identifier)%>%

summarize(`95_Sum`=sum(`95`,na.rm=TRUE),`90_Sum`=sum(`90`,na.rm=TRUE),`85_Sum`=sum(`85`,na.rm=TRUE),`80_Sum`=sum(`80`,na.rm=TRUE),`75_Sum`=sum(`75`,na.rm=TRUE),`70_Sum`=sum(`70`,na.rm=TRUE),`65_Sum`=sum(`65`,na.rm=TRUE),`Average_%_Peak_VII`=mean(Percent_Peak_Cont_VII,na.rm=TRUE),`SD_%_Peak_VII`=sd(Percent_Peak_Cont_VII,na.rm=TRUE))%>%

mutate(`95%`=(`95_Sum`/nrow(Control_Batch))*100,`90%`=(`90_Sum`/nrow(Control_Batch))*100,`85%`=(`85_Sum`/nrow(Control_Batch))*100,`80%`=(`80_Sum`/nrow(Control_Batch))*100,`75%`=(`75_Sum`/nrow(Control_Batch))*100,`70%`=(`70_Sum`/nrow(Control_Batch))*100,`65%`=(`65_Sum`/nrow(Control_Batch))*100)
```

```
VCV_Trigger_Count_Summary<-cbind(VCV_DPI_Trigger_Total[,c(1,2,7)],VCV_Trigger_Count)
%>%

mutate(Identifier=paste(paste(`Trigger_Value_%`,"%",sep=""),`Slope_Value`,VCV_Label,
sep="_"))%>%

  group_by(Identifier)%>%

summarize(`95_Sum`=sum(`95`,na.rm=TRUE),`90_Sum`=sum(`90`,na.rm=TRUE),`85_Sum`=sum(`
85`,na.rm=TRUE),`80_Sum`=sum(`80`,na.rm=TRUE),`75_Sum`=sum(`75`,na.rm=TRUE),`70_Sum`
=sum(`70`,na.rm=TRUE),`65_Sum`=sum(`65`,na.rm=TRUE),`Average_%_Peak_VII`=mean(Percen
t_Peak_Cont_VII,na.rm=TRUE),`SD_%_Peak_VII`=sd(Percent_Peak_Cont_VII,na.rm=TRUE))%>%

mutate(`95%`=(`95_Sum`/nrow(Control_Batch))*100,`90%`=(`90_Sum`/nrow(Control_Batch))
*100,`85%`=(`85_Sum`/nrow(Control_Batch))*100,`80%`=(`80_Sum`/nrow(Control_Batch))*1
00,`75%`=(`75_Sum`/nrow(Control_Batch))*100,`70%`=(`70_Sum`/nrow(Control_Batch))*100
,`65%`=(`65_Sum`/nrow(Control_Batch))*100)

OUR_Trigger_Count_Summary<-cbind(OUR_DPI_Trigger_Total[,c(1,2,7)],OUR_Trigger_Count)
%>%

mutate(Identifier=paste(paste(`Trigger_Value_%`,"%",sep=""),`Slope_Value`,OUR_Label,
sep="_"))%>%

  group_by(Identifier)%>%

summarize(`95_Sum`=sum(`95`,na.rm=TRUE),`90_Sum`=sum(`90`,na.rm=TRUE),`85_Sum`=sum(`
85`,na.rm=TRUE),`80_Sum`=sum(`80`,na.rm=TRUE),`75_Sum`=sum(`75`,na.rm=TRUE),`70_Sum`
=sum(`70`,na.rm=TRUE),`65_Sum`=sum(`65`,na.rm=TRUE),`Average_%_Peak_VII`=mean(Percen
t_Peak_Cont_VII,na.rm=TRUE),`SD_%_Peak_VII`=sd(Percent_Peak_Cont_VII,na.rm=TRUE))%>%

mutate(`95%`=(`95_Sum`/nrow(Control_Batch))*100,`90%`=(`90_Sum`/nrow(Control_Batch))
*100,`85%`=(`85_Sum`/nrow(Control_Batch))*100,`80%`=(`80_Sum`/nrow(Control_Batch))*1
00,`75%`=(`75_Sum`/nrow(Control_Batch))*100,`70%`=(`70_Sum`/nrow(Control_Batch))*100
,`65%`=(`65_Sum`/nrow(Control_Batch))*100)

DO_DPI_Trigger_Total<-cbind(DO_DPI_Trigger_Total,DO_Trigger_Count[,c(8,10)])

VCV_DPI_Trigger_Total<-cbind(VCV_DPI_Trigger_Total,VCV_Trigger_Count[,c(8,10)])

OUR_DPI_Trigger_Total<-cbind(OUR_DPI_Trigger_Total,OUR_Trigger_Count[,c(8,10)])
```

```
Summary_List<-list(DO_Trigger_Count_Summary,VCV_Trigger_Count_Summary,OUR_Trigger_Co
unt_Summary)

Summary_Names<-c("DO_Trigger_Count_Summary","VCV_Trigger_Count_Summary","OUR_Trigger
_Count_Summary")

names(Summary_List)<-Summary_Names

write_xlsx(Summary_List,"Trigger_Count_Summary.xlsx")



Total_List<-list(DO_DPI_Trigger_Total,VCV_DPI_Trigger_Total,OUR_DPI_Trigger_Total)

Total_Names<-c("DO_DPI_Trigger_Total","VCV_DPI_Trigger_Total","OUR_DPI_Trigger_Total
")

names(Total_List)<-Total_Names

write_xlsx(Total_List,"Trigger_Data_Total.xlsx")




#### Plotting Results ####

pdf(file='Trigger_Count_Summary_Blinded.pdf',width=17.04,height=11.04)

for (i in 1:nrow(VII_Percentiles))

{

  if (i==1)

  {


a<-DO_Trigger_Count_Summary%>%mutate(Identifier=fct_reorder(Identifier,desc(`95%`),.
fun='length'))%>%


ggplot(aes(x=reorder(Identifier,desc(`95%`)),y=`95%`,fill=Identifier))+geom_col(widt
h=0.8)+


theme(legend.position="none",axis.text.x=element_blank(),axis.title.x=element_blank(
```

```
),axis.ticks.x=element_blank())+

      labs(

        title=paste("Percent of Batches Predicted Within",
paste(VII_Percentiles[c(i),],"%",sep=""), "of Peak Titer",sep=" "),

        y=paste("Percentage within",  paste(VII_Percentiles[c(i),],"%",sep=""), "of
Peak Titer",sep=" "),

        fill="Trigger"

      )+

      ylim(0,100)



b<-VCV_Trigger_Count_Summary%>%mutate(Identifier=fct_reorder(Identifier,desc(`95%`),
.fun='length'))%>%


ggplot(aes(x=reorder(Identifier,desc(`95%`)),y=`95%`,fill=Identifier))+geom_col(widt
h=0.8)+


theme(legend.position="none",axis.text.x=element_blank(),axis.title.x=element_blank(
),axis.ticks.x=element_blank())+

      labs(

        title=paste("Percent of Batches Predicted Within",
paste(VII_Percentiles[c(i),],"%",sep=""), "of Peak Titer",sep=" "),

        y=paste("Percentage within",  paste(VII_Percentiles[c(i),],"%",sep=""), "of
Peak Titer",sep=" "),

        fill="Trigger"

      )+

      ylim(0,100)



c<-OUR_Trigger_Count_Summary%>%mutate(Identifier=fct_reorder(Identifier,desc(`95%`),
.fun='length'))%>%
```

```
ggplot(aes(x=reorder(Identifier,desc(`95%`)),y=`95%`,fill=Identifier))+geom_col(widt
h=0.8)+

theme(legend.position="none",axis.text.x=element_blank(),axis.title.x=element_blank(
),axis.ticks.x=element_blank())+

      labs(

        title=paste("Percent of Batches Predicted Within",
paste(VII_Percentiles[c(i),],"%",sep=""), "of Peak Titer",sep=" "),

        y=paste("Percentage within",  paste(VII_Percentiles[c(i),],"%",sep=""), "of
Peak Titer",sep=" "),

        fill="Trigger"

      )+

      ylim(0,100)



    d<-ggplot()+

      geom_point(data=Mean_Merge_DO_Data,aes(DO_x_Mean,y=DO_y_Mean))+

geom_ribbon(data=Mean_Merge_DO_Data,aes(ymin=DO_y_Mean-DO_y_SD,ymax=DO_y_Mean+DO_y_S
D,x=DO_x_Mean),fill="grey46",alpha=0.2)+

      geom_vline(xintercept=1.2,color="red")+

      geom_text(x=0.8,y=57,aes(label="Trigger 1"),color="red",size=5)+

      geom_vline(xintercept=1.8,color="red")+

      geom_text(x=2.3,y=80,aes(label="Trigger 2"),color="red",size=5)+

      geom_vline(xintercept=2.8,color="red")+

      geom_text(x=3.3,y=100,aes(label="Trigger 3"),color="red",size=5)+

      theme(legend.position="none",axis.text.y=element_blank())+

      labs(

        title = "Average DO Trend",
```

```
        x="Days Post Infection (DPI)",
        y="DO (%)"
      )

    e<-ggplot()+
      geom_point(data=Mean_Merge_VCV_Data,aes(VCV_x_Mean,y=VCV_y_Mean))+
geom_ribbon(data=Mean_Merge_VCV_Data,aes(ymin=VCV_y_Mean-VCV_y_SD,ymax=VCV_y_Mean+VCV_y_SD,x=VCV_x_Mean),fill="grey46",alpha=0.2)+
      geom_vline(xintercept=1,color="red")+
      geom_text(x=0.5,y=3.3e8,aes(label="Trigger 1"),color="red",size=5)+
      geom_vline(xintercept=1.6,color="red")+
      geom_text(x=2.1,y=2.05e8,aes(label="Trigger 2"),color="red",size=5)+
      geom_vline(xintercept=2.6,color="red")+
      geom_text(x=3.2,y=1.2e8,aes(label="Trigger 3"),color="red",size=5)+
      theme(legend.position="none",axis.text.y=element_blank())+
      labs(
        title = "Average VCV Trend",
        x="Days Post Infection (DPI)",
        y="VCV (viable cells*um3/mL)"
      )

    f<-ggplot()+
      geom_point(data=Mean_Merge_OUR_Data,aes(OUR_x_Mean,y=OUR_y_Mean))+
geom_ribbon(data=Mean_Merge_OUR_Data,aes(ymin=OUR_y_Mean-OUR_y_SD,ymax=OUR_y_Mean+OUR_y_SD,x=OUR_x_Mean),fill="grey46",alpha=0.2)+
```

```
      geom_vline(xintercept=1.7,color="red")+
      geom_text(x=1.2,y=4.2e-6,aes(label="Trigger 1"),color="red",size=5)+
      geom_vline(xintercept=2,color="red")+
      geom_text(x=2.5,y=2.4e-6,aes(label="Trigger 2"),color="red",size=5)+
      geom_vline(xintercept=3,color="red")+
      geom_text(x=3.5,y=1.3e-6,aes(label="Trigger 3"),color="red",size=5)+
      theme(legend.position="none",axis.text.y=element_blank())+
      labs(
        title = "Average OUR Trend",
        x="Days Post Infection (DPI)",
        y="OUR (uM O2/((viable cells*um3)/mL))"
      )

    g<-ggarrange(a,b,c,d,e,f,
                 labels=c("A","B","C"),
                 ncol=3,nrow=2)
    print(g)
  }else if (i==2)
  {

a<-DO_Trigger_Count_Summary%>%mutate(Identifier=fct_reorder(Identifier,desc(`90%`),.
fun='length'))%>%

ggplot(aes(x=reorder(Identifier,desc(`90%`)),y=`90%`,fill=Identifier))+geom_col(widt
h=0.8)+

theme(legend.position="none",axis.text.x=element_blank(),axis.title.x=element_blank(
),axis.ticks.x=element_blank())+
```

```
      labs(

        title=paste("Percent of Batches Predicted Within",
paste(VII_Percentiles[c(i),],"%",sep=""), "of Peak Titer",sep=" "),

        y=paste("Percentage within",  paste(VII_Percentiles[c(i),],"%",sep=""), "of
Peak Titer",sep=" "),

        fill="Trigger"

      )+

      ylim(0,100)



b<-VCV_Trigger_Count_Summary%>%mutate(Identifier=fct_reorder(Identifier,desc(`90%`),
.fun='length'))%>%


ggplot(aes(x=reorder(Identifier,desc(`90%`)),y=`90%`,fill=Identifier))+geom_col(widt
h=0.8)+


theme(legend.position="none",axis.text.x=element_blank(),axis.title.x=element_blank(
),axis.ticks.x=element_blank())+

      labs(

        title=paste("Percent of Batches Predicted Within",
paste(VII_Percentiles[c(i),],"%",sep=""), "of Peak Titer",sep=" "),

        y=paste("Percentage within",  paste(VII_Percentiles[c(i),],"%",sep=""), "of
Peak Titer",sep=" "),

        fill="Trigger"

      )+

      ylim(0,100)



c<-OUR_Trigger_Count_Summary%>%mutate(Identifier=fct_reorder(Identifier,desc(`90%`),
.fun='length'))%>%


ggplot(aes(x=reorder(Identifier,desc(`90%`)),y=`90%`,fill=Identifier))+geom_col(widt
```

```
h=0.8)+

theme(legend.position="none",axis.text.x=element_blank(),axis.title.x=element_blank(
),axis.ticks.x=element_blank())+

      labs(

        title=paste("Percent of Batches Predicted Within",
paste(VII_Percentiles[c(i),],"%",sep=""), "of Peak Titer",sep=" "),

        y=paste("Percentage within",  paste(VII_Percentiles[c(i),],"%",sep=""), "of
Peak Titer",sep=" "),

        fill="Trigger"

      )+

      ylim(0,100)



    d<-ggplot()+

      geom_point(data=Mean_Merge_DO_Data,aes(DO_x_Mean,y=DO_y_Mean))+

geom_ribbon(data=Mean_Merge_DO_Data,aes(ymin=DO_y_Mean-DO_y_SD,ymax=DO_y_Mean+DO_y_S
D,x=DO_x_Mean),fill="grey46",alpha=0.2)+

      geom_vline(xintercept=1.2,color="red")+

      geom_text(x=0.8,y=57,aes(label="Trigger 1"),color="red",size=5)+

      geom_vline(xintercept=1.8,color="red")+

      geom_text(x=2.3,y=80,aes(label="Trigger 2"),color="red",size=5)+

      geom_vline(xintercept=2.8,color="red")+

      geom_text(x=3.3,y=100,aes(label="Trigger 3"),color="red",size=5)+

      theme(legend.position="none",axis.text.y=element_blank())+

      labs(

        title = "Average DO Trend",

        x="Days Post Infection (DPI)",
```

```
        y="DO (%)"
      )

    e<-ggplot()+
      geom_point(data=Mean_Merge_VCV_Data,aes(VCV_x_Mean,y=VCV_y_Mean))+
geom_ribbon(data=Mean_Merge_VCV_Data,aes(ymin=VCV_y_Mean-VCV_y_SD,ymax=VCV_y_Mean+VC
V_y_SD,x=VCV_x_Mean),fill="grey46",alpha=0.2)+
      geom_vline(xintercept=1,color="red")+
      geom_text(x=0.5,y=3.3e8,aes(label="Trigger 1"),color="red",size=5)+
      geom_vline(xintercept=1.6,color="red")+
      geom_text(x=2.1,y=2.05e8,aes(label="Trigger 2"),color="red",size=5)+
      geom_vline(xintercept=2.6,color="red")+
      geom_text(x=3.2,y=1.2e8,aes(label="Trigger 3"),color="red",size=5)+
      theme(legend.position="none",axis.text.y=element_blank())+
      labs(
        title = "Average VCV Trend",
        x="Days Post Infection (DPI)",
        y="VCV (viable cells*um3/mL)"
      )

    f<-ggplot()+
      geom_point(data=Mean_Merge_OUR_Data,aes(OUR_x_Mean,y=OUR_y_Mean))+
geom_ribbon(data=Mean_Merge_OUR_Data,aes(ymin=OUR_y_Mean-OUR_y_SD,ymax=OUR_y_Mean+OU
R_y_SD,x=OUR_x_Mean),fill="grey46",alpha=0.2)+
      geom_vline(xintercept=1.7,color="red")+
```

```
      geom_text(x=1.2,y=4.2e-6,aes(label="Trigger 1"),color="red",size=5)+
      geom_vline(xintercept=2,color="red")+
      geom_text(x=2.5,y=2.4e-6,aes(label="Trigger 2"),color="red",size=5)+
      geom_vline(xintercept=3,color="red")+
      geom_text(x=3.5,y=1.3e-6,aes(label="Trigger 3"),color="red",size=5)+
      theme(legend.position="none",axis.text.y=element_blank())+
      labs(
        title = "Average OUR Trend",
        x="Days Post Infection (DPI)",
        y="OUR (uM O2/((viable cells*um3)/mL))"
      )

    g<-ggarrange(a,b,c,d,e,f,
                 labels=c("A","B","C"),
                 ncol=3,nrow=2)
    print(g)
  }else if (i==3)
  {

a<-DO_Trigger_Count_Summary%>%mutate(Identifier=fct_reorder(Identifier,desc(`85%`),.
fun='length'))%>%

ggplot(aes(x=reorder(Identifier,desc(`85%`)),y=`85%`,fill=Identifier))+geom_col(widt
h=0.8)+

theme(legend.position="none",axis.text.x=element_blank(),axis.title.x=element_blank(
),axis.ticks.x=element_blank())+
      labs(
```

```
        title=paste("Percent of Batches Predicted Within",
paste(VII_Percentiles[c(i),],"%",sep=""), "of Peak Titer",sep=" "),

        y=paste("Percentage within",  paste(VII_Percentiles[c(i),],"%",sep=""), "of
Peak Titer",sep=" "),

        fill="Trigger"

      )+

      ylim(0,100)



b<-VCV_Trigger_Count_Summary%>%mutate(Identifier=fct_reorder(Identifier,desc(`85%`),
.fun='length'))%>%


ggplot(aes(x=reorder(Identifier,desc(`85%`)),y=`85%`,fill=Identifier))+geom_col(widt
h=0.8)+


theme(legend.position="none",axis.text.x=element_blank(),axis.title.x=element_blank(
),axis.ticks.x=element_blank())+

      labs(

        title=paste("Percent of Batches Predicted Within",
paste(VII_Percentiles[c(i),],"%",sep=""), "of Peak Titer",sep=" "),

        y=paste("Percentage within",  paste(VII_Percentiles[c(i),],"%",sep=""), "of
Peak Titer",sep=" "),

        fill="Trigger"

      )+

      ylim(0,100)



c<-OUR_Trigger_Count_Summary%>%mutate(Identifier=fct_reorder(Identifier,desc(`85%`),
.fun='length'))%>%


ggplot(aes(x=reorder(Identifier,desc(`85%`)),y=`85%`,fill=Identifier))+geom_col(widt
h=0.8)+
```

```
theme(legend.position="none",axis.text.x=element_blank(),axis.title.x=element_blank(
),axis.ticks.x=element_blank())+

      labs(

        title=paste("Percent of Batches Predicted Within",
paste(VII_Percentiles[c(i),],"%",sep=""), "of Peak Titer",sep=" "),

        y=paste("Percentage within",  paste(VII_Percentiles[c(i),],"%",sep=""), "of
Peak Titer",sep=" "),

        fill="Trigger"

      )+

      ylim(0,100)



    d<-ggplot()+

      geom_point(data=Mean_Merge_DO_Data,aes(DO_x_Mean,y=DO_y_Mean))+


geom_ribbon(data=Mean_Merge_DO_Data,aes(ymin=DO_y_Mean-DO_y_SD,ymax=DO_y_Mean+DO_y_S
D,x=DO_x_Mean),fill="grey46",alpha=0.2)+

      geom_vline(xintercept=1.2,color="red")+

      geom_text(x=0.8,y=57,aes(label="Trigger 1"),color="red",size=5)+

      geom_vline(xintercept=1.8,color="red")+

      geom_text(x=2.3,y=80,aes(label="Trigger 2"),color="red",size=5)+

      geom_vline(xintercept=2.8,color="red")+

      geom_text(x=3.3,y=100,aes(label="Trigger 3"),color="red",size=5)+

      theme(legend.position="none",axis.text.y=element_blank())+

      labs(

        title = "Average DO Trend",

        x="Days Post Infection (DPI)",

        y="DO (%)"
```

```
      )


    e<-ggplot()+
      geom_point(data=Mean_Merge_VCV_Data,aes(VCV_x_Mean,y=VCV_y_Mean))+
geom_ribbon(data=Mean_Merge_VCV_Data,aes(ymin=VCV_y_Mean-VCV_y_SD,ymax=VCV_y_Mean+VC
V_y_SD,x=VCV_x_Mean),fill="grey46",alpha=0.2)+
      geom_vline(xintercept=1,color="red")+
      geom_text(x=0.5,y=3.3e8,aes(label="Trigger 1"),color="red",size=5)+
      geom_vline(xintercept=1.6,color="red")+
      geom_text(x=2.1,y=2.05e8,aes(label="Trigger 2"),color="red",size=5)+
      geom_vline(xintercept=2.6,color="red")+
      geom_text(x=3.2,y=1.2e8,aes(label="Trigger 3"),color="red",size=5)+
      theme(legend.position="none",axis.text.y=element_blank())+
      labs(
        title = "Average VCV Trend",
        x="Days Post Infection (DPI)",
        y="VCV (viable cells*um3/mL)"
      )


    f<-ggplot()+
      geom_point(data=Mean_Merge_OUR_Data,aes(OUR_x_Mean,y=OUR_y_Mean))+
geom_ribbon(data=Mean_Merge_OUR_Data,aes(ymin=OUR_y_Mean-OUR_y_SD,ymax=OUR_y_Mean+OU
R_y_SD,x=OUR_x_Mean),fill="grey46",alpha=0.2)+
      geom_vline(xintercept=1.7,color="red")+
      geom_text(x=1.2,y=4.2e-6,aes(label="Trigger 1"),color="red",size=5)+
```

```
      geom_vline(xintercept=2,color="red")+
      geom_text(x=2.5,y=2.4e-6,aes(label="Trigger 2"),color="red",size=5)+
      geom_vline(xintercept=3,color="red")+
      geom_text(x=3.5,y=1.3e-6,aes(label="Trigger 3"),color="red",size=5)+
      theme(legend.position="none",axis.text.y=element_blank())+
      labs(
        title = "Average OUR Trend",
        x="Days Post Infection (DPI)",
        y="OUR (uM O2/((viable cells*um3)/mL))"
      )

    g<-ggarrange(a,b,c,d,e,f,
                 labels=c("A","B","C"),
                 ncol=3,nrow=2)
    print(g)
  }else if (i==4)
  {

a<-DO_Trigger_Count_Summary%>%mutate(Identifier=fct_reorder(Identifier,desc(`80%`),.fun='length'))%>%

ggplot(aes(x=reorder(Identifier,desc(`80%`)),y=`80%`,fill=Identifier))+geom_col(width=0.8)+

theme(legend.position="none",axis.text.x=element_blank(),axis.title.x=element_blank(),axis.ticks.x=element_blank())+
      labs(
        title=paste("Percent of Batches Predicted Within", paste(VII_Percentiles[c(i),],"%",sep=""), "of Peak Titer",sep=" "),
```

```
        y=paste("Percentage within",  paste(VII_Percentiles[c(i),],"%",sep=""), "of
Peak Titer",sep=" "),

        fill="Trigger"

      )+

      ylim(0,100)



b<-VCV_Trigger_Count_Summary%>%mutate(Identifier=fct_reorder(Identifier,desc(`80%`),
.fun='length'))%>%

ggplot(aes(x=reorder(Identifier,desc(`80%`)),y=`80%`,fill=Identifier))+geom_col(widt
h=0.8)+

theme(legend.position="none",axis.text.x=element_blank(),axis.title.x=element_blank(
),axis.ticks.x=element_blank())+

      labs(

        title=paste("Percent of Batches Predicted Within",
paste(VII_Percentiles[c(i),],"%",sep=""), "of Peak Titer",sep=" "),

        y=paste("Percentage within",  paste(VII_Percentiles[c(i),],"%",sep=""), "of
Peak Titer",sep=" "),

        fill="Trigger"

      )+

      ylim(0,100)



c<-OUR_Trigger_Count_Summary%>%mutate(Identifier=fct_reorder(Identifier,desc(`80%`),
.fun='length'))%>%

ggplot(aes(x=reorder(Identifier,desc(`80%`)),y=`80%`,fill=Identifier))+geom_col(widt
h=0.8)+

theme(legend.position="none",axis.text.x=element_blank(),axis.title.x=element_blank(
```

```
),axis.ticks.x=element_blank())+

      labs(

        title=paste("Percent of Batches Predicted Within",
paste(VII_Percentiles[c(i),],"%",sep=""), "of Peak Titer",sep=" "),

        y=paste("Percentage within",  paste(VII_Percentiles[c(i),],"%",sep=""), "of
Peak Titer",sep=" "),

        fill="Trigger"

      )+

      ylim(0,100)



    d<-ggplot()+

      geom_point(data=Mean_Merge_DO_Data,aes(DO_x_Mean,y=DO_y_Mean))+


geom_ribbon(data=Mean_Merge_DO_Data,aes(ymin=DO_y_Mean-DO_y_SD,ymax=DO_y_Mean+DO_y_S
D,x=DO_x_Mean),fill="grey46",alpha=0.2)+

      geom_vline(xintercept=1.2,color="red")+

      geom_text(x=0.8,y=57,aes(label="Trigger 1"),color="red",size=5)+

      geom_vline(xintercept=1.8,color="red")+

      geom_text(x=2.3,y=80,aes(label="Trigger 2"),color="red",size=5)+

      geom_vline(xintercept=2.8,color="red")+

      geom_text(x=3.3,y=100,aes(label="Trigger 3"),color="red",size=5)+

      theme(legend.position="none",axis.text.y=element_blank())+

      labs(

        title = "Average DO Trend",

        x="Days Post Infection (DPI)",

        y="DO (%)"

      )
```

```
e<-ggplot()+
  geom_point(data=Mean_Merge_VCV_Data,aes(VCV_x_Mean,y=VCV_y_Mean))+
geom_ribbon(data=Mean_Merge_VCV_Data,aes(ymin=VCV_y_Mean-VCV_y_SD,ymax=VCV_y_Mean+VC
V_y_SD,x=VCV_x_Mean),fill="grey46",alpha=0.2)+
  geom_vline(xintercept=1,color="red")+
  geom_text(x=0.5,y=3.3e8,aes(label="Trigger 1"),color="red",size=5)+
  geom_vline(xintercept=1.6,color="red")+
  geom_text(x=2.1,y=2.05e8,aes(label="Trigger 2"),color="red",size=5)+
  geom_vline(xintercept=2.6,color="red")+
  geom_text(x=3.2,y=1.2e8,aes(label="Trigger 3"),color="red",size=5)+
  theme(legend.position="none",axis.text.y=element_blank())+
  labs(
    title = "Average VCV Trend",
    x="Days Post Infection (DPI)",
    y="VCV (viable cells*um3/mL)"
  )

f<-ggplot()+
  geom_point(data=Mean_Merge_OUR_Data,aes(OUR_x_Mean,y=OUR_y_Mean))+
geom_ribbon(data=Mean_Merge_OUR_Data,aes(ymin=OUR_y_Mean-OUR_y_SD,ymax=OUR_y_Mean+OU
R_y_SD,x=OUR_x_Mean),fill="grey46",alpha=0.2)+
  geom_vline(xintercept=1.7,color="red")+
  geom_text(x=1.2,y=4.2e-6,aes(label="Trigger 1"),color="red",size=5)+
  geom_vline(xintercept=2,color="red")+
```

```
      geom_text(x=2.5,y=2.4e-6,aes(label="Trigger 2"),color="red",size=5)+

      geom_vline(xintercept=3,color="red")+

      geom_text(x=3.5,y=1.3e-6,aes(label="Trigger 3"),color="red",size=5)+

      theme(legend.position="none",axis.text.y=element_blank())+

      labs(

        title = "Average OUR Trend",

        x="Days Post Infection (DPI)",

        y="OUR (uM O2/((viable cells*um3)/mL))"

      )


    g<-ggarrange(a,b,c,d,e,f,

                 labels=c("A","B","C"),

                 ncol=3,nrow=2)

    print(g)

  }else if (i==5)

  {

a<-DO_Trigger_Count_Summary%>%mutate(Identifier=fct_reorder(Identifier,desc(`75%`),.
fun='length'))%>%

ggplot(aes(x=reorder(Identifier,desc(`75%`)),y=`75%`,fill=Identifier))+geom_col(widt
h=0.8)+

theme(legend.position="none",axis.text.x=element_blank(),axis.title.x=element_blank(
),axis.ticks.x=element_blank())+

      labs(

        title=paste("Percent of Batches Predicted Within",
paste(VII_Percentiles[c(i),],"%",sep=""), "of Peak Titer",sep=" "),

        y=paste("Percentage within",  paste(VII_Percentiles[c(i),],"%",sep=""), "of
```

```
Peak Titer",sep=" "),

        fill="Trigger"

      )+

      ylim(0,100)



b<-VCV_Trigger_Count_Summary%>%mutate(Identifier=fct_reorder(Identifier,desc(`75%`),
.fun='length'))%>%


ggplot(aes(x=reorder(Identifier,desc(`75%`)),y=`75%`,fill=Identifier))+geom_col(widt
h=0.8)+


theme(legend.position="none",axis.text.x=element_blank(),axis.title.x=element_blank(
),axis.ticks.x=element_blank())+

      labs(

        title=paste("Percent of Batches Predicted Within",
paste(VII_Percentiles[c(i),],"%",sep=""), "of Peak Titer",sep=" "),

        y=paste("Percentage within",  paste(VII_Percentiles[c(i),],"%",sep=""), "of
Peak Titer",sep=" "),

        fill="Trigger"

      )+

      ylim(0,100)



c<-OUR_Trigger_Count_Summary%>%mutate(Identifier=fct_reorder(Identifier,desc(`75%`),
.fun='length'))%>%


ggplot(aes(x=reorder(Identifier,desc(`75%`)),y=`75%`,fill=Identifier))+geom_col(widt
h=0.8)+


theme(legend.position="none",axis.text.x=element_blank(),axis.title.x=element_blank(
),axis.ticks.x=element_blank())+
```

```
      labs(
        title=paste("Percent of Batches Predicted Within",
paste(VII_Percentiles[c(i),],"%",sep=""), "of Peak Titer",sep=" "),
        y=paste("Percentage within",  paste(VII_Percentiles[c(i),],"%",sep=""), "of
Peak Titer",sep=" "),
        fill="Trigger"
      )+
      ylim(0,100)


    d<-ggplot()+
      geom_point(data=Mean_Merge_DO_Data,aes(DO_x_Mean,y=DO_y_Mean))+

geom_ribbon(data=Mean_Merge_DO_Data,aes(ymin=DO_y_Mean-DO_y_SD,ymax=DO_y_Mean+DO_y_S
D,x=DO_x_Mean),fill="grey46",alpha=0.2)+
      geom_vline(xintercept=1.2,color="red")+
      geom_text(x=0.8,y=57,aes(label="Trigger 1"),color="red",size=5)+
      geom_vline(xintercept=1.8,color="red")+
      geom_text(x=2.3,y=80,aes(label="Trigger 2"),color="red",size=5)+
      geom_vline(xintercept=2.8,color="red")+
      geom_text(x=3.3,y=100,aes(label="Trigger 3"),color="red",size=5)+
      theme(legend.position="none",axis.text.y=element_blank())+
      labs(
        title = "Average DO Trend",
        x="Days Post Infection (DPI)",
        y="DO (%)"
      )
```

```
e<-ggplot()+
  geom_point(data=Mean_Merge_VCV_Data,aes(VCV_x_Mean,y=VCV_y_Mean))+
geom_ribbon(data=Mean_Merge_VCV_Data,aes(ymin=VCV_y_Mean-VCV_y_SD,ymax=VCV_y_Mean+VC
V_y_SD,x=VCV_x_Mean),fill="grey46",alpha=0.2)+
  geom_vline(xintercept=1,color="red")+
  geom_text(x=0.5,y=3.3e8,aes(label="Trigger 1"),color="red",size=5)+
  geom_vline(xintercept=1.6,color="red")+
  geom_text(x=2.1,y=2.05e8,aes(label="Trigger 2"),color="red",size=5)+
  geom_vline(xintercept=2.6,color="red")+
  geom_text(x=3.2,y=1.2e8,aes(label="Trigger 3"),color="red",size=5)+
  theme(legend.position="none",axis.text.y=element_blank())+
  labs(
    title = "Average VCV Trend",
    x="Days Post Infection (DPI)",
    y="VCV (viable cells*um3/mL)"
  )

f<-ggplot()+
  geom_point(data=Mean_Merge_OUR_Data,aes(OUR_x_Mean,y=OUR_y_Mean))+
geom_ribbon(data=Mean_Merge_OUR_Data,aes(ymin=OUR_y_Mean-OUR_y_SD,ymax=OUR_y_Mean+OU
R_y_SD,x=OUR_x_Mean),fill="grey46",alpha=0.2)+
  geom_vline(xintercept=1.7,color="red")+
  geom_text(x=1.2,y=4.2e-6,aes(label="Trigger 1"),color="red",size=5)+
  geom_vline(xintercept=2,color="red")+
  geom_text(x=2.5,y=2.4e-6,aes(label="Trigger 2"),color="red",size=5)+
```

```
      geom_vline(xintercept=3,color="red")+

      geom_text(x=3.5,y=1.3e-6,aes(label="Trigger 3"),color="red",size=5)+

      theme(legend.position="none",axis.text.y=element_blank())+

      labs(

        title = "Average OUR Trend",

        x="Days Post Infection (DPI)",

        y="OUR (uM O2/((viable cells*um3)/mL))"

      )


    g<-ggarrange(a,b,c,d,e,f,

                 labels=c("A","B","C"),

                 ncol=3,nrow=2)

    print(g)

  }else if (i==6)

  {

a<-DO_Trigger_Count_Summary%>%mutate(Identifier=fct_reorder(Identifier,desc(`70%`),.
fun='length'))%>%

ggplot(aes(x=reorder(Identifier,desc(`70%`)),y=`70%`,fill=Identifier))+geom_col(widt
h=0.8)+

theme(legend.position="none",axis.text.x=element_blank(),axis.title.x=element_blank(
),axis.ticks.x=element_blank())+

      labs(

        title=paste("Percent of Batches Predicted Within",
paste(VII_Percentiles[c(i),],"%",sep=""), "of Peak Titer",sep=" "),

        y=paste("Percentage within",  paste(VII_Percentiles[c(i),],"%",sep=""), "of
Peak Titer",sep=" "),
```

```
        fill="Trigger"

      )+

      ylim(0,100)



b<-VCV_Trigger_Count_Summary%>%mutate(Identifier=fct_reorder(Identifier,desc(`70%`),
.fun='length'))%>%


ggplot(aes(x=reorder(Identifier,desc(`70%`)),y=`70%`,fill=Identifier))+geom_col(widt
h=0.8)+


theme(legend.position="none",axis.text.x=element_blank(),axis.title.x=element_blank(
),axis.ticks.x=element_blank())+

      labs(

        title=paste("Percent of Batches Predicted Within",
paste(VII_Percentiles[c(i),],"%",sep=""), "of Peak Titer",sep=" "),

        y=paste("Percentage within",  paste(VII_Percentiles[c(i),],"%",sep=""), "of
Peak Titer",sep=" "),

        fill="Trigger"

      )+

      ylim(0,100)



c<-OUR_Trigger_Count_Summary%>%mutate(Identifier=fct_reorder(Identifier,desc(`70%`),
.fun='length'))%>%


ggplot(aes(x=reorder(Identifier,desc(`70%`)),y=`70%`,fill=Identifier))+geom_col(widt
h=0.8)+


theme(legend.position="none",axis.text.x=element_blank(),axis.title.x=element_blank(
),axis.ticks.x=element_blank())+

      labs(
```

```
        title=paste("Percent of Batches Predicted Within",
paste(VII_Percentiles[c(i),],"%",sep=""), "of Peak Titer",sep=" "),

        y=paste("Percentage within",  paste(VII_Percentiles[c(i),],"%",sep=""), "of
Peak Titer",sep=" "),

        fill="Trigger"

      )+

      ylim(0,100)



    d<-ggplot()+

      geom_point(data=Mean_Merge_DO_Data,aes(DO_x_Mean,y=DO_y_Mean))+


geom_ribbon(data=Mean_Merge_DO_Data,aes(ymin=DO_y_Mean-DO_y_SD,ymax=DO_y_Mean+DO_y_S
D,x=DO_x_Mean),fill="grey46",alpha=0.2)+

      geom_vline(xintercept=1.2,color="red")+

      geom_text(x=0.8,y=57,aes(label="Trigger 1"),color="red",size=5)+

      geom_vline(xintercept=1.8,color="red")+

      geom_text(x=2.3,y=80,aes(label="Trigger 2"),color="red",size=5)+

      geom_vline(xintercept=2.8,color="red")+

      geom_text(x=3.3,y=100,aes(label="Trigger 3"),color="red",size=5)+

      theme(legend.position="none",axis.text.y=element_blank())+

      labs(

        title = "Average DO Trend",

        x="Days Post Infection (DPI)",

        y="DO (%)"

      )



    e<-ggplot()+
```

```
      geom_point(data=Mean_Merge_VCV_Data,aes(VCV_x_Mean,y=VCV_y_Mean))+

geom_ribbon(data=Mean_Merge_VCV_Data,aes(ymin=VCV_y_Mean-VCV_y_SD,ymax=VCV_y_Mean+VC
V_y_SD,x=VCV_x_Mean),fill="grey46",alpha=0.2)+

      geom_vline(xintercept=1,color="red")+

      geom_text(x=0.5,y=3.3e8,aes(label="Trigger 1"),color="red",size=5)+

      geom_vline(xintercept=1.6,color="red")+

      geom_text(x=2.1,y=2.05e8,aes(label="Trigger 2"),color="red",size=5)+

      geom_vline(xintercept=2.6,color="red")+

      geom_text(x=3.2,y=1.2e8,aes(label="Trigger 3"),color="red",size=5)+

      theme(legend.position="none",axis.text.y=element_blank())+

      labs(

        title = "Average VCV Trend",

        x="Days Post Infection (DPI)",

        y="VCV (viable cells*um3/mL)"

      )


    f<-ggplot()+

      geom_point(data=Mean_Merge_OUR_Data,aes(OUR_x_Mean,y=OUR_y_Mean))+

geom_ribbon(data=Mean_Merge_OUR_Data,aes(ymin=OUR_y_Mean-OUR_y_SD,ymax=OUR_y_Mean+OU
R_y_SD,x=OUR_x_Mean),fill="grey46",alpha=0.2)+

      geom_vline(xintercept=1.7,color="red")+

      geom_text(x=1.2,y=4.2e-6,aes(label="Trigger 1"),color="red",size=5)+

      geom_vline(xintercept=2,color="red")+

      geom_text(x=2.5,y=2.4e-6,aes(label="Trigger 2"),color="red",size=5)+

      geom_vline(xintercept=3,color="red")+
```

```
      geom_text(x=3.5,y=1.3e-6,aes(label="Trigger 3"),color="red",size=5)+

      theme(legend.position="none",axis.text.y=element_blank())+

      labs(

        title = "Average OUR Trend",

        x="Days Post Infection (DPI)",

        y="OUR (uM O2/((viable cells*um3)/mL))"

      )


    g<-ggarrange(a,b,c,d,e,f,

                 labels=c("A","B","C"),

                 ncol=3,nrow=2)

    print(g)

  }else if (i==7)

  {

a<-DO_Trigger_Count_Summary%>%mutate(Identifier=fct_reorder(Identifier,desc(`65%`),.
fun='length'))%>%


ggplot(aes(x=reorder(Identifier,desc(`65%`)),y=`65%`,fill=Identifier))+geom_col(widt
h=0.8)+


theme(legend.position="none",axis.text.x=element_blank(),axis.title.x=element_blank(
),axis.ticks.x=element_blank())+

      labs(

        title=paste("Percent of Batches Predicted Within",
paste(VII_Percentiles[c(i),],"%",sep=""), "of Peak Titer",sep=" "),

        y=paste("Percentage within",  paste(VII_Percentiles[c(i),],"%",sep=""), "of
Peak Titer",sep=" "),

        fill="Trigger"
```

```
      )+

      ylim(0,100)



b<-VCV_Trigger_Count_Summary%>%mutate(Identifier=fct_reorder(Identifier,desc(`65%`),
.fun='length'))%>%


ggplot(aes(x=reorder(Identifier,desc(`65%`)),y=`65%`,fill=Identifier))+geom_col(widt
h=0.8)+


theme(legend.position="none",axis.text.x=element_blank(),axis.title.x=element_blank(
),axis.ticks.x=element_blank())+

      labs(

        title=paste("Percent of Batches Predicted Within",
paste(VII_Percentiles[c(i),],"%",sep=""), "of Peak Titer",sep=" "),

        y=paste("Percentage within",  paste(VII_Percentiles[c(i),],"%",sep=""), "of
Peak Titer",sep=" "),

        fill="Trigger"

      )+

      ylim(0,100)



c<-OUR_Trigger_Count_Summary%>%mutate(Identifier=fct_reorder(Identifier,desc(`65%`),
.fun='length'))%>%


ggplot(aes(x=reorder(Identifier,desc(`65%`)),y=`65%`,fill=Identifier))+geom_col(widt
h=0.8)+


theme(legend.position="none",axis.text.x=element_blank(),axis.title.x=element_blank(
),axis.ticks.x=element_blank())+

      labs(

        title=paste("Percent of Batches Predicted Within",
paste(VII_Percentiles[c(i),],"%",sep=""), "of Peak Titer",sep=" "),
```

```
        y=paste("Percentage within",  paste(VII_Percentiles[c(i),],"%",sep=""), "of
Peak Titer",sep=" "),

        fill="Trigger"

      )+

      ylim(0,100)



    d<-ggplot()+

      geom_point(data=Mean_Merge_DO_Data,aes(DO_x_Mean,y=DO_y_Mean))+


geom_ribbon(data=Mean_Merge_DO_Data,aes(ymin=DO_y_Mean-DO_y_SD,ymax=DO_y_Mean+DO_y_S
D,x=DO_x_Mean),fill="grey46",alpha=0.2)+

      geom_vline(xintercept=1.2,color="red")+

      geom_text(x=0.8,y=57,aes(label="Trigger 1"),color="red",size=5)+

      geom_vline(xintercept=1.8,color="red")+

      geom_text(x=2.3,y=80,aes(label="Trigger 2"),color="red",size=5)+

      geom_vline(xintercept=2.8,color="red")+

      geom_text(x=3.3,y=100,aes(label="Trigger 3"),color="red",size=5)+

      theme(legend.position="none",axis.text.y=element_blank())+

      labs(

        title = "Average DO Trend",

        x="Days Post Infection (DPI)",

        y="DO (%)"

      )



    e<-ggplot()+

      geom_point(data=Mean_Merge_VCV_Data,aes(VCV_x_Mean,y=VCV_y_Mean))+
```

```
geom_ribbon(data=Mean_Merge_VCV_Data,aes(ymin=VCV_y_Mean-VCV_y_SD,ymax=VCV_y_Mean+VC
V_y_SD,x=VCV_x_Mean),fill="grey46",alpha=0.2)+
      geom_vline(xintercept=1,color="red")+
      geom_text(x=0.5,y=3.3e8,aes(label="Trigger 1"),color="red",size=5)+
      geom_vline(xintercept=1.6,color="red")+
      geom_text(x=2.1,y=2.05e8,aes(label="Trigger 2"),color="red",size=5)+
      geom_vline(xintercept=2.6,color="red")+
      geom_text(x=3.2,y=1.2e8,aes(label="Trigger 3"),color="red",size=5)+
      theme(legend.position="none",axis.text.y=element_blank())+
      labs(
        title = "Average VCV Trend",
        x="Days Post Infection (DPI)",
        y="VCV (viable cells*um3/mL)"
      )

    f<-ggplot()+
      geom_point(data=Mean_Merge_OUR_Data,aes(OUR_x_Mean,y=OUR_y_Mean))+
geom_ribbon(data=Mean_Merge_OUR_Data,aes(ymin=OUR_y_Mean-OUR_y_SD,ymax=OUR_y_Mean+OU
R_y_SD,x=OUR_x_Mean),fill="grey46",alpha=0.2)+
      geom_vline(xintercept=1.7,color="red")+
      geom_text(x=1.2,y=4.2e-6,aes(label="Trigger 1"),color="red",size=5)+
      geom_vline(xintercept=2,color="red")+
      geom_text(x=2.5,y=2.4e-6,aes(label="Trigger 2"),color="red",size=5)+
      geom_vline(xintercept=3,color="red")+
      geom_text(x=3.5,y=1.3e-6,aes(label="Trigger 3"),color="red",size=5)+
```

```
      theme(legend.position="none",axis.text.y=element_blank())+
      labs(
        title = "Average OUR Trend",
        x="Days Post Infection (DPI)",
        y="OUR (uM O2/((viable cells*um3)/mL))"
      )

    g<-ggarrange(a,b,c,d,e,f,
                 labels=c("A","B","C"),
                 ncol=3,nrow=2)
    print(g)
  }
}

dev.off()


test<-as.data.frame(t(DO_Trigger_Count_Summary))
colnames(test)<-c(test[c(1),])
test<-test[-c(1:10),]
test<-add_column(test,VII_Percentiles,.before="10%_DO_DPI_Trigger_1")
test2<-pivot_longer(test,cols=c(colnames(test[,-c(1)])),names_to="Identifier",values
_to="Percent")
test2[,c(3)]<-as.numeric(unlist(test2[,c(3)]))
test2[,c(1)]<-as.numeric(unlist(test2[,c(1)]))
test2<-test2%>%group_by(Percentiles)%>%arrange(-Percentiles,-Percent)%>%mutate(rank=
```

```
1:n())

p<-test2%>%ggplot()+aes(xmin=1,xmax=20,x=rank)+aes(ymin=0,ymax=1.2,y=Percent)+facet_
wrap(~Percentiles)+geom_col()+aes(fill=Identifier)

p+facet_null()+geom_text(x=10,y=1.1,aes(label=paste("Fraction predicted
within",paste(Percentiles,"%",sep=""),"of Peak Titer",sep="
")),size=5)+aes(group=Identifier)+transition_states(Percentiles)

anim_save("Percentiles_Rank_DO.gif")



test<-as.data.frame(t(VCV_Trigger_Count_Summary))

colnames(test)<-c(test[c(1),])

test<-test[-c(1:10),]

test<-add_column(test,VII_Percentiles,.before="10%_VCV_DPI_Trigger_1")

test2<-pivot_longer(test,cols=c(colnames(test[,-c(1)])),names_to="Identifier",values
_to="Percent")

test2[,c(3)]<-as.numeric(unlist(test2[,c(3)]))

test2[,c(1)]<-as.numeric(unlist(test2[,c(1)]))

test2<-test2%>%group_by(Percentiles)%>%arrange(-Percentiles,-Percent)%>%mutate(rank=
1:n())

p<-test2%>%ggplot()+aes(xmin=1,xmax=20,x=rank)+aes(ymin=0,ymax=1.2,y=Percent)+facet_
wrap(~Percentiles)+geom_col()+aes(fill=Identifier)

p+facet_null()+geom_text(x=10,y=1.1,aes(label=paste("Fraction predicted
within",paste(Percentiles,"%",sep=""),"of Peak Titer",sep="
")),size=5)+aes(group=Identifier)+transition_states(Percentiles)

anim_save("Percentiles_Rank_VCV.gif")



test<-as.data.frame(t(OUR_Trigger_Count_Summary))

colnames(test)<-c(test[c(1),])

test<-test[-c(1:10),]

test<-add_column(test,VII_Percentiles,.before="10%_OUR_DPI_Trigger_1")
```

```
test2<-pivot_longer(test,cols=c(colnames(test[,-c(1)])),names_to="Identifier",values
_to="Percent")

test2[,c(3)]<-as.numeric(unlist(test2[,c(3)]))

test2[,c(1)]<-as.numeric(unlist(test2[,c(1)]))

test2<-test2%>%group_by(Percentiles)%>%arrange(-Percentiles,-Percent)%>%mutate(rank=
1:n())

p<-test2%>%ggplot()+aes(xmin=1,xmax=20,x=rank)+aes(ymin=0,ymax=1.2,y=Percent)+facet_
wrap(~Percentiles)+geom_col()+aes(fill=Identifier)

p+facet_null()+geom_text(x=10,y=1.1,aes(label=paste("Fraction predicted
within",paste(Percentiles,"%",sep=""),"of Peak Titer",sep="
")),size=5)+aes(group=Identifier)+transition_states(Percentiles)

anim_save("Percentiles_Rank_OUR.gif")




# d<-ggplot()+

#   geom_point(data=Mean_Merge_DO_Data,aes(DO_x_Mean,y=DO_y_Mean))+

#
geom_ribbon(data=Mean_Merge_DO_Data,aes(ymin=DO_y_Mean-DO_y_SD,ymax=DO_y_Mean+DO_y_S
D,x=DO_x_Mean),fill="grey46",alpha=0.1)+

#
geom_point(data=Mean_Merge_VII_Data,aes(VII_x_Mean,y=VII_y_Mean/1e7),color="red")+

#
geom_ribbon(data=Mean_Merge_VII_Data,aes(ymin=(VII_y_Mean-VII_y_SD)/1e7,ymax=(VII_y_
Mean+VII_y_SD)/1e7,x=VII_x_Mean),fill="tomato1",alpha=0.1)+

#   scale_y_continuous("DO_y_Mean",sec.axis=sec_axis(~.*1e7,name="VII Titer"))+

#   labs(

#     title = "Average DO + VII Trend",

#   )
```

```
#### Non-Control Batch Calculations ####

Num_Batch_ID<-nrow(Non_Control_Batch)

VII_Percentiles<-data.frame(array(c(95,90,85,80,75,70,65)))

colnames(VII_Percentiles)<-c("Percentiles")

Peak_VII<-as.data.frame(matrix(ncol=5))

colnames(Peak_VII)<-c("Peak_Cont_VII_DPI","Peak_Cont_VII","Peak_VII_DPI","Peak_VII",
"Batch_ID")

Percentile_Store<-data.frame(matrix(ncol=1))



for (l in 1:Num_Batch_ID)

{

  h<-filter(OUR_Data_Extended,`Batch
ID`==as.character(Non_Control_Batch[l,])&`Infection?`=="INFECTION")


Temp_Filter_Cont_VII_Full<-h%>%filter(Step_100_row_VII==max(Step_100_row_VII,na.rm=T
RUE))

  Temp_Filter_VII_Full<-h%>%filter(VII==max(VII,na.rm=TRUE))

  Peak_VII[c(l),c(1:2)]<-Temp_Filter_Cont_VII_Full[,c("DPI","Step_100_row_VII")]

  Peak_VII[c(l),c(3:5)]<-Temp_Filter_VII_Full[,c("DPI","VII","Batch ID")]

  if (l==2)

  {

    Blank_rows<-data.frame(matrix(ncol=29,nrow=(nrow(Non_Control_Batch)-1)))


Percentile_Store<-rbind(Percentile_Store,setNames(Blank_rows,names(Percentile_Store)
))

  }

  for (i in 1:nrow(VII_Percentiles))

  {
```

```
Temp_Percentile_Minus<-h%>%filter(Step_100_row_VII<(Peak_VII[c(l),c(2)]-((100-VII_Percentiles[c(i),])/100)*Peak_VII[c(l),c(2)])&DPI<Peak_VII[c(l),c(1)])

Temp_Percentile_Plus<-h%>%filter(Step_100_row_VII<(Peak_VII[c(l),c(2)]-((100-VII_Percentiles[c(i),])/100)*Peak_VII[c(l),c(2)])&DPI>Peak_VII[c(l),c(1)])


    if (l==1)

    {

      Varname_Plus_DPI<-paste("+",VII_Percentiles[c(i),],"th"," DPI",sep="")

      Varname_Minus_DPI<-paste("-",VII_Percentiles[c(i),],"th"," DPI",sep="")

      Varname_Plus_Value<-paste("+",VII_Percentiles[c(i),],"th"," Value",sep="")

      Varname_Minus_Value<-paste("-",VII_Percentiles[c(i),],"th"," Value",sep="")

Percentile_Store[[Varname_Minus_DPI]]<-tail(Temp_Percentile_Minus[,c("DPI")],n=1)

Percentile_Store[[Varname_Minus_Value]]<-tail(Temp_Percentile_Minus[,c("Step_100_row_VII")],n=1)

      Percentile_Store[[Varname_Plus_DPI]]<-Temp_Percentile_Plus[c(1),c("DPI")]

Percentile_Store[[Varname_Plus_Value]]<-Temp_Percentile_Plus[c(1),c("Step_100_row_VII")]

    }else

    {

      Varname_Plus_DPI<-paste("+",VII_Percentiles[c(i),],"th"," DPI",sep="")

      Varname_Minus_DPI<-paste("-",VII_Percentiles[c(i),],"th"," DPI",sep="")

      Varname_Plus_Value<-paste("+",VII_Percentiles[c(i),],"th"," Value",sep="")

      Varname_Minus_Value<-paste("-",VII_Percentiles[c(i),],"th"," Value",sep="")

Percentile_Store[c(l),c(Varname_Minus_DPI)]<-tail(Temp_Percentile_Minus[,c("DPI")],n=1)
```

```
Percentile_Store[c(l),c(Varname_Minus_Value)]<-tail(Temp_Percentile_Minus[,c("Step_1
00_row_VII")],n=1)

Percentile_Store[c(l),c(Varname_Plus_DPI)]<-Temp_Percentile_Plus[c(1),c("DPI")]

Percentile_Store[c(l),c(Varname_Plus_Value)]<-Temp_Percentile_Plus[c(1),c("Step_100_
row_VII")]

    }

  }

}

Peak_VII<-cbind(Peak_VII,Percentile_Store[,-c(1)])

#### Trigger 1 Calculations ####

Value_Trigger<-data.frame(array(c(10,15,20,25,30,35,40)))

colnames(Value_Trigger)<-c("Trigger Value Percent")

DPI_Trigger<-0.5

Derivative_Trigger<-matrix(c(0, -5e7, -5e-7,

                             5, -1e7, -1e-6,

                             15, -2e8, -5e-6,

                             25, -1.5e8, -8e-6,

                             30, -8e7, -1.5e-6),nrow=5,ncol=3,byrow=TRUE)

colnames(Derivative_Trigger)<-c("DO_Derivative","VCV_Derivative","OUR_Derivative")

DO_x_Data<-as.data.frame(matrix(ncol=Num_Batch_ID))

DO_y_Data<-as.data.frame(matrix(ncol=Num_Batch_ID))
```

```
DO_DPI_Trigger_Full_1<-as.data.frame(matrix(ncol=4))

colnames(DO_DPI_Trigger_Full_1)<-c("DO_DPI_Trigger","DO%_Trigger","dDO_Trigger","Bat
ch_ID")



VCV_x_Data<-as.data.frame(matrix(ncol=Num_Batch_ID))

VCV_y_Data<-as.data.frame(matrix(ncol=Num_Batch_ID))

VCV_DPI_Trigger_Full_1<-as.data.frame(matrix(ncol=4))

colnames(VCV_DPI_Trigger_Full_1)<-c("VCV_DPI_Trigger","VCV_Trigger","dVCV_Trigger","
Batch_ID")



OUR_x_Data<-as.data.frame(matrix(ncol=Num_Batch_ID))

OUR_y_Data<-as.data.frame(matrix(ncol=Num_Batch_ID))

OUR_DPI_Trigger_Full_1<-as.data.frame(matrix(ncol=4))

colnames(OUR_DPI_Trigger_Full_1)<-c("OUR_DPI_Trigger","OUR_Trigger","dOUR_Trigger","
Batch_ID")



VII_x_Data<-as.data.frame(matrix(ncol=Num_Batch_ID))

VII_y_Data<-as.data.frame(matrix(ncol=Num_Batch_ID))



for (a in 1:nrow(Value_Trigger))

{

  for (i in 1:nrow(Derivative_Trigger))

  {

    for (l in 1:Num_Batch_ID)

    {

      h<-filter(OUR_Data_Extended,`Batch
ID`==as.character(Non_Control_Batch[l,])&`Infection?`=="INFECTION")
```

```
f<-filter(OUR_Data_Extended,`Batch ID`==as.character(Non_Control_Batch[l,]))

if (a==1&&i==1)

{

  n=nrow(h)

  DO_x_Data[1:n,c(l)]<-h[,"DPI"]

  DO_y_Data[1:n,c(l)]<-h[,"Avg DO hr (%)"]


  VCV_x_Data[1:n,c(l)]<-h[,"DPI"]

  VCV_y_Data[1:n,c(l)]<-h[,"Avg VCV hr (um3 cells)"]


  OUR_x_Data[1:n,c(l)]<-h[,"DPI"]

  OUR_y_Data[1:n,c(l)]<-h[,"Avg OUR hr (/um3 cells) min"]


  VII_x_Data[1:n,c(l)]<-h[,"DPI"]

  VII_y_Data[1:n,c(l)]<-h[,"Step_100_row_VII"]

}


#setting up empty matrices and filling them with batch data

DO_Data_Trim<-as.data.frame(matrix(ncol=3))

colnames(DO_Data_Trim)<-c("DPI","DO%","dDO%")

DO_Data_Trim[1:nrow(f),]<-f[,(c("DPI","Avg DO hr (%)","dDO (%)"))]


VCV_Data_Trim<-as.data.frame(matrix(ncol=3))

colnames(VCV_Data_Trim)<-c("DPI","VCV","dVCV")

VCV_Data_Trim[1:nrow(f),]<-f[,(c("DPI","Avg VCV hr (um3 cells)","dVCV (um3
cells)"))]
```

```
	OUR_Data_Trim<-as.data.frame(matrix(ncol=3))

	colnames(OUR_Data_Trim)<-c("DPI","OUR","dOUR")

	OUR_Data_Trim[1:nrow(f),]<-f[,(c("DPI","Avg OUR hr (/um3 cells) min","dOUR
(/um3 cells) min" ))]



	#Max and Min DO calculations


Max_DO<-DO_Data_Trim%>%filter(DPI<(-0.5))%>%filter(`DO%`==max(`DO%`,na.rm=TRUE))

	Max_DO<-Max_DO[,c(2)]

	# if (round(Max_DO[,c(1)],0)==(-3))

	# {

	#   Max_DO<-Max_DO[,c(2)]

	# }else

	# {

	#
Max_DO<-DO_Data_Trim%>%filter(DPI>3&`dDO%`<10)%>%filter(`DO%`==max(`DO%`,na.rm=TRUE)
)

	#   Max_DO<-Max_DO[,c(2)]

	#   if (is_empty(Max_DO))

	#   {

	#
Max_DO<-DO_Data_Trim%>%filter(DPI>2)%>%filter(`DO%`==max(`DO%`,na.rm=TRUE))

	#     Max_DO<-Max_DO[,c(2)]

	#   }

	# }


Min_DO<-DO_Data_Trim%>%filter(DPI<(-0.1)&`DO%`>0)%>%filter(`DO%`==min(`DO%`,na.rm=TR
```

```
UE))
      Min_DO<-Min_DO[,c(2)]


      #Max and min VCV calculations

Max_VCV<-VCV_Data_Trim%>%filter(DPI<3.5&DPI>0.5)%>%filter(VCV==max(VCV,na.rm=TRUE))
      Max_VCV<-Max_VCV[,c(2)]
      Min_VCV<-VCV_Data_Trim%>%filter(DPI<(-1))%>%filter(VCV==min(VCV,na.rm=TRUE))
      Min_VCV<-Min_VCV[,c(2)]
      # if (round(Min_VCV[,c(1)],0)==(-3))
      # {
      #   Min_VCV<-Min_VCV[,c(2)]
      # }else
      # {
      #
Min_VCV<-VCV_Data_Trim%>%filter(DPI>2.5&dVCV>(-1e7)|DPI<(-1))%>%filter(VCV==min(VCV,
na.rm=TRUE))
      #   Min_VCV<-Min_VCV[,c(2)]
      #   if (is_empty(Min_VCV))
      #   {
      #     Min_VCV<-VCV_Data_Trim%>%filter()
      #   }
      # }


      #Max and min OUR calculations
      Max_OUR<-OUR_Data_Trim%>%filter(DPI<(-0.5))%>%filter(OUR==max(OUR,na.rm=TRUE))
      Max_OUR<-Max_OUR[,c(2)]
```

```
      # if(round(Max_OUR[,c(1)],1)<3&round(Max_OUR[,c(1)],1)>0)
      # {
      #   Max_OUR<-Max_OUR[,c(2)]
      # }else
      # {
      #
Max_OUR<-OUR_Data_Trim%>%filter(DPI>0|DPI<(-0.5))%>%filter(DPI<3&dOUR<1e-8)%>%filter
(OUR==max(OUR,na.rm=TRUE))
      #   Max_OUR<-Max_OUR[,c(2)]
      #   if(is_empty(Max_OUR))
      #   {
      #     Max_OUR<-OUR_Data_Trim%>%filter()
      #   }
      # }
      Min_OUR<-OUR_Data_Trim%>%filter(DPI<(-1))%>%filter(OUR==min(OUR,na.rm=TRUE))
      Min_OUR<-Min_OUR[,c(2)]
      # if (round(Min_OUR[,c(1)],0)==(-3))
      # {
      #   Min_OUR<-Min_OUR[,c(2)]
      # }else
      # {
      #
Min_OUR<-OUR_Data_Trim%>%filter(DPI>2.5&dOUR>(-1e-8)|DPI<(-1))%>%filter(OUR==min(OUR
,na.rm=TRUE))
      #   Min_OUR<-Min_OUR[,c(2)]
      #   if (is_empty(Min_OUR))
      #   {
```

```
      #     Min_OUR<-OUR_Data_Trim%>%filter()

      #   }

      # }


      #Filtering for Trigger


Temp_Filter_DO_Full<-DO_Data_Trim%>%filter(DPI>DPI_Trigger)%>%filter(`dDO%`>Derivati
ve_Trigger[i,1])%>%filter(`DO%`>Min_DO+(Max_DO-Min_DO)*(Value_Trigger[c(a),]/100))


Temp_Filter_VCV_Full<-VCV_Data_Trim%>%filter(DPI>DPI_Trigger)%>%filter(dVCV<Derivati
ve_Trigger[i,2])%>%filter(VCV<Max_VCV-(Max_VCV-Min_VCV)*(Value_Trigger[c(a),]/100))


Temp_Filter_OUR_Full<-OUR_Data_Trim%>%filter(DPI>DPI_Trigger)%>%filter(dOUR<Derivati
ve_Trigger[i,3])%>%filter(OUR<Max_OUR-(Max_OUR-Min_OUR)*(Value_Trigger[c(a),]/100))




      #Storing Trigger Data

      if (a==1&&i==1)

      {

        DO_DPI_Trigger_Full_1[c(l),c(1:3)]<-Temp_Filter_DO_Full[c(1),]

        DO_DPI_Trigger_Full_1[c(l),c(4)]<-Non_Control_Batch[l,]


DO_DPI_Trigger_Full_1<-DO_DPI_Trigger_Full_1%>%mutate(DO_Label="DO_DPI_Trigger_1",`T
rigger_Value_%`=Value_Trigger[c(a),],`Slope_Value`=Derivative_Trigger[i,1])

        DO_row<-nrow(DO_DPI_Trigger_Full_1)


        VCV_DPI_Trigger_Full_1[c(l),c(1:3)]<-Temp_Filter_VCV_Full[c(1),]

        VCV_DPI_Trigger_Full_1[c(l),c(4)]<-Non_Control_Batch[l,]
```

```
VCV_DPI_Trigger_Full_1<-VCV_DPI_Trigger_Full_1%>%mutate(VCV_Label="VCV_DPI_Trigger_1
",`Trigger_Value_%`=Value_Trigger[c(a),],`Slope_Value`=Derivative_Trigger[i,2])

        VCV_row<-nrow(VCV_DPI_Trigger_Full_1)


        OUR_DPI_Trigger_Full_1[c(l),c(1:3)]<-Temp_Filter_OUR_Full[c(1),]

        OUR_DPI_Trigger_Full_1[c(l),c(4)]<-Non_Control_Batch[l,]

OUR_DPI_Trigger_Full_1<-OUR_DPI_Trigger_Full_1%>%mutate(OUR_Label="OUR_DPI_Trigger_1
",`Trigger_Value_%`=Value_Trigger[c(a),],`Slope_Value`=Derivative_Trigger[i,3])

        OUR_row<-nrow(OUR_DPI_Trigger_Full_1)


      }else

      {

        DO_DPI_Trigger_Full_1[c(DO_row+l),c(1:3)]<-Temp_Filter_DO_Full[c(1),]

        DO_DPI_Trigger_Full_1[c(DO_row+l),c(4)]<-Non_Control_Batch[l,]

        DO_DPI_Trigger_Full_1[c(DO_row+l),c(5)]<-c("DO_DPI_Trigger_1")

        DO_DPI_Trigger_Full_1[c(DO_row+l),c(6)]<-Value_Trigger[c(a),]

        DO_DPI_Trigger_Full_1[c(DO_row+l),c(7)]<-Derivative_Trigger[i,1]


        VCV_DPI_Trigger_Full_1[c(VCV_row+l),c(1:3)]<-Temp_Filter_VCV_Full[c(1),]

        VCV_DPI_Trigger_Full_1[c(VCV_row+l),c(4)]<-Non_Control_Batch[l,]

        VCV_DPI_Trigger_Full_1[c(VCV_row+l),c(5)]<-c("VCV_DPI_Trigger_1")

        VCV_DPI_Trigger_Full_1[c(VCV_row+l),c(6)]<-Value_Trigger[c(a),]

        VCV_DPI_Trigger_Full_1[c(VCV_row+l),c(7)]<-Derivative_Trigger[i,2]


        OUR_DPI_Trigger_Full_1[c(OUR_row+l),c(1:3)]<-Temp_Filter_OUR_Full[c(1),]

        OUR_DPI_Trigger_Full_1[c(OUR_row+l),c(4)]<-Non_Control_Batch[l,]
```

```
        OUR_DPI_Trigger_Full_1[c(OUR_row+l),c(5)]<-c("OUR_DPI_Trigger_1")
        OUR_DPI_Trigger_Full_1[c(OUR_row+l),c(6)]<-Value_Trigger[c(a),]
        OUR_DPI_Trigger_Full_1[c(OUR_row+l),c(7)]<-Derivative_Trigger[i,3]
      }
    }
    #Row numbers for storing trigger data
    DO_row<-nrow(DO_DPI_Trigger_Full_1)
    VCV_row<-nrow(VCV_DPI_Trigger_Full_1)
    OUR_row<-nrow(OUR_DPI_Trigger_Full_1)
  }
}

# #Average DO trends
# colnames(DO_x_Data)<-c(paste(unlist(transpose(Non_Control_Batch)),'x',sep="_"))
# colnames(DO_y_Data)<-c(paste(unlist(transpose(Non_Control_Batch)),'y',sep="_"))
# DO_x_Mean<-rowMeans(DO_x_Data,na.rm=TRUE)
# DO_y_Mean<-rowMeans(DO_y_Data,na.rm=TRUE)
# DO_x_SD<-apply(DO_x_Data,1,sd,na.rm=TRUE)
# DO_y_SD<-apply(DO_y_Data,1,sd,na.rm=TRUE)
# DO_x_Data<-cbind(DO_x_Data,DO_x_Mean,DO_x_SD)
# DO_y_Data<-cbind(DO_y_Data,DO_y_Mean,DO_y_SD)
# Mean_Merge_DO_Data<-cbind(DO_x_Data[,c(8:9)],DO_y_Data[,c(8:9)])
#
# #Average VCV trends
# colnames(VCV_x_Data)<-c(paste(unlist(transpose(Non_Control_Batch)),'x',sep="_"))
```

```
# colnames(VCV_y_Data)<-c(paste(unlist(transpose(Non_Control_Batch)),'y',sep="_"))

# VCV_x_Mean<-rowMeans(VCV_x_Data,na.rm=TRUE)

# VCV_y_Mean<-rowMeans(VCV_y_Data,na.rm=TRUE)

# VCV_x_SD<-apply(VCV_x_Data,1,sd,na.rm=TRUE)

# VCV_y_SD<-apply(VCV_y_Data,1,sd,na.rm=TRUE)

# VCV_x_Data<-cbind(VCV_x_Data,VCV_x_Mean,VCV_x_SD)

# VCV_y_Data<-cbind(VCV_y_Data,VCV_y_Mean,VCV_y_SD)

# Mean_Merge_VCV_Data<-cbind(VCV_x_Data[,c(8:9)],VCV_y_Data[,c(8:9)])

#

# #Average OUR trends

# colnames(OUR_x_Data)<-c(paste(unlist(transpose(Non_Control_Batch)),'x',sep="_"))

# colnames(OUR_y_Data)<-c(paste(unlist(transpose(Non_Control_Batch)),'y',sep="_"))

# OUR_x_Mean<-rowMeans(OUR_x_Data,na.rm=TRUE)

# OUR_y_Mean<-rowMeans(OUR_y_Data,na.rm=TRUE)

# OUR_x_SD<-apply(OUR_x_Data,1,sd,na.rm=TRUE)

# OUR_y_SD<-apply(OUR_y_Data,1,sd,na.rm=TRUE)

# OUR_x_Data<-cbind(OUR_x_Data,OUR_x_Mean,OUR_x_SD)

# OUR_y_Data<-cbind(OUR_y_Data,OUR_y_Mean,OUR_y_SD)

# Mean_Merge_OUR_Data<-cbind(OUR_x_Data[,c(8:9)],OUR_y_Data[,c(8:9)])

#

# #Average VII Trends

# colnames(VII_x_Data)<-c(paste(unlist(transpose(Non_Control_Batch)),'x',sep="_"))

# colnames(VII_y_Data)<-c(paste(unlist(transpose(Non_Control_Batch)),'y',sep="_"))

# VII_x_Mean<-rowMeans(VII_x_Data,na.rm=TRUE)

# VII_y_Mean<-rowMeans(VII_y_Data,na.rm=TRUE)
```

```
# VII_x_SD<-apply(VII_x_Data,1,sd,na.rm=TRUE)

# VII_y_SD<-apply(VII_y_Data,1,sd,na.rm=TRUE)

# VII_x_Data<-cbind(VII_x_Data,VII_x_Mean,VII_x_SD)

# VII_y_Data<-cbind(VII_y_Data,VII_y_Mean,VII_y_SD)

# Mean_Merge_VII_Data<-cbind(VII_x_Data[,c(8:9)],VII_y_Data[,c(8:9)])


#### Trigger 2 Calculations ####

Value_Trigger_2<-data.frame(array(c(30,35,40,45,50,55,60)))

colnames(Value_Trigger_2)<-c("Trigger Value Percent")

DPI_Trigger_2<-1

Derivative_Trigger_2<-matrix(c(20, -5e7, -3e-6,

                               25, -1e7, -5e-6,

                               35, -1e8, -8e-6,

                               45, -1.5e8, -1e-5,

                               50, -8e7, -2e-6),nrow=5,ncol=3,byrow=TRUE)

colnames(Derivative_Trigger_2)<-c("DO_Derivative","VCV_Derivative","OUR_Derivative")


DO_DPI_Trigger_Full_2<-as.data.frame(matrix(ncol=4))

colnames(DO_DPI_Trigger_Full_2)<-c("DO_DPI_Trigger","DO%_Trigger","dDO_Trigger","Bat
ch_ID")


VCV_DPI_Trigger_Full_2<-as.data.frame(matrix(ncol=4))

colnames(VCV_DPI_Trigger_Full_2)<-c("VCV_DPI_Trigger","VCV_Trigger","dVCV_Trigger","
Batch_ID")


OUR_DPI_Trigger_Full_2<-as.data.frame(matrix(ncol=4))
```

```
colnames(OUR_DPI_Trigger_Full_2)<-c("OUR_DPI_Trigger","OUR_Trigger","dOUR_Trigger","
Batch_ID")


for (a in 1:nrow(Value_Trigger_2))

{

  for (i in 1:nrow(Derivative_Trigger_2))

  {

    for (l in 1:Num_Batch_ID)

    {

      h<-filter(OUR_Data_Extended,`Batch
ID`==as.character(Non_Control_Batch[l,])&`Infection?`=="INFECTION")

      f<-filter(OUR_Data_Extended,`Batch ID`==as.character(Non_Control_Batch[l,]))



      #setting up empty matrices and filling them with batch data

      DO_Data_Trim<-as.data.frame(matrix(ncol=3))

      colnames(DO_Data_Trim)<-c("DPI","DO%","dDO%")

      DO_Data_Trim[1:nrow(f),]<-f[,(c("DPI","Avg DO hr (%)","dDO (%)"))]



      VCV_Data_Trim<-as.data.frame(matrix(ncol=3))

      colnames(VCV_Data_Trim)<-c("DPI","VCV","dVCV")

      VCV_Data_Trim[1:nrow(f),]<-f[,(c("DPI","Avg VCV hr (um3 cells)","dVCV (um3
cells)"))]



      OUR_Data_Trim<-as.data.frame(matrix(ncol=3))

      colnames(OUR_Data_Trim)<-c("DPI","OUR","dOUR")

      OUR_Data_Trim[1:nrow(f),]<-f[,(c("DPI","Avg OUR hr (/um3 cells) min","dOUR
(/um3 cells) min" ))]
```

```
    #Max and Min DO calculations

Max_DO<-DO_Data_Trim%>%filter(DPI<(-0.5))%>%filter(`DO%`==max(`DO%`,na.rm=TRUE))

    Max_DO<-Max_DO[,c(2)]

    # if (round(Max_DO[,c(1)],0)==(-3))

    # {

    #   Max_DO<-Max_DO[,c(2)]

    # }else

    # {

    #
Max_DO<-DO_Data_Trim%>%filter(DPI>3&`dDO%`<10)%>%filter(`DO%`==max(`DO%`,na.rm=TRUE)
)

    #   Max_DO<-Max_DO[,c(2)]

    #   if (is_empty(Max_DO))

    #   {

    #
Max_DO<-DO_Data_Trim%>%filter(DPI>2)%>%filter(`DO%`==max(`DO%`,na.rm=TRUE))

    #     Max_DO<-Max_DO[,c(2)]

    #   }

    # }

Min_DO<-DO_Data_Trim%>%filter(DPI<(-0.1)&`DO%`>0)%>%filter(`DO%`==min(`DO%`,na.rm=TR
UE))

    Min_DO<-Min_DO[,c(2)]



    #Max and min VCV calculations
```

```
Max_VCV<-VCV_Data_Trim%>%filter(DPI<3.5&DPI>0.5)%>%filter(VCV==max(VCV,na.rm=TRUE))

      Max_VCV<-Max_VCV[,c(2)]

      Min_VCV<-VCV_Data_Trim%>%filter(DPI<(-1))%>%filter(VCV==min(VCV,na.rm=TRUE))

      Min_VCV<-Min_VCV[,c(2)]

      # if (round(Min_VCV[,c(1)],0)==(-3))

      # {

      #   Min_VCV<-Min_VCV[,c(2)]

      # }else

      # {

      #
Min_VCV<-VCV_Data_Trim%>%filter(DPI>2.5&dVCV>(-1e7)|DPI<(-1))%>%filter(VCV==min(VCV,
na.rm=TRUE))

      #   Min_VCV<-Min_VCV[,c(2)]

      #   if (is_empty(Min_VCV))

      #   {

      #     Min_VCV<-VCV_Data_Trim%>%filter()

      #   }

      # }


      #Max and min OUR calculations

      Max_OUR<-OUR_Data_Trim%>%filter(DPI<(-0.5))%>%filter(OUR==max(OUR,na.rm=TRUE))

      Max_OUR<-Max_OUR[,c(2)]

      # if(round(Max_OUR[,c(1)],1)<3&round(Max_OUR[,c(1)],1)>0)

      # {

      #   Max_OUR<-Max_OUR[,c(2)]

      # }else
```

```
# {

#
Max_OUR<-OUR_Data_Trim%>%filter(DPI>0|DPI<(-0.5))%>%filter(DPI<3&dOUR<1e-8)%>%filter
(OUR==max(OUR,na.rm=TRUE))

#   Max_OUR<-Max_OUR[,c(2)]

#   if(is_empty(Max_OUR))

#   {

#     Max_OUR<-OUR_Data_Trim%>%filter()

#   }

# }

Min_OUR<-OUR_Data_Trim%>%filter(DPI<(-1))%>%filter(OUR==min(OUR,na.rm=TRUE))

Min_OUR<-Min_OUR[,c(2)]

# if (round(Min_OUR[,c(1)],0)==(-3))

# {

#   Min_OUR<-Min_OUR[,c(2)]

# }else

# {

#
Min_OUR<-OUR_Data_Trim%>%filter(DPI>2.5&dOUR>(-1e-8)|DPI<(-1))%>%filter(OUR==min(OUR
,na.rm=TRUE))

#   Min_OUR<-Min_OUR[,c(2)]

#   if (is_empty(Min_OUR))

#   {

#     Min_OUR<-OUR_Data_Trim%>%filter()

#   }

# }
```

```
      #Filtering for Trigger

Temp_Filter_DO_Full<-DO_Data_Trim%>%filter(DPI>DPI_Trigger_2)%>%filter(`dDO%`>Deriva
tive_Trigger_2[i,1])%>%filter(`DO%`>Min_DO+(Max_DO-Min_DO)*(Value_Trigger_2[c(a),]/1
00))

Temp_Filter_VCV_Full<-VCV_Data_Trim%>%filter(DPI>DPI_Trigger_2)%>%filter(dVCV<Deriva
tive_Trigger_2[i,2])%>%filter(VCV<Max_VCV-(Max_VCV-Min_VCV)*(Value_Trigger_2[c(a),]/
100))

Temp_Filter_OUR_Full<-OUR_Data_Trim%>%filter(DPI>DPI_Trigger_2)%>%filter(dOUR<Deriva
tive_Trigger_2[i,3])%>%filter(OUR<Max_OUR-(Max_OUR-Min_OUR)*(Value_Trigger_2[c(a),]/
100))



      #Storing Trigger Data

      if (a==1&i==1)

      {

        DO_DPI_Trigger_Full_2[c(l),c(1:3)]<-Temp_Filter_DO_Full[c(1),]

        DO_DPI_Trigger_Full_2[c(l),c(4)]<-Non_Control_Batch[l,]

DO_DPI_Trigger_Full_2<-DO_DPI_Trigger_Full_2%>%mutate(DO_Label="DO_DPI_Trigger_2",`T
rigger_Value_%`=Value_Trigger_2[c(a),],`Slope_Value`=Derivative_Trigger_2[i,1])

        DO_row<-nrow(DO_DPI_Trigger_Full_2)


        VCV_DPI_Trigger_Full_2[c(l),c(1:3)]<-Temp_Filter_VCV_Full[c(1),]

        VCV_DPI_Trigger_Full_2[c(l),c(4)]<-Non_Control_Batch[l,]

VCV_DPI_Trigger_Full_2<-VCV_DPI_Trigger_Full_2%>%mutate(VCV_Label="VCV_DPI_Trigger_2
",`Trigger_Value_%`=Value_Trigger_2[c(a),],`Slope_Value`=Derivative_Trigger_2[i,2])

        VCV_row<-nrow(VCV_DPI_Trigger_Full_2)
```

```
        OUR_DPI_Trigger_Full_2[c(l),c(1:3)]<-Temp_Filter_OUR_Full[c(1),]

        OUR_DPI_Trigger_Full_2[c(l),c(4)]<-Non_Control_Batch[l,]

OUR_DPI_Trigger_Full_2<-OUR_DPI_Trigger_Full_2%>%mutate(OUR_Label="OUR_DPI_Trigger_2
",`Trigger_Value_%`=Value_Trigger_2[c(a),],`Slope_Value`=Derivative_Trigger_2[i,3])

        OUR_row<-nrow(OUR_DPI_Trigger_Full_2)


      }else

      {

        DO_DPI_Trigger_Full_2[c(DO_row+l),c(1:3)]<-Temp_Filter_DO_Full[c(1),]

        DO_DPI_Trigger_Full_2[c(DO_row+l),c(4)]<-Non_Control_Batch[l,]

        DO_DPI_Trigger_Full_2[c(DO_row+l),c(5)]<-c("DO_DPI_Trigger_2")

        DO_DPI_Trigger_Full_2[c(DO_row+l),c(6)]<-Value_Trigger_2[c(a),]

        DO_DPI_Trigger_Full_2[c(DO_row+l),c(7)]<-Derivative_Trigger_2[i,1]


        VCV_DPI_Trigger_Full_2[c(VCV_row+l),c(1:3)]<-Temp_Filter_VCV_Full[c(1),]

        VCV_DPI_Trigger_Full_2[c(VCV_row+l),c(4)]<-Non_Control_Batch[l,]

        VCV_DPI_Trigger_Full_2[c(VCV_row+l),c(5)]<-c("VCV_DPI_Trigger_2")

        VCV_DPI_Trigger_Full_2[c(VCV_row+l),c(6)]<-Value_Trigger_2[c(a),]

        VCV_DPI_Trigger_Full_2[c(VCV_row+l),c(7)]<-Derivative_Trigger_2[i,2]


        OUR_DPI_Trigger_Full_2[c(OUR_row+l),c(1:3)]<-Temp_Filter_OUR_Full[c(1),]

        OUR_DPI_Trigger_Full_2[c(OUR_row+l),c(4)]<-Non_Control_Batch[l,]

        OUR_DPI_Trigger_Full_2[c(OUR_row+l),c(5)]<-c("OUR_DPI_Trigger_2")

        OUR_DPI_Trigger_Full_2[c(OUR_row+l),c(6)]<-Value_Trigger_2[c(a),]

        OUR_DPI_Trigger_Full_2[c(OUR_row+l),c(7)]<-Derivative_Trigger_2[i,3]
```

```
      }

    }

    #Row numbers for storing trigger data

    DO_row<-nrow(DO_DPI_Trigger_Full_2)

    VCV_row<-nrow(VCV_DPI_Trigger_Full_2)

    OUR_row<-nrow(OUR_DPI_Trigger_Full_2)

  }

}


#### Trigger 3 Calculations ####

Value_Trigger_3<-data.frame(array(c(10,15,20,25,30,35)))

colnames(Value_Trigger_3)<-c("Trigger Value Percent")

DPI_Trigger_3<-1.5

Derivative_Trigger_3<-matrix(c(10, -5e7, -5e-7,

                               15, -1e7, -2e-6,

                               30, -2e8, -1e-6,

                               35, -1.5e8, -4e-7,

                               40, -8e7, -8e-7),nrow=5,ncol=3,byrow=TRUE)

colnames(Derivative_Trigger_3)<-c("DO_Derivative","VCV_Derivative","OUR_Derivative")


DO_DPI_Trigger_Full_3<-as.data.frame(matrix(ncol=4))

colnames(DO_DPI_Trigger_Full_3)<-c("DO_DPI_Trigger","DO%_Trigger","dDO_Trigger","Bat
ch_ID")


VCV_DPI_Trigger_Full_3<-as.data.frame(matrix(ncol=4))
```

```
colnames(VCV_DPI_Trigger_Full_3)<-c("VCV_DPI_Trigger","VCV_Trigger","dVCV_Trigger","
Batch_ID")


OUR_DPI_Trigger_Full_3<-as.data.frame(matrix(ncol=4))

colnames(OUR_DPI_Trigger_Full_3)<-c("OUR_DPI_Trigger","OUR_Trigger","dOUR_Trigger","
Batch_ID")


for (a in 1:nrow(Value_Trigger_3))

{

  for (i in 1:nrow(Derivative_Trigger_3))

  {

    for (l in 1:Num_Batch_ID)

    {

      h<-filter(OUR_Data_Extended,`Batch
ID`==as.character(Non_Control_Batch[l,])&`Infection?`=="INFECTION")

      f<-filter(OUR_Data_Extended,`Batch ID`==as.character(Non_Control_Batch[l,]))


      #setting up empty matrices and filling them with batch data

      DO_Data_Trim<-as.data.frame(matrix(ncol=3))

      colnames(DO_Data_Trim)<-c("DPI","DO%","dDO%")

      DO_Data_Trim[1:nrow(f),]<-f[,(c("DPI","Avg DO hr (%)","dDO (%)"))]


      VCV_Data_Trim<-as.data.frame(matrix(ncol=3))

      colnames(VCV_Data_Trim)<-c("DPI","VCV","dVCV")

      VCV_Data_Trim[1:nrow(f),]<-f[,(c("DPI","Avg VCV hr (um3 cells)","dVCV (um3
cells)"))]
```

```
	OUR_Data_Trim<-as.data.frame(matrix(ncol=3))

	colnames(OUR_Data_Trim)<-c("DPI","OUR","dOUR")

	OUR_Data_Trim[1:nrow(f),]<-f[,(c("DPI","Avg OUR hr (/um3 cells) min","dOUR
(/um3 cells) min" ))]



	#Max and Min DO calculations


Max_DO<-DO_Data_Trim%>%filter(DPI<(-0.5))%>%filter(`DO%`==max(`DO%`,na.rm=TRUE))

	Max_DO<-Max_DO[,c(2)]

	# if (round(Max_DO[,c(1)],0)==(-3))

	# {

	#   Max_DO<-Max_DO[,c(2)]

	# }else

	# {

	#
Max_DO<-DO_Data_Trim%>%filter(DPI>3&`dDO%`<10)%>%filter(`DO%`==max(`DO%`,na.rm=TRUE)
)

	#   Max_DO<-Max_DO[,c(2)]

	#   if (is_empty(Max_DO))

	#   {

	#
Max_DO<-DO_Data_Trim%>%filter(DPI>2)%>%filter(`DO%`==max(`DO%`,na.rm=TRUE))

	#     Max_DO<-Max_DO[,c(2)]

	#   }

	# }


Min_DO<-DO_Data_Trim%>%filter(DPI<(-0.1)&`DO%`>0)%>%filter(`DO%`==min(`DO%`,na.rm=TR
UE))

	Min_DO<-Min_DO[,c(2)]
```

```
#Max and min VCV calculations

Max_VCV<-VCV_Data_Trim%>%filter(DPI<3.5&DPI>0.5)%>%filter(VCV==max(VCV,na.rm=TRUE))

Max_VCV<-Max_VCV[,c(2)]

Min_VCV<-VCV_Data_Trim%>%filter(DPI<(-1))%>%filter(VCV==min(VCV,na.rm=TRUE))

Min_VCV<-Min_VCV[,c(2)]

# if (round(Min_VCV[,c(1)],0)==(-3))

# {

#   Min_VCV<-Min_VCV[,c(2)]

# }else

# {

# Min_VCV<-VCV_Data_Trim%>%filter(DPI>2.5&dVCV>(-1e7)|DPI<(-1))%>%filter(VCV==min(VCV,
na.rm=TRUE))

#   Min_VCV<-Min_VCV[,c(2)]

#   if (is_empty(Min_VCV))

#   {

#     Min_VCV<-VCV_Data_Trim%>%filter()

#   }

# }


#Max and min OUR calculations

Max_OUR<-OUR_Data_Trim%>%filter(DPI<(-0.5))%>%filter(OUR==max(OUR,na.rm=TRUE))

Max_OUR<-Max_OUR[,c(2)]

# if(round(Max_OUR[,c(1)],1)<3&round(Max_OUR[,c(1)],1)>0)
```

```
# {
#   Max_OUR<-Max_OUR[,c(2)]
# }else
# {
#
Max_OUR<-OUR_Data_Trim%>%filter(DPI>0|DPI<(-0.5))%>%filter(DPI<3&dOUR<1e-8)%>%filter
(OUR==max(OUR,na.rm=TRUE))
#   Max_OUR<-Max_OUR[,c(2)]
#   if(is_empty(Max_OUR))
#   {
#     Max_OUR<-OUR_Data_Trim%>%filter()
#   }
# }
Min_OUR<-OUR_Data_Trim%>%filter(DPI<(-1))%>%filter(OUR==min(OUR,na.rm=TRUE))
Min_OUR<-Min_OUR[,c(2)]
# if (round(Min_OUR[,c(1)],0)==(-3))
# {
#   Min_OUR<-Min_OUR[,c(2)]
# }else
# {
#
Min_OUR<-OUR_Data_Trim%>%filter(DPI>2.5&dOUR>(-1e-8)|DPI<(-1))%>%filter(OUR==min(OUR
,na.rm=TRUE))
#   Min_OUR<-Min_OUR[,c(2)]
#   if (is_empty(Min_OUR))
#   {
#     Min_OUR<-OUR_Data_Trim%>%filter()
```

```
      #   }

      # }


      #Filtering for Trigger


Temp_Filter_DO_Full<-DO_Data_Trim%>%filter(DPI>DPI_Trigger_3)%>%filter(`dDO%`<Derivative_Trigger_3[i,1])%>%filter(`DO%`>Max_DO*(1-(Value_Trigger_3[c(a),]/100)))


Temp_Filter_VCV_Full<-VCV_Data_Trim%>%filter(DPI>DPI_Trigger_3)%>%filter(dVCV>Derivative_Trigger_3[i,2])%>%filter(VCV<Min_VCV+(Max_VCV-Min_VCV)*(Value_Trigger_3[c(a),]/100))


Temp_Filter_OUR_Full<-OUR_Data_Trim%>%filter(DPI>DPI_Trigger_3)%>%filter(dOUR>Derivative_Trigger_3[i,3])%>%filter(OUR<Min_OUR+(Max_OUR-Min_OUR)*(Value_Trigger_3[c(a),]/100))




      #Storing Trigger Data

      if (a==1&i==1)

      {

        DO_DPI_Trigger_Full_3[c(l),c(1:3)]<-Temp_Filter_DO_Full[c(1),]

        DO_DPI_Trigger_Full_3[c(l),c(4)]<-Non_Control_Batch[l,]


DO_DPI_Trigger_Full_3<-DO_DPI_Trigger_Full_3%>%mutate(DO_Label="DO_DPI_Trigger_3",`Trigger_Value_%`=Value_Trigger_3[c(a),],`Slope_Value`=Derivative_Trigger_3[i,1])

        DO_row<-nrow(DO_DPI_Trigger_Full_3)



        VCV_DPI_Trigger_Full_3[c(l),c(1:3)]<-Temp_Filter_VCV_Full[c(1),]

        VCV_DPI_Trigger_Full_3[c(l),c(4)]<-Non_Control_Batch[l,]


VCV_DPI_Trigger_Full_3<-VCV_DPI_Trigger_Full_3%>%mutate(VCV_Label="VCV_DPI_Trigger_3
```

```
",`Trigger_Value_%`=Value_Trigger_3[c(a),],`Slope_Value`=Derivative_Trigger_3[i,2])

        VCV_row<-nrow(VCV_DPI_Trigger_Full_3)


        OUR_DPI_Trigger_Full_3[c(l),c(1:3)]<-Temp_Filter_OUR_Full[c(1),]

        OUR_DPI_Trigger_Full_3[c(l),c(4)]<-Non_Control_Batch[l,]

OUR_DPI_Trigger_Full_3<-OUR_DPI_Trigger_Full_3%>%mutate(OUR_Label="OUR_DPI_Trigger_3
",`Trigger_Value_%`=Value_Trigger_3[c(a),],`Slope_Value`=Derivative_Trigger_3[i,3])

        OUR_row<-nrow(OUR_DPI_Trigger_Full_3)


      }else

      {

        DO_DPI_Trigger_Full_3[c(DO_row+l),c(1:3)]<-Temp_Filter_DO_Full[c(1),]

        DO_DPI_Trigger_Full_3[c(DO_row+l),c(4)]<-Non_Control_Batch[l,]

        DO_DPI_Trigger_Full_3[c(DO_row+l),c(5)]<-c("DO_DPI_Trigger_3")

        DO_DPI_Trigger_Full_3[c(DO_row+l),c(6)]<-Value_Trigger_3[c(a),]

        DO_DPI_Trigger_Full_3[c(DO_row+l),c(7)]<-Derivative_Trigger_3[i,1]


        VCV_DPI_Trigger_Full_3[c(VCV_row+l),c(1:3)]<-Temp_Filter_VCV_Full[c(1),]

        VCV_DPI_Trigger_Full_3[c(VCV_row+l),c(4)]<-Non_Control_Batch[l,]

        VCV_DPI_Trigger_Full_3[c(VCV_row+l),c(5)]<-c("VCV_DPI_Trigger_3")

        VCV_DPI_Trigger_Full_3[c(VCV_row+l),c(6)]<-Value_Trigger_3[c(a),]

        VCV_DPI_Trigger_Full_3[c(VCV_row+l),c(7)]<-Derivative_Trigger_3[i,2]


        OUR_DPI_Trigger_Full_3[c(OUR_row+l),c(1:3)]<-Temp_Filter_OUR_Full[c(1),]

        OUR_DPI_Trigger_Full_3[c(OUR_row+l),c(4)]<-Non_Control_Batch[l,]
```

```
        OUR_DPI_Trigger_Full_3[c(OUR_row+l),c(5)]<-c("OUR_DPI_Trigger_3")

        OUR_DPI_Trigger_Full_3[c(OUR_row+l),c(6)]<-Value_Trigger_3[c(a),]

        OUR_DPI_Trigger_Full_3[c(OUR_row+l),c(7)]<-Derivative_Trigger_3[i,3]

      }

    }

    #Row numbers for storing trigger data

    DO_row<-nrow(DO_DPI_Trigger_Full_3)

    VCV_row<-nrow(VCV_DPI_Trigger_Full_3)

    OUR_row<-nrow(OUR_DPI_Trigger_Full_3)

  }

}


DO_DPI_Trigger_Full_1<-merge(DO_DPI_Trigger_Full_1,Peak_VII[,c("Peak_Cont_VII_DPI","Peak_Cont_VII","Batch_ID")],by.x="Batch_ID")%>%mutate(DPI_Offset=Peak_Cont_VII_DPI-DO_DPI_Trigger)

DO_DPI_Trigger_Full_2<-merge(DO_DPI_Trigger_Full_2,Peak_VII[,c("Peak_Cont_VII_DPI","Peak_Cont_VII","Batch_ID")],by.x="Batch_ID")%>%mutate(DPI_Offset=Peak_Cont_VII_DPI-DO_DPI_Trigger)

DO_DPI_Trigger_Full_3<-merge(DO_DPI_Trigger_Full_3,Peak_VII[,c("Peak_Cont_VII_DPI","Peak_Cont_VII","Batch_ID")],by.x="Batch_ID")%>%mutate(DPI_Offset=Peak_Cont_VII_DPI-DO_DPI_Trigger)



VCV_DPI_Trigger_Full_1<-merge(VCV_DPI_Trigger_Full_1,Peak_VII[,c("Peak_Cont_VII_DPI","Peak_Cont_VII","Batch_ID")],by.x="Batch_ID")%>%mutate(DPI_Offset=Peak_Cont_VII_DPI-VCV_DPI_Trigger)

VCV_DPI_Trigger_Full_2<-merge(VCV_DPI_Trigger_Full_2,Peak_VII[,c("Peak_Cont_VII_DPI","Peak_Cont_VII","Batch_ID")],by.x="Batch_ID")%>%mutate(DPI_Offset=Peak_Cont_VII_DPI-VCV_DPI_Trigger)

VCV_DPI_Trigger_Full_3<-merge(VCV_DPI_Trigger_Full_3,Peak_VII[,c("Peak_Cont_VII_DPI","Peak_Cont_VII","Batch_ID")],by.x="Batch_ID")%>%mutate(DPI_Offset=Peak_Cont_VII_DPI-VCV_DPI_Trigger)
```

```
OUR_DPI_Trigger_Full_1<-merge(OUR_DPI_Trigger_Full_1,Peak_VII[,c("Peak_Cont_VII_DPI"
,"Peak_Cont_VII","Batch_ID")],by.x="Batch_ID")%>%mutate(DPI_Offset=Peak_Cont_VII_DPI
-OUR_DPI_Trigger)

OUR_DPI_Trigger_Full_2<-merge(OUR_DPI_Trigger_Full_2,Peak_VII[,c("Peak_Cont_VII_DPI"
,"Peak_Cont_VII","Batch_ID")],by.x="Batch_ID")%>%mutate(DPI_Offset=Peak_Cont_VII_DPI
-OUR_DPI_Trigger)

OUR_DPI_Trigger_Full_3<-merge(OUR_DPI_Trigger_Full_3,Peak_VII[,c("Peak_Cont_VII_DPI"
,"Peak_Cont_VII","Batch_ID")],by.x="Batch_ID")%>%mutate(DPI_Offset=Peak_Cont_VII_DPI
-OUR_DPI_Trigger)




#### Further Analyses ####

DO_Trigger_Summary_1<-group_by(DO_DPI_Trigger_Full_1,`Trigger_Value_%`,`Slope_Value`
)%>%summarize(Mean_Trigger_DPI=mean(DO_DPI_Trigger,na.rm=TRUE),SD_DPI_Trigger=sd(DO_
DPI_Trigger,na.rm=TRUE),Mean_DPI_Offset=mean(DPI_Offset,na.rm=TRUE),SD_DPI_Offset=sd
(DPI_Offset,na.rm=TRUE))%>%mutate(Mean_Peak_VII_DPI=mean(Peak_VII[,c(1)]))

DO_Trigger_Summary_2<-group_by(DO_DPI_Trigger_Full_2,`Trigger_Value_%`,`Slope_Value`
)%>%summarize(Mean_Trigger_DPI=mean(DO_DPI_Trigger,na.rm=TRUE),SD_DPI_Trigger=sd(DO_
DPI_Trigger,na.rm=TRUE),Mean_DPI_Offset=mean(DPI_Offset,na.rm=TRUE),SD_DPI_Offset=sd
(DPI_Offset,na.rm=TRUE))%>%mutate(Mean_Peak_VII_DPI=mean(Peak_VII[,c(1)]))

DO_Trigger_Summary_3<-group_by(DO_DPI_Trigger_Full_3,`Trigger_Value_%`,`Slope_Value`
)%>%summarize(Mean_Trigger_DPI=mean(DO_DPI_Trigger,na.rm=TRUE),SD_DPI_Trigger=sd(DO_
DPI_Trigger,na.rm=TRUE),Mean_DPI_Offset=mean(DPI_Offset,na.rm=TRUE),SD_DPI_Offset=sd
(DPI_Offset,na.rm=TRUE))%>%mutate(Mean_Peak_VII_DPI=mean(Peak_VII[,c(1)]))



VCV_Trigger_Summary_1<-group_by(VCV_DPI_Trigger_Full_1,`Trigger_Value_%`,`Slope_Valu
e`)%>%summarize(Mean_Trigger_DPI=mean(VCV_DPI_Trigger,na.rm=TRUE),SD_DPI_Trigger=sd(
VCV_DPI_Trigger,na.rm=TRUE),Mean_DPI_Offset=mean(DPI_Offset,na.rm=TRUE),SD_DPI_Offse
t=sd(DPI_Offset,na.rm=TRUE))%>%mutate(Mean_Peak_VII_DPI=mean(Peak_VII[,c(1)]))

VCV_Trigger_Summary_2<-group_by(VCV_DPI_Trigger_Full_2,`Trigger_Value_%`,`Slope_Valu
e`)%>%summarize(Mean_Trigger_DPI=mean(VCV_DPI_Trigger,na.rm=TRUE),SD_DPI_Trigger=sd(
VCV_DPI_Trigger,na.rm=TRUE),Mean_DPI_Offset=mean(DPI_Offset,na.rm=TRUE),SD_DPI_Offse
t=sd(DPI_Offset,na.rm=TRUE))%>%mutate(Mean_Peak_VII_DPI=mean(Peak_VII[,c(1)]))

VCV_Trigger_Summary_3<-group_by(VCV_DPI_Trigger_Full_3,`Trigger_Value_%`,`Slope_Valu
e`)%>%summarize(Mean_Trigger_DPI=mean(VCV_DPI_Trigger,na.rm=TRUE),SD_DPI_Trigger=sd(
VCV_DPI_Trigger,na.rm=TRUE),Mean_DPI_Offset=mean(DPI_Offset,na.rm=TRUE),SD_DPI_Offse
```

```
t=sd(DPI_Offset,na.rm=TRUE))%>%mutate(Mean_Peak_VII_DPI=mean(Peak_VII[,c(1)]))


OUR_Trigger_Summary_1<-group_by(OUR_DPI_Trigger_Full_1,`Trigger_Value_%`,`Slope_Valu
e`)%>%summarize(Mean_Trigger_DPI=mean(OUR_DPI_Trigger,na.rm=TRUE),SD_DPI_Trigger=sd(
OUR_DPI_Trigger,na.rm=TRUE),Mean_DPI_Offset=mean(DPI_Offset,na.rm=TRUE),SD_DPI_Offse
t=sd(DPI_Offset,na.rm=TRUE))%>%mutate(Mean_Peak_VII_DPI=mean(Peak_VII[,c(1)]))

OUR_Trigger_Summary_2<-group_by(OUR_DPI_Trigger_Full_2,`Trigger_Value_%`,`Slope_Valu
e`)%>%summarize(Mean_Trigger_DPI=mean(OUR_DPI_Trigger,na.rm=TRUE),SD_DPI_Trigger=sd(
OUR_DPI_Trigger,na.rm=TRUE),Mean_DPI_Offset=mean(DPI_Offset,na.rm=TRUE),SD_DPI_Offse
t=sd(DPI_Offset,na.rm=TRUE))%>%mutate(Mean_Peak_VII_DPI=mean(Peak_VII[,c(1)]))

OUR_Trigger_Summary_3<-group_by(OUR_DPI_Trigger_Full_3,`Trigger_Value_%`,`Slope_Valu
e`)%>%summarize(Mean_Trigger_DPI=mean(OUR_DPI_Trigger,na.rm=TRUE),SD_DPI_Trigger=sd(
OUR_DPI_Trigger,na.rm=TRUE),Mean_DPI_Offset=mean(DPI_Offset,na.rm=TRUE),SD_DPI_Offse
t=sd(DPI_Offset,na.rm=TRUE))%>%mutate(Mean_Peak_VII_DPI=mean(Peak_VII[,c(1)]))


DO_DPI_Trigger_Full_1<-merge(DO_DPI_Trigger_Full_1,DO_Trigger_Summary_1,by.x=c("Trig
ger_Value_%","Slope_Value"))

DO_DPI_Trigger_Full_2<-merge(DO_DPI_Trigger_Full_2,DO_Trigger_Summary_2,by.x=c("Trig
ger_Value_%","Slope_Value"))

DO_DPI_Trigger_Full_3<-merge(DO_DPI_Trigger_Full_3,DO_Trigger_Summary_3,by.x=c("Trig
ger_Value_%","Slope_Value"))


VCV_DPI_Trigger_Full_1<-merge(VCV_DPI_Trigger_Full_1,VCV_Trigger_Summary_1,by.x=c("T
rigger_Value_%","Slope_Value"))

VCV_DPI_Trigger_Full_2<-merge(VCV_DPI_Trigger_Full_2,VCV_Trigger_Summary_2,by.x=c("T
rigger_Value_%","Slope_Value"))

VCV_DPI_Trigger_Full_3<-merge(VCV_DPI_Trigger_Full_3,VCV_Trigger_Summary_3,by.x=c("T
rigger_Value_%","Slope_Value"))


OUR_DPI_Trigger_Full_1<-merge(OUR_DPI_Trigger_Full_1,OUR_Trigger_Summary_1,by.x=c("T
rigger_Value_%","Slope_Value"))

OUR_DPI_Trigger_Full_2<-merge(OUR_DPI_Trigger_Full_2,OUR_Trigger_Summary_2,by.x=c("T
rigger_Value_%","Slope_Value"))

OUR_DPI_Trigger_Full_3<-merge(OUR_DPI_Trigger_Full_3,OUR_Trigger_Summary_3,by.x=c("T
```

```
rigger_Value_%","Slope_Value"))



DO_DPI_Trigger_Total<-rbind(DO_DPI_Trigger_Full_1,DO_DPI_Trigger_Full_2,DO_DPI_Trigg
er_Full_3)%>%mutate(Predicted_Peak_VII_DPI=DO_DPI_Trigger+Mean_DPI_Offset)

VCV_DPI_Trigger_Total<-rbind(VCV_DPI_Trigger_Full_1,VCV_DPI_Trigger_Full_2,VCV_DPI_T
rigger_Full_3)%>%mutate(Predicted_Peak_VII_DPI=VCV_DPI_Trigger+Mean_DPI_Offset)

OUR_DPI_Trigger_Total<-rbind(OUR_DPI_Trigger_Full_1,OUR_DPI_Trigger_Full_2,OUR_DPI_T
rigger_Full_3)%>%mutate(Predicted_Peak_VII_DPI=OUR_DPI_Trigger+Mean_DPI_Offset)



DO_Trigger_Count<-data.frame(matrix(nrow=nrow(DO_DPI_Trigger_Total),ncol=9))

VCV_Trigger_Count<-data.frame(matrix(nrow=nrow(VCV_DPI_Trigger_Total),ncol=9))

OUR_Trigger_Count<-data.frame(matrix(nrow=nrow(OUR_DPI_Trigger_Total),ncol=9))



for (n in 1:nrow(DO_Trigger_Count))

{


Temp_Peak_VII<-as.matrix(Peak_VII%>%filter(Batch_ID==DO_DPI_Trigger_Total[c(n),c("Ba
tch_ID")]))

  for (i in 1:nrow(VII_Percentiles))

  {


Percentile_Range<-as.numeric(Temp_Peak_VII[grep(paste(VII_Percentiles[c(i),],"th","
DPI",sep=""),colnames(Temp_Peak_VII))])

    if (is.na(DO_DPI_Trigger_Total[c(n),c("Predicted_Peak_VII_DPI")]))

    {

      DO_Trigger_Count[c(n),c(i)]<-0

      DO_Trigger_Count[c(n),c(8)]<-NA

      DO_Trigger_Count[c(n),c(9)]<-NA

    }else
```

```
    {

      if
(DO_DPI_Trigger_Total[c(n),c("Predicted_Peak_VII_DPI")]>Percentile_Range[1]&&DO_DPI_
Trigger_Total[c(n),c("Predicted_Peak_VII_DPI")]<Percentile_Range[2])

      {

        DO_Trigger_Count[c(n),c(i)]<-1

        DO_Trigger_Count[c(n),c(8)]<-OUR_Data_Extended%>%filter(`Batch
ID`==DO_DPI_Trigger_Total[c(n),c("Batch_ID")]&DPI<DO_DPI_Trigger_Total[c(n),c("Predi
cted_Peak_VII_DPI")])%>%select(Step_100_row_VII)%>%tail(.,n=1)

        DO_Trigger_Count[c(n),c(9)]<-as.numeric(Temp_Peak_VII[2])

      }else

      {

        DO_Trigger_Count[c(n),c(i)]<-0

        DO_Trigger_Count[c(n),c(8)]<-OUR_Data_Extended%>%filter(`Batch
ID`==DO_DPI_Trigger_Total[c(n),c("Batch_ID")]&DPI<DO_DPI_Trigger_Total[c(n),c("Predi
cted_Peak_VII_DPI")])%>%select(Step_100_row_VII)%>%tail(.,n=1)

        DO_Trigger_Count[c(n),c(9)]<-as.numeric(Temp_Peak_VII[2])

      }

    }

  }

}



for (n in 1:nrow(VCV_Trigger_Count))

{


Temp_Peak_VII<-as.matrix(Peak_VII%>%filter(Batch_ID==VCV_DPI_Trigger_Total[c(n),c("B
atch_ID")]))

  for (i in 1:nrow(VII_Percentiles))

  {
```

```
Percentile_Range<-as.numeric(Temp_Peak_VII[grep(paste(VII_Percentiles[c(i),],"th","
DPI",sep=""),colnames(Temp_Peak_VII))])

    if (is.na(VCV_DPI_Trigger_Total[c(n),c("Predicted_Peak_VII_DPI")]))

    {

      VCV_Trigger_Count[c(n),c(i)]<-0

      VCV_Trigger_Count[c(n),c(8)]<-NA

      VCV_Trigger_Count[c(n),c(9)]<-NA

    }else

    {

      if
(VCV_DPI_Trigger_Total[c(n),c("Predicted_Peak_VII_DPI")]>Percentile_Range[1]&&VCV_DP
I_Trigger_Total[c(n),c("Predicted_Peak_VII_DPI")]<Percentile_Range[2])

      {

        VCV_Trigger_Count[c(n),c(i)]<-1

        VCV_Trigger_Count[c(n),c(8)]<-OUR_Data_Extended%>%filter(`Batch
ID`==VCV_DPI_Trigger_Total[c(n),c("Batch_ID")]&DPI<VCV_DPI_Trigger_Total[c(n),c("Pre
dicted_Peak_VII_DPI")])%>%select(Step_100_row_VII)%>%tail(.,n=1)

        VCV_Trigger_Count[c(n),c(9)]<-as.numeric(Temp_Peak_VII[2])

      }else

      {

        VCV_Trigger_Count[c(n),c(i)]<-0

        VCV_Trigger_Count[c(n),c(8)]<-OUR_Data_Extended%>%filter(`Batch
ID`==VCV_DPI_Trigger_Total[c(n),c("Batch_ID")]&DPI<VCV_DPI_Trigger_Total[c(n),c("Pre
dicted_Peak_VII_DPI")])%>%select(Step_100_row_VII)%>%tail(.,n=1)

        VCV_Trigger_Count[c(n),c(9)]<-as.numeric(Temp_Peak_VII[2])

      }

    }

  }
```

```
}

for (n in 1:nrow(OUR_Trigger_Count))
{

Temp_Peak_VII<-as.matrix(Peak_VII%>%filter(Batch_ID==OUR_DPI_Trigger_Total[c(n),c("B
atch_ID")]))
  for (i in 1:nrow(VII_Percentiles))
  {

Percentile_Range<-as.numeric(Temp_Peak_VII[grep(paste(VII_Percentiles[c(i),],"th","
DPI",sep=""),colnames(Temp_Peak_VII))])
    if (is.na(OUR_DPI_Trigger_Total[c(n),c("Predicted_Peak_VII_DPI")]))
    {
      OUR_Trigger_Count[c(n),c(i)]<-0
      OUR_Trigger_Count[c(n),c(8)]<-NA
      OUR_Trigger_Count[c(n),c(9)]<-NA
    }else
    {
      if
(OUR_DPI_Trigger_Total[c(n),c("Predicted_Peak_VII_DPI")]>Percentile_Range[1]&&OUR_DP
I_Trigger_Total[c(n),c("Predicted_Peak_VII_DPI")]<Percentile_Range[2])
      {
        OUR_Trigger_Count[c(n),c(i)]<-1
        OUR_Trigger_Count[c(n),c(8)]<-OUR_Data_Extended%>%filter(`Batch
ID`==OUR_DPI_Trigger_Total[c(n),c("Batch_ID")]&DPI<OUR_DPI_Trigger_Total[c(n),c("Pre
dicted_Peak_VII_DPI")])%>%select(Step_100_row_VII)%>%tail(.,n=1)
        OUR_Trigger_Count[c(n),c(9)]<-as.numeric(Temp_Peak_VII[2])
      }else
```

```
      {

        OUR_Trigger_Count[c(n),c(i)]<-0

        OUR_Trigger_Count[c(n),c(8)]<-OUR_Data_Extended%>%filter(`Batch ID`==OUR_DPI_Trigger_Total[c(n),c("Batch_ID")]&DPI<OUR_DPI_Trigger_Total[c(n),c("Predicted_Peak_VII_DPI")])%>%select(Step_100_row_VII)%>%tail(.,n=1)

        OUR_Trigger_Count[c(n),c(9)]<-as.numeric(Temp_Peak_VII[2])

      }

    }

  }

}



colnames(DO_Trigger_Count)<-c(t(VII_Percentiles),"Predicted_Peak_VII","Peak_Cont_VII")

colnames(VCV_Trigger_Count)<-c(t(VII_Percentiles),"Predicted_Peak_VII","Peak_Cont_VII")

colnames(OUR_Trigger_Count)<-c(t(VII_Percentiles),"Predicted_Peak_VII","Peak_Cont_VII")



DO_Trigger_Count<-DO_Trigger_Count%>%mutate(Percent_Peak_Cont_VII=(Predicted_Peak_VII/Peak_Cont_VII)*100)

VCV_Trigger_Count<-VCV_Trigger_Count%>%mutate(Percent_Peak_Cont_VII=(Predicted_Peak_VII/Peak_Cont_VII)*100)

OUR_Trigger_Count<-OUR_Trigger_Count%>%mutate(Percent_Peak_Cont_VII=(Predicted_Peak_VII/Peak_Cont_VII)*100)




DO_Trigger_Count_Summary<-cbind(DO_DPI_Trigger_Total[,c(1,2,7)],DO_Trigger_Count)%>%


mutate(Identifier=paste(paste(`Trigger_Value_%`,"%",sep=""),`Slope_Value`,DO_Label,sep="_"))%>%
```

```
  group_by(Identifier)%>%

summarize(`95_Sum`=sum(`95`,na.rm=TRUE),`90_Sum`=sum(`90`,na.rm=TRUE),`85_Sum`=sum(`
85`,na.rm=TRUE),`80_Sum`=sum(`80`,na.rm=TRUE),`75_Sum`=sum(`75`,na.rm=TRUE),`70_Sum`
=sum(`70`,na.rm=TRUE),`65_Sum`=sum(`65`,na.rm=TRUE),`Average_%_Peak_VII`=mean(Percen
t_Peak_Cont_VII,na.rm=TRUE),`SD_%_Peak_VII`=sd(Percent_Peak_Cont_VII,na.rm=TRUE))%>%

mutate(`95%`=(`95_Sum`/nrow(Non_Control_Batch))*100,`90%`=(`90_Sum`/nrow(Non_Control
_Batch))*100,`85%`=(`85_Sum`/nrow(Non_Control_Batch))*100,`80%`=(`80_Sum`/nrow(Non_C
ontrol_Batch))*100,`75%`=(`75_Sum`/nrow(Non_Control_Batch))*100,`70%`=(`70_Sum`/nrow
(Non_Control_Batch))*100,`65%`=(`65_Sum`/nrow(Non_Control_Batch))*100)

VCV_Trigger_Count_Summary<-cbind(VCV_DPI_Trigger_Total[,c(1,2,7)],VCV_Trigger_Count)
%>%

mutate(Identifier=paste(paste(`Trigger_Value_%`,"%",sep=""),`Slope_Value`,VCV_Label,
sep="_"))%>%

  group_by(Identifier)%>%

summarize(`95_Sum`=sum(`95`,na.rm=TRUE),`90_Sum`=sum(`90`,na.rm=TRUE),`85_Sum`=sum(`
85`,na.rm=TRUE),`80_Sum`=sum(`80`,na.rm=TRUE),`75_Sum`=sum(`75`,na.rm=TRUE),`70_Sum`
=sum(`70`,na.rm=TRUE),`65_Sum`=sum(`65`,na.rm=TRUE),`Average_%_Peak_VII`=mean(Percen
t_Peak_Cont_VII,na.rm=TRUE),`SD_%_Peak_VII`=sd(Percent_Peak_Cont_VII,na.rm=TRUE))%>%

mutate(`95%`=(`95_Sum`/nrow(Non_Control_Batch))*100,`90%`=(`90_Sum`/nrow(Non_Control
_Batch))*100,`85%`=(`85_Sum`/nrow(Non_Control_Batch))*100,`80%`=(`80_Sum`/nrow(Non_C
ontrol_Batch))*100,`75%`=(`75_Sum`/nrow(Non_Control_Batch))*100,`70%`=(`70_Sum`/nrow
(Non_Control_Batch))*100,`65%`=(`65_Sum`/nrow(Non_Control_Batch))*100)

OUR_Trigger_Count_Summary<-cbind(OUR_DPI_Trigger_Total[,c(1,2,7)],OUR_Trigger_Count)
%>%

mutate(Identifier=paste(paste(`Trigger_Value_%`,"%",sep=""),`Slope_Value`,OUR_Label,
sep="_"))%>%

  group_by(Identifier)%>%

summarize(`95_Sum`=sum(`95`,na.rm=TRUE),`90_Sum`=sum(`90`,na.rm=TRUE),`85_Sum`=sum(`
85`,na.rm=TRUE),`80_Sum`=sum(`80`,na.rm=TRUE),`75_Sum`=sum(`75`,na.rm=TRUE),`70_Sum`
=sum(`70`,na.rm=TRUE),`65_Sum`=sum(`65`,na.rm=TRUE),`Average_%_Peak_VII`=mean(Percen
t_Peak_Cont_VII,na.rm=TRUE),`SD_%_Peak_VII`=sd(Percent_Peak_Cont_VII,na.rm=TRUE))%>%
```

```
mutate(`95%`=(`95_Sum`/nrow(Non_Control_Batch))*100,`90%`=(`90_Sum`/nrow(Non_Control
_Batch))*100,`85%`=(`85_Sum`/nrow(Non_Control_Batch))*100,`80%`=(`80_Sum`/nrow(Non_C
ontrol_Batch))*100,`75%`=(`75_Sum`/nrow(Non_Control_Batch))*100,`70%`=(`70_Sum`/nrow
(Non_Control_Batch))*100,`65%`=(`65_Sum`/nrow(Non_Control_Batch))*100)



DO_DPI_Trigger_Total<-cbind(DO_DPI_Trigger_Total,DO_Trigger_Count[,c(8,10)])

VCV_DPI_Trigger_Total<-cbind(VCV_DPI_Trigger_Total,VCV_Trigger_Count[,c(8,10)])

OUR_DPI_Trigger_Total<-cbind(OUR_DPI_Trigger_Total,OUR_Trigger_Count[,c(8,10)])



Summary_List<-list(DO_Trigger_Count_Summary,VCV_Trigger_Count_Summary,OUR_Trigger_Co
unt_Summary)

Summary_Names<-c("DO_Trigger_Count_Summary","VCV_Trigger_Count_Summary","OUR_Trigger
_Count_Summary")

names(Summary_List)<-Summary_Names

write_xlsx(Summary_List,"Trigger_Count_Summary.xlsx")



Total_List<-list(DO_DPI_Trigger_Total,VCV_DPI_Trigger_Total,OUR_DPI_Trigger_Total)

Total_Names<-c("DO_DPI_Trigger_Total","VCV_DPI_Trigger_Total","OUR_DPI_Trigger_Total
")

names(Total_List)<-Total_Names

write_xlsx(Total_List,"Trigger_Data_Total.xlsx")
```
