# Supplementary material for: Minimizing batch‐to‐batch variability of a live virus vaccine by process analytical technologies
Source: Biotechnol Prog. 2025 May 22;41(5):e70037. doi: 10.1002/btpr.70037 (PMC12531925; doi:10.1002/btpr.70037)
Supplement: Supplementary file 3 — Data S3. Supporting Information. [file BTPR-41-e70037-s002.pdf]

```
rm(list=ls())


library(rstudioapi)

library(readxl)

library(tidyverse)

library(dplyr)

library(janitor)

library(lubridate)

library(readr)

library(writexl)

library(openxlsx)

library(stringr)

library(tidyr)

library(tibble)

library(ggplot2)

library(ggpubr)


File_Name <- selectFile()

OUR_Data <- read_excel(File_Name,sheet="Sheet1",guess_max=10000)

Batch_IDs<-unique(OUR_Data[,c(3)])

Num_Batch_ID<-nrow(Batch_IDs)

OUR_Data[,"VII"]<-as.numeric(unlist(OUR_Data[,"VII"]))

OUR_Data[,"Qp"]<-as.numeric(unlist(OUR_Data[,"Qp"]))

OUR_Data[which(OUR_Data[,"Infection?"]=="INFECTION"&OUR_Data[,"VII"]<2e5&OUR_Data[,"
VII"]>0),"Qp"]<-0
```

```
i<-1

for (i in 1:Num_Batch_ID)

  {

    Filtered_OUR_Data<-OUR_Data%>%filter(`Batch ID` %in% Batch_IDs[i,] & `Qp` !=
"NA")

    n<-nrow(Filtered_OUR_Data)

    j<-1

    for (j in 1:n)

    {

      Temp_OUR_Data<-OUR_Data%>%filter(`Batch ID` %in% Batch_IDs[i,])

      if (j==n)

      {

        Temp_OUR_Data<-filter(Temp_OUR_Data,
DPP==as.numeric(Filtered_OUR_Data[j,'DPP']))

        m<-nrow(Temp_OUR_Data)

        Step_Qp<-data.frame(matrix(nrow=m,ncol=1))

        Step_Qp[1,]<-Filtered_OUR_Data[j,'Qp']

        Linear_Qp<-Filtered_OUR_Data[j,'Qp']


Shift_Linear_Qp<-Linear_Qp+mean(as.array(unlist(Filtered_OUR_Data[(j-1):j,'Qp'])))

      } else

      {

        Temp_OUR_Data<-filter(Temp_OUR_Data,
DPP>=as.numeric(Filtered_OUR_Data[j,'DPP']) &
DPP<as.numeric(Filtered_OUR_Data[j+1,'DPP']))

        m<-nrow(Temp_OUR_Data)
```

```
        Step_Qp<-data.frame(matrix(nrow=m,ncol=1))

        Step_Qp[1,]<-Filtered_OUR_Data[j,'Qp']

        Step_Qp[2:m,]<-Filtered_OUR_Data[j+1,'Qp']

Linear_Data<-approx(as.array(unlist(Filtered_OUR_Data[j:(j+1),'DPP'])),as.array(unli
st(Filtered_OUR_Data[j:(j+1),'Qp'])),as.array(unlist(Temp_OUR_Data[1:m,'DPP'])))

        Linear_Qp<-as.data.frame(Linear_Data[["y"]])

        Shift_Linear_Qp<-data.frame(matrix(nrow=nrow(Linear_Qp)),ncol=1)

Shift_Linear_Qp<-Linear_Qp+mean(as.array(unlist(Filtered_OUR_Data[j:(j+1),'Qp'])))

      }

      if (j==1)

        {

          Temp_Interpolated_Data=data.frame(matrix(nrow=m,ncol=ncol(Temp_OUR_Data)))

          Temp_Interpolated_Data[j:m,]<-Temp_OUR_Data

          Temp_Interpolated_Data<-Temp_Interpolated_Data%>%add_column(Step_Qp=NA,
Linear_Qp=NA, Shift_Linear_Qp=NA)

          Temp_Interpolated_Data[(j):(m),c(ncol(Temp_OUR_Data)+1)]<-Step_Qp[1:m,]

          Temp_Interpolated_Data[(j):(m),c(ncol(Temp_OUR_Data)+2)]<-Linear_Qp

          Temp_Interpolated_Data[(j):(m),c(ncol(Temp_OUR_Data)+3)]<-Shift_Linear_Qp

colnames(Temp_Interpolated_Data)<-c(colnames(Temp_OUR_Data),"Step_Qp","Linear_Qp","S
hift_Linear_Qp")

          q<-nrow(Temp_Interpolated_Data)

        } else

        {

Temp_Interpolated_Data[(q+1):(q+m),c(1:ncol(Temp_OUR_Data))]<-Temp_OUR_Data
```

```
Temp_Interpolated_Data[(q+1):(q+m),c(ncol(Temp_OUR_Data)+1)]<-Step_Qp[1:m,]

          Temp_Interpolated_Data[(q+1):(q+m),c(ncol(Temp_OUR_Data)+2)]<-Linear_Qp

Temp_Interpolated_Data[(q+1):(q+m),c(ncol(Temp_OUR_Data)+3)]<-Shift_Linear_Qp

          q<-nrow(Temp_Interpolated_Data)

        }

      j<-j+1

    }

    if (i==1)

      {

Interpolated_Data<-data.frame(matrix(nrow=nrow(Temp_Interpolated_Data),ncol=ncol(Tem
p_Interpolated_Data)))

        Interpolated_Data[i:nrow(Temp_Interpolated_Data),]<-Temp_Interpolated_Data

        colnames(Interpolated_Data)<-c(colnames(Temp_Interpolated_Data))

        k<-nrow(Interpolated_Data)

      } else

      {

Interpolated_Data[(k+1):(k+nrow(Temp_Interpolated_Data)),]<-Temp_Interpolated_Data

        k<-nrow(Interpolated_Data)

      }

    i<-i+1

  }


Death_Rate<-0.36
```

```
Column_Names<-c('Step_min_VII','Step_hr_VII','Step_min_avg_hr_VII','Step_50_row_VII'
,'Step_min_avg_2hr_VII','Step_100_row_VII','Step_200_row_VII','Step_600_row_VII','St
ep_linearized_hr_VII','Step_linearized_2hr_VII','Step_linearized_3hr_VII','Linear_mi
n_VII','Linear_hr_VII','Linear_min_avg_hr_VII','Linear_50_row_VII','Linear_min_avg_2
hr_VII','Linear_100_row_VII','Linear_200_row_VII','Linear_600_row_VII','Linear_linea
rized_hr_VII','Linear_linearized_2hr_VII','Linear_linearized_3hr_VII','Shift_Linear_
min_VII','Shift_Linear_hr_VII','Shift_Linear_min_avg_hr_VII','Shift_Linear_50_row_VI
I','Shift_Linear_min_avg_2hr_VII','Shift_Linear_100_row_VII','Shift_Linear_200_row_V
II','Shift_Linear_600_row_VII','Shift_Linear_linearized_hr_VII','Shift_Linear_linear
ized_2hr_VII','Shift_Linear_linearized_3hr_VII')

for (nm in Column_Names) Interpolated_Data<-Interpolated_Data%>%mutate(!! nm:=NA)

p<-1

r<-1

Linear_VII<-data.frame(matrix(nrow=nrow(Interpolated_Data),ncol=33))



for (r in 1:Num_Batch_ID)

  {

    Temp_Data<-Interpolated_Data%>%filter(`Batch ID` %in% Batch_IDs[r,])

    c<-nrow(Temp_Data)

    Temp_Linear_VII<-data.frame(matrix(nrow=c,ncol=33))

    for (p in 1:c)

    {

      if (p==1) #is.na(Temp_Data[p,'VII'])-->enter this into the if statement for
forced VII and switch the contents of the conditional statements

      {

        Temp_Linear_VII[p,1]<-as.numeric(Temp_Data[p,'VII'])

        Temp_Linear_VII[p,2]<-as.numeric(Temp_Data[p,'VII'])

        Temp_Linear_VII[p,3]<-as.numeric(Temp_Data[p,'VII'])

        Temp_Linear_VII[p,4]<-as.numeric(Temp_Data[p,'VII'])

        Temp_Linear_VII[p,5]<-as.numeric(Temp_Data[p,'VII'])
```

```
Temp_Linear_VII[p,6]<-as.numeric(Temp_Data[p,'VII'])

Temp_Linear_VII[p,7]<-as.numeric(Temp_Data[p,'VII'])

Temp_Linear_VII[p,8]<-as.numeric(Temp_Data[p,'VII'])

Temp_Linear_VII[p,9]<-as.numeric(Temp_Data[p,'VII'])

Temp_Linear_VII[p,10]<-as.numeric(Temp_Data[p,'VII'])

Temp_Linear_VII[p,11]<-as.numeric(Temp_Data[p,'VII'])

Temp_Linear_VII[p,12]<-as.numeric(Temp_Data[p,'VII'])

Temp_Linear_VII[p,13]<-as.numeric(Temp_Data[p,'VII'])

Temp_Linear_VII[p,14]<-as.numeric(Temp_Data[p,'VII'])

Temp_Linear_VII[p,15]<-as.numeric(Temp_Data[p,'VII'])

Temp_Linear_VII[p,16]<-as.numeric(Temp_Data[p,'VII'])

Temp_Linear_VII[p,17]<-as.numeric(Temp_Data[p,'VII'])

Temp_Linear_VII[p,18]<-as.numeric(Temp_Data[p,'VII'])

Temp_Linear_VII[p,19]<-as.numeric(Temp_Data[p,'VII'])

Temp_Linear_VII[p,20]<-as.numeric(Temp_Data[p,'VII'])

Temp_Linear_VII[p,21]<-as.numeric(Temp_Data[p,'VII'])

Temp_Linear_VII[p,22]<-as.numeric(Temp_Data[p,'VII'])

Temp_Linear_VII[p,23]<-as.numeric(Temp_Data[p,'VII'])

Temp_Linear_VII[p,24]<-as.numeric(Temp_Data[p,'VII'])

Temp_Linear_VII[p,25]<-as.numeric(Temp_Data[p,'VII'])

Temp_Linear_VII[p,26]<-as.numeric(Temp_Data[p,'VII'])

Temp_Linear_VII[p,27]<-as.numeric(Temp_Data[p,'VII'])

Temp_Linear_VII[p,28]<-as.numeric(Temp_Data[p,'VII'])

Temp_Linear_VII[p,29]<-as.numeric(Temp_Data[p,'VII'])

Temp_Linear_VII[p,30]<-as.numeric(Temp_Data[p,'VII'])
```

```
          Temp_Linear_VII[p,31]<-as.numeric(Temp_Data[p,'VII'])

          Temp_Linear_VII[p,32]<-as.numeric(Temp_Data[p,'VII'])

          Temp_Linear_VII[p,33]<-as.numeric(Temp_Data[p,'VII'])


        } else

        {

          if (is.na(Temp_Data[p,"Growth Rate (600)"]))

          {

            Temp_Data[p,"Growth Rate (600)"]<-Temp_Data[p-1,"Growth Rate (600)"]

          }

Temp_Linear_VII[p,1]<-((as.numeric(Temp_Data[p,"Step_Qp"])*as.numeric(Temp_Data[p-1,
'VCV'])*(exp(as.numeric(Temp_Data[p,"Growth Rate
(min)"])*(as.numeric(Temp_Data[p,'DPP'])-as.numeric(Temp_Data[p-1,'DPP'])))-exp(-Dea
th_Rate*(as.numeric(Temp_Data[p,'DPP'])-as.numeric(Temp_Data[p-1,'DPP'])))))/(Death_
Rate+as.numeric(Temp_Data[p,"Growth Rate
(min)"])))+(as.numeric(Temp_Linear_VII[p-1,1])*exp(-Death_Rate*(as.numeric(Temp_Data
[p,'DPP'])-as.numeric(Temp_Data[p-1,'DPP']))))


Temp_Linear_VII[p,2]<-((as.numeric(Temp_Data[p,"Step_Qp"])*as.numeric(Temp_Data[p-1,
'VCV'])*(exp(as.numeric(Temp_Data[p,"Growth Rate
(hr)"])*(as.numeric(Temp_Data[p,'DPP'])-as.numeric(Temp_Data[p-1,'DPP'])))-exp(-Deat
h_Rate*(as.numeric(Temp_Data[p,'DPP'])-as.numeric(Temp_Data[p-1,'DPP'])))))/(Death_R
ate+as.numeric(Temp_Data[p,"Growth Rate
(hr)"])))+(as.numeric(Temp_Linear_VII[p-1,2])*exp(-Death_Rate*(as.numeric(Temp_Data[
p,'DPP'])-as.numeric(Temp_Data[p-1,'DPP']))))


Temp_Linear_VII[p,3]<-((as.numeric(Temp_Data[p,"Step_Qp"])*as.numeric(Temp_Data[p-1,
'VCV'])*(exp(as.numeric(Temp_Data[p,"Growth Rate (min avg
hr)"])*(as.numeric(Temp_Data[p,'DPP'])-as.numeric(Temp_Data[p-1,'DPP'])))-exp(-Death
_Rate*(as.numeric(Temp_Data[p,'DPP'])-as.numeric(Temp_Data[p-1,'DPP'])))))/(Death_Ra
te+as.numeric(Temp_Data[p,"Growth Rate (min avg
hr)"])))+(as.numeric(Temp_Linear_VII[p-1,3])*exp(-Death_Rate*(as.numeric(Temp_Data[p
,'DPP'])-as.numeric(Temp_Data[p-1,'DPP']))))


Temp_Linear_VII[p,4]<-((as.numeric(Temp_Data[p,"Step_Qp"])*as.numeric(Temp_Data[p-1,
'VCV'])*(exp(as.numeric(Temp_Data[p,"Growth Rate
```

```
(50)"])*(as.numeric(Temp_Data[p,'DPP'])-as.numeric(Temp_Data[p-1,'DPP'])))-exp(-Deat
h_Rate*(as.numeric(Temp_Data[p,'DPP'])-as.numeric(Temp_Data[p-1,'DPP'])))))/(Death_R
ate+as.numeric(Temp_Data[p,"Growth Rate
(50)"])))+(as.numeric(Temp_Linear_VII[p-1,4])*exp(-Death_Rate*(as.numeric(Temp_Data[
p,'DPP'])-as.numeric(Temp_Data[p-1,'DPP']))))

Temp_Linear_VII[p,5]<-((as.numeric(Temp_Data[p,"Step_Qp"])*as.numeric(Temp_Data[p-1,
'VCV'])*(exp(as.numeric(Temp_Data[p,"Growth Rate (min avg
2hr)"])*(as.numeric(Temp_Data[p,'DPP'])-as.numeric(Temp_Data[p-1,'DPP'])))-exp(-Deat
h_Rate*(as.numeric(Temp_Data[p,'DPP'])-as.numeric(Temp_Data[p-1,'DPP'])))))/(Death_R
ate+as.numeric(Temp_Data[p,"Growth Rate (min avg
2hr)"])))+(as.numeric(Temp_Linear_VII[p-1,5])*exp(-Death_Rate*(as.numeric(Temp_Data[
p,'DPP'])-as.numeric(Temp_Data[p-1,'DPP']))))

Temp_Linear_VII[p,6]<-((as.numeric(Temp_Data[p,"Step_Qp"])*as.numeric(Temp_Data[p-1,
'VCV'])*(exp(as.numeric(Temp_Data[p,"Growth Rate
(100)"])*(as.numeric(Temp_Data[p,'DPP'])-as.numeric(Temp_Data[p-1,'DPP'])))-exp(-Dea
th_Rate*(as.numeric(Temp_Data[p,'DPP'])-as.numeric(Temp_Data[p-1,'DPP'])))))/(Death_
Rate+as.numeric(Temp_Data[p,"Growth Rate
(100)"])))+(as.numeric(Temp_Linear_VII[p-1,6])*exp(-Death_Rate*(as.numeric(Temp_Data
[p,'DPP'])-as.numeric(Temp_Data[p-1,'DPP']))))

Temp_Linear_VII[p,7]<-((as.numeric(Temp_Data[p,"Step_Qp"])*as.numeric(Temp_Data[p-1,
'VCV'])*(exp(as.numeric(Temp_Data[p,"Growth Rate
(200)"])*(as.numeric(Temp_Data[p,'DPP'])-as.numeric(Temp_Data[p-1,'DPP'])))-exp(-Dea
th_Rate*(as.numeric(Temp_Data[p,'DPP'])-as.numeric(Temp_Data[p-1,'DPP'])))))/(Death_
Rate+as.numeric(Temp_Data[p,"Growth Rate
(200)"])))+(as.numeric(Temp_Linear_VII[p-1,7])*exp(-Death_Rate*(as.numeric(Temp_Data
[p,'DPP'])-as.numeric(Temp_Data[p-1,'DPP']))))

Temp_Linear_VII[p,8]<-((as.numeric(Temp_Data[p,"Step_Qp"])*as.numeric(Temp_Data[p-1,
'VCV'])*(exp(as.numeric(Temp_Data[p,"Growth Rate
(600)"])*(as.numeric(Temp_Data[p,'DPP'])-as.numeric(Temp_Data[p-1,'DPP'])))-exp(-Dea
th_Rate*(as.numeric(Temp_Data[p,'DPP'])-as.numeric(Temp_Data[p-1,'DPP'])))))/(Death_
Rate+as.numeric(Temp_Data[p,"Growth Rate
(600)"])))+(as.numeric(Temp_Linear_VII[p-1,8])*exp(-Death_Rate*(as.numeric(Temp_Data
[p,'DPP'])-as.numeric(Temp_Data[p-1,'DPP']))))

Temp_Linear_VII[p,9]<-((as.numeric(Temp_Data[p,"Step_Qp"])*as.numeric(Temp_Data[p-1,
'VCV'])*(exp(as.numeric(Temp_Data[p,"Growth Rate (linearized
hr)"])*(as.numeric(Temp_Data[p,'DPP'])-as.numeric(Temp_Data[p-1,'DPP'])))-exp(-Death
_Rate*(as.numeric(Temp_Data[p,'DPP'])-as.numeric(Temp_Data[p-1,'DPP'])))))/(Death_Ra
te+as.numeric(Temp_Data[p,"Growth Rate (linearized
hr)"])))+(as.numeric(Temp_Linear_VII[p-1,9])*exp(-Death_Rate*(as.numeric(Temp_Data[p
,'DPP'])-as.numeric(Temp_Data[p-1,'DPP']))))
```

```
Temp_Linear_VII[p,10]<-((as.numeric(Temp_Data[p,"Step_Qp"])*as.numeric(Temp_Data[p-1
,'VCV'])*(exp(as.numeric(Temp_Data[p,"Growth Rate (linearized
2hr)"])*(as.numeric(Temp_Data[p,'DPP'])-as.numeric(Temp_Data[p-1,'DPP'])))-exp(-Deat
h_Rate*(as.numeric(Temp_Data[p,'DPP'])-as.numeric(Temp_Data[p-1,'DPP'])))))/(Death_R
ate+as.numeric(Temp_Data[p,"Growth Rate (linearized
2hr)"])))+(as.numeric(Temp_Linear_VII[p-1,10])*exp(-Death_Rate*(as.numeric(Temp_Data
[p,'DPP'])-as.numeric(Temp_Data[p-1,'DPP']))))

Temp_Linear_VII[p,11]<-((as.numeric(Temp_Data[p,"Step_Qp"])*as.numeric(Temp_Data[p-1
,'VCV'])*(exp(as.numeric(Temp_Data[p,"Growth Rate (linearized
3hr)"])*(as.numeric(Temp_Data[p,'DPP'])-as.numeric(Temp_Data[p-1,'DPP'])))-exp(-Deat
h_Rate*(as.numeric(Temp_Data[p,'DPP'])-as.numeric(Temp_Data[p-1,'DPP'])))))/(Death_R
ate+as.numeric(Temp_Data[p,"Growth Rate (linearized
3hr)"])))+(as.numeric(Temp_Linear_VII[p-1,11])*exp(-Death_Rate*(as.numeric(Temp_Data
[p,'DPP'])-as.numeric(Temp_Data[p-1,'DPP']))))

Temp_Linear_VII[p,12]<-((as.numeric(Temp_Data[p,"Linear_Qp"])*as.numeric(Temp_Data[p
-1,'VCV'])*(exp(as.numeric(Temp_Data[p,"Growth Rate
(min)"])*(as.numeric(Temp_Data[p,'DPP'])-as.numeric(Temp_Data[p-1,'DPP'])))-exp(-Dea
th_Rate*(as.numeric(Temp_Data[p,'DPP'])-as.numeric(Temp_Data[p-1,'DPP'])))))/(Death_
Rate+as.numeric(Temp_Data[p,"Growth Rate
(min)"])))+(as.numeric(Temp_Linear_VII[p-1,12])*exp(-Death_Rate*(as.numeric(Temp_Dat
a[p,'DPP'])-as.numeric(Temp_Data[p-1,'DPP']))))

Temp_Linear_VII[p,13]<-((as.numeric(Temp_Data[p,"Linear_Qp"])*as.numeric(Temp_Data[p
-1,'VCV'])*(exp(as.numeric(Temp_Data[p,"Growth Rate
(hr)"])*(as.numeric(Temp_Data[p,'DPP'])-as.numeric(Temp_Data[p-1,'DPP'])))-exp(-Deat
h_Rate*(as.numeric(Temp_Data[p,'DPP'])-as.numeric(Temp_Data[p-1,'DPP'])))))/(Death_R
ate+as.numeric(Temp_Data[p,"Growth Rate
(hr)"])))+(as.numeric(Temp_Linear_VII[p-1,13])*exp(-Death_Rate*(as.numeric(Temp_Data
[p,'DPP'])-as.numeric(Temp_Data[p-1,'DPP']))))

Temp_Linear_VII[p,14]<-((as.numeric(Temp_Data[p,"Linear_Qp"])*as.numeric(Temp_Data[p
-1,'VCV'])*(exp(as.numeric(Temp_Data[p,"Growth Rate (min avg
hr)"])*(as.numeric(Temp_Data[p,'DPP'])-as.numeric(Temp_Data[p-1,'DPP'])))-exp(-Death
_Rate*(as.numeric(Temp_Data[p,'DPP'])-as.numeric(Temp_Data[p-1,'DPP'])))))/(Death_Ra
te+as.numeric(Temp_Data[p,"Growth Rate (min avg
hr)"])))+(as.numeric(Temp_Linear_VII[p-1,14])*exp(-Death_Rate*(as.numeric(Temp_Data[
p,'DPP'])-as.numeric(Temp_Data[p-1,'DPP']))))

Temp_Linear_VII[p,15]<-((as.numeric(Temp_Data[p,"Linear_Qp"])*as.numeric(Temp_Data[p
```

```
-1,'VCV'])*(exp(as.numeric(Temp_Data[p,"Growth Rate
(50)"])*(as.numeric(Temp_Data[p,'DPP'])-as.numeric(Temp_Data[p-1,'DPP'])))-exp(-Deat
h_Rate*(as.numeric(Temp_Data[p,'DPP'])-as.numeric(Temp_Data[p-1,'DPP'])))))/(Death_R
ate+as.numeric(Temp_Data[p,"Growth Rate
(50)"])))+(as.numeric(Temp_Linear_VII[p-1,15])*exp(-Death_Rate*(as.numeric(Temp_Data
[p,'DPP'])-as.numeric(Temp_Data[p-1,'DPP']))))


Temp_Linear_VII[p,16]<-((as.numeric(Temp_Data[p,"Linear_Qp"])*as.numeric(Temp_Data[p
-1,'VCV'])*(exp(as.numeric(Temp_Data[p,"Growth Rate (min avg
2hr)"])*(as.numeric(Temp_Data[p,'DPP'])-as.numeric(Temp_Data[p-1,'DPP'])))-exp(-Deat
h_Rate*(as.numeric(Temp_Data[p,'DPP'])-as.numeric(Temp_Data[p-1,'DPP'])))))/(Death_R
ate+as.numeric(Temp_Data[p,"Growth Rate (min avg
2hr)"])))+(as.numeric(Temp_Linear_VII[p-1,16])*exp(-Death_Rate*(as.numeric(Temp_Data
[p,'DPP'])-as.numeric(Temp_Data[p-1,'DPP']))))


Temp_Linear_VII[p,17]<-((as.numeric(Temp_Data[p,"Linear_Qp"])*as.numeric(Temp_Data[p
-1,'VCV'])*(exp(as.numeric(Temp_Data[p,"Growth Rate
(100)"])*(as.numeric(Temp_Data[p,'DPP'])-as.numeric(Temp_Data[p-1,'DPP'])))-exp(-Dea
th_Rate*(as.numeric(Temp_Data[p,'DPP'])-as.numeric(Temp_Data[p-1,'DPP'])))))/(Death_
Rate+as.numeric(Temp_Data[p,"Growth Rate
(100)"])))+(as.numeric(Temp_Linear_VII[p-1,17])*exp(-Death_Rate*(as.numeric(Temp_Dat
a[p,'DPP'])-as.numeric(Temp_Data[p-1,'DPP']))))


Temp_Linear_VII[p,18]<-((as.numeric(Temp_Data[p,"Linear_Qp"])*as.numeric(Temp_Data[p
-1,'VCV'])*(exp(as.numeric(Temp_Data[p,"Growth Rate
(200)"])*(as.numeric(Temp_Data[p,'DPP'])-as.numeric(Temp_Data[p-1,'DPP'])))-exp(-Dea
th_Rate*(as.numeric(Temp_Data[p,'DPP'])-as.numeric(Temp_Data[p-1,'DPP'])))))/(Death_
Rate+as.numeric(Temp_Data[p,"Growth Rate
(200)"])))+(as.numeric(Temp_Linear_VII[p-1,18])*exp(-Death_Rate*(as.numeric(Temp_Dat
a[p,'DPP'])-as.numeric(Temp_Data[p-1,'DPP']))))


Temp_Linear_VII[p,19]<-((as.numeric(Temp_Data[p,"Linear_Qp"])*as.numeric(Temp_Data[p
-1,'VCV'])*(exp(as.numeric(Temp_Data[p,"Growth Rate
(600)"])*(as.numeric(Temp_Data[p,'DPP'])-as.numeric(Temp_Data[p-1,'DPP'])))-exp(-Dea
th_Rate*(as.numeric(Temp_Data[p,'DPP'])-as.numeric(Temp_Data[p-1,'DPP'])))))/(Death_
Rate+as.numeric(Temp_Data[p,"Growth Rate
(600)"])))+(as.numeric(Temp_Linear_VII[p-1,19])*exp(-Death_Rate*(as.numeric(Temp_Dat
a[p,'DPP'])-as.numeric(Temp_Data[p-1,'DPP']))))


Temp_Linear_VII[p,20]<-((as.numeric(Temp_Data[p,"Linear_Qp"])*as.numeric(Temp_Data[p
-1,'VCV'])*(exp(as.numeric(Temp_Data[p,"Growth Rate (linearized
hr)"])*(as.numeric(Temp_Data[p,'DPP'])-as.numeric(Temp_Data[p-1,'DPP'])))-exp(-Death
_Rate*(as.numeric(Temp_Data[p,'DPP'])-as.numeric(Temp_Data[p-1,'DPP'])))))/(Death_Ra
te+as.numeric(Temp_Data[p,"Growth Rate (linearized
hr)"])))+(as.numeric(Temp_Linear_VII[p-1,20])*exp(-Death_Rate*(as.numeric(Temp_Data[
```

```
p,'DPP'])-as.numeric(Temp_Data[p-1,'DPP']))))

Temp_Linear_VII[p,21]<-((as.numeric(Temp_Data[p,"Linear_Qp"])*as.numeric(Temp_Data[p
-1,'VCV'])*(exp(as.numeric(Temp_Data[p,"Growth Rate (linearized
2hr)"])*(as.numeric(Temp_Data[p,'DPP'])-as.numeric(Temp_Data[p-1,'DPP'])))-exp(-Deat
h_Rate*(as.numeric(Temp_Data[p,'DPP'])-as.numeric(Temp_Data[p-1,'DPP'])))))/(Death_R
ate+as.numeric(Temp_Data[p,"Growth Rate (linearized
2hr)"])))+(as.numeric(Temp_Linear_VII[p-1,21])*exp(-Death_Rate*(as.numeric(Temp_Data
[p,'DPP'])-as.numeric(Temp_Data[p-1,'DPP']))))

Temp_Linear_VII[p,22]<-((as.numeric(Temp_Data[p,"Linear_Qp"])*as.numeric(Temp_Data[p
-1,'VCV'])*(exp(as.numeric(Temp_Data[p,"Growth Rate (linearized
3hr)"])*(as.numeric(Temp_Data[p,'DPP'])-as.numeric(Temp_Data[p-1,'DPP'])))-exp(-Deat
h_Rate*(as.numeric(Temp_Data[p,'DPP'])-as.numeric(Temp_Data[p-1,'DPP'])))))/(Death_R
ate+as.numeric(Temp_Data[p,"Growth Rate (linearized
3hr)"])))+(as.numeric(Temp_Linear_VII[p-1,22])*exp(-Death_Rate*(as.numeric(Temp_Data
[p,'DPP'])-as.numeric(Temp_Data[p-1,'DPP']))))


Temp_Linear_VII[p,23]<-((as.numeric(Temp_Data[p,"Shift_Linear_Qp"])*as.numeric(Temp_
Data[p-1,'VCV'])*(exp(as.numeric(Temp_Data[p,"Growth Rate
(min)"])*(as.numeric(Temp_Data[p,'DPP'])-as.numeric(Temp_Data[p-1,'DPP'])))-exp(-Dea
th_Rate*(as.numeric(Temp_Data[p,'DPP'])-as.numeric(Temp_Data[p-1,'DPP'])))))/(Death_
Rate+as.numeric(Temp_Data[p,"Growth Rate
(min)"])))+(as.numeric(Temp_Linear_VII[p-1,23])*exp(-Death_Rate*(as.numeric(Temp_Dat
a[p,'DPP'])-as.numeric(Temp_Data[p-1,'DPP']))))

Temp_Linear_VII[p,24]<-((as.numeric(Temp_Data[p,"Shift_Linear_Qp"])*as.numeric(Temp_
Data[p-1,'VCV'])*(exp(as.numeric(Temp_Data[p,"Growth Rate
(hr)"])*(as.numeric(Temp_Data[p,'DPP'])-as.numeric(Temp_Data[p-1,'DPP'])))-exp(-Deat
h_Rate*(as.numeric(Temp_Data[p,'DPP'])-as.numeric(Temp_Data[p-1,'DPP'])))))/(Death_R
ate+as.numeric(Temp_Data[p,"Growth Rate
(hr)"])))+(as.numeric(Temp_Linear_VII[p-1,24])*exp(-Death_Rate*(as.numeric(Temp_Data
[p,'DPP'])-as.numeric(Temp_Data[p-1,'DPP']))))

Temp_Linear_VII[p,25]<-((as.numeric(Temp_Data[p,"Shift_Linear_Qp"])*as.numeric(Temp_
Data[p-1,'VCV'])*(exp(as.numeric(Temp_Data[p,"Growth Rate (min avg
hr)"])*(as.numeric(Temp_Data[p,'DPP'])-as.numeric(Temp_Data[p-1,'DPP'])))-exp(-Death
_Rate*(as.numeric(Temp_Data[p,'DPP'])-as.numeric(Temp_Data[p-1,'DPP'])))))/(Death_Ra
te+as.numeric(Temp_Data[p,"Growth Rate (min avg
hr)"])))+(as.numeric(Temp_Linear_VII[p-1,25])*exp(-Death_Rate*(as.numeric(Temp_Data[
p,'DPP'])-as.numeric(Temp_Data[p-1,'DPP']))))
```

```
Temp_Linear_VII[p,26]<-((as.numeric(Temp_Data[p,"Shift_Linear_Qp"])*as.numeric(Temp_
Data[p-1,'VCV'])*(exp(as.numeric(Temp_Data[p,"Growth Rate
(50)"])*(as.numeric(Temp_Data[p,'DPP'])-as.numeric(Temp_Data[p-1,'DPP'])))-exp(-Deat
h_Rate*(as.numeric(Temp_Data[p,'DPP'])-as.numeric(Temp_Data[p-1,'DPP']))))/(Death_R
ate+as.numeric(Temp_Data[p,"Growth Rate
(50)"])))+(as.numeric(Temp_Linear_VII[p-1,26])*exp(-Death_Rate*(as.numeric(Temp_Data
[p,'DPP'])-as.numeric(Temp_Data[p-1,'DPP']))))


Temp_Linear_VII[p,27]<-((as.numeric(Temp_Data[p,"Shift_Linear_Qp"])*as.numeric(Temp_
Data[p-1,'VCV'])*(exp(as.numeric(Temp_Data[p,"Growth Rate (min avg
2hr)"])*(as.numeric(Temp_Data[p,'DPP'])-as.numeric(Temp_Data[p-1,'DPP'])))-exp(-Deat
h_Rate*(as.numeric(Temp_Data[p,'DPP'])-as.numeric(Temp_Data[p-1,'DPP']))))/(Death_R
ate+as.numeric(Temp_Data[p,"Growth Rate (min avg
2hr)"])))+(as.numeric(Temp_Linear_VII[p-1,27])*exp(-Death_Rate*(as.numeric(Temp_Data
[p,'DPP'])-as.numeric(Temp_Data[p-1,'DPP']))))


Temp_Linear_VII[p,28]<-((as.numeric(Temp_Data[p,"Shift_Linear_Qp"])*as.numeric(Temp_
Data[p-1,'VCV'])*(exp(as.numeric(Temp_Data[p,"Growth Rate
(100)"])*(as.numeric(Temp_Data[p,'DPP'])-as.numeric(Temp_Data[p-1,'DPP'])))-exp(-Dea
th_Rate*(as.numeric(Temp_Data[p,'DPP'])-as.numeric(Temp_Data[p-1,'DPP']))))/(Death_
Rate+as.numeric(Temp_Data[p,"Growth Rate
(100)"])))+(as.numeric(Temp_Linear_VII[p-1,28])*exp(-Death_Rate*(as.numeric(Temp_Dat
a[p,'DPP'])-as.numeric(Temp_Data[p-1,'DPP']))))


Temp_Linear_VII[p,29]<-((as.numeric(Temp_Data[p,"Shift_Linear_Qp"])*as.numeric(Temp_
Data[p-1,'VCV'])*(exp(as.numeric(Temp_Data[p,"Growth Rate
(200)"])*(as.numeric(Temp_Data[p,'DPP'])-as.numeric(Temp_Data[p-1,'DPP'])))-exp(-Dea
th_Rate*(as.numeric(Temp_Data[p,'DPP'])-as.numeric(Temp_Data[p-1,'DPP']))))/(Death_
Rate+as.numeric(Temp_Data[p,"Growth Rate
(200)"])))+(as.numeric(Temp_Linear_VII[p-1,29])*exp(-Death_Rate*(as.numeric(Temp_Dat
a[p,'DPP'])-as.numeric(Temp_Data[p-1,'DPP']))))


Temp_Linear_VII[p,30]<-((as.numeric(Temp_Data[p,"Shift_Linear_Qp"])*as.numeric(Temp_
Data[p-1,'VCV'])*(exp(as.numeric(Temp_Data[p,"Growth Rate
(600)"])*(as.numeric(Temp_Data[p,'DPP'])-as.numeric(Temp_Data[p-1,'DPP'])))-exp(-Dea
th_Rate*(as.numeric(Temp_Data[p,'DPP'])-as.numeric(Temp_Data[p-1,'DPP']))))/(Death_
Rate+as.numeric(Temp_Data[p,"Growth Rate
(600)"])))+(as.numeric(Temp_Linear_VII[p-1,30])*exp(-Death_Rate*(as.numeric(Temp_Dat
a[p,'DPP'])-as.numeric(Temp_Data[p-1,'DPP']))))


Temp_Linear_VII[p,31]<-((as.numeric(Temp_Data[p,"Shift_Linear_Qp"])*as.numeric(Temp_
Data[p-1,'VCV'])*(exp(as.numeric(Temp_Data[p,"Growth Rate (linearized
hr)"])*(as.numeric(Temp_Data[p,'DPP'])-as.numeric(Temp_Data[p-1,'DPP'])))-exp(-Death
_Rate*(as.numeric(Temp_Data[p,'DPP'])-as.numeric(Temp_Data[p-1,'DPP']))))/(Death_Ra
te+as.numeric(Temp_Data[p,"Growth Rate (linearized
```

```
hr)"])))+(as.numeric(Temp_Linear_VII[p-1,31])*exp(-Death_Rate*(as.numeric(Temp_Data[
p,'DPP'])-as.numeric(Temp_Data[p-1,'DPP']))))


Temp_Linear_VII[p,32]<-((as.numeric(Temp_Data[p,"Shift_Linear_Qp"])*as.numeric(Temp_
Data[p-1,'VCV'])*(exp(as.numeric(Temp_Data[p,"Growth Rate (linearized
2hr)"])*(as.numeric(Temp_Data[p,'DPP'])-as.numeric(Temp_Data[p-1,'DPP'])))-exp(-Deat
h_Rate*(as.numeric(Temp_Data[p,'DPP'])-as.numeric(Temp_Data[p-1,'DPP'])))))/(Death_R
ate+as.numeric(Temp_Data[p,"Growth Rate (linearized
2hr)"])))+(as.numeric(Temp_Linear_VII[p-1,32])*exp(-Death_Rate*(as.numeric(Temp_Data
[p,'DPP'])-as.numeric(Temp_Data[p-1,'DPP']))))


Temp_Linear_VII[p,33]<-((as.numeric(Temp_Data[p,"Shift_Linear_Qp"])*as.numeric(Temp_
Data[p-1,'VCV'])*(exp(as.numeric(Temp_Data[p,"Growth Rate (linearized
3hr)"])*(as.numeric(Temp_Data[p,'DPP'])-as.numeric(Temp_Data[p-1,'DPP'])))-exp(-Deat
h_Rate*(as.numeric(Temp_Data[p,'DPP'])-as.numeric(Temp_Data[p-1,'DPP'])))))/(Death_R
ate+as.numeric(Temp_Data[p,"Growth Rate (linearized
3hr)"])))+(as.numeric(Temp_Linear_VII[p-1,33])*exp(-Death_Rate*(as.numeric(Temp_Data
[p,'DPP'])-as.numeric(Temp_Data[p-1,'DPP']))))

      }



    }

    if (r==1)

    {

      Linear_VII<-data.frame(matrix(nrow=nrow(Temp_Linear_VII),ncol=33))

      Linear_VII[r:nrow(Temp_Linear_VII),]<-Temp_Linear_VII

      q<-nrow(Linear_VII)

    } else

    {

      Linear_VII[(q+1):(q+nrow(Temp_Linear_VII)),]<-Temp_Linear_VII

      q<-nrow(Linear_VII)

    }

  }
```

```
Interpolated_Data[,c((ncol(Temp_Interpolated_Data)+1):ncol(Interpolated_Data))]<-Lin
ear_VII


Final_Data<-OUR_Data

Final_Data<-merge(Final_Data,Interpolated_Data,all=TRUE)


l<-1

pdf("VII_Trends.pdf",onefile=TRUE)

for (l in 1:Num_Batch_ID)

{

  h<-filter(Final_Data,`Batch
ID`==as.character(Batch_IDs[l,])&`Infection?`=="INFECTION")%>%mutate(VII_SD=VII*0.3)

  a<-ggplot(h,aes(x=DPI))+

    geom_point(aes(y=Step_100_row_VII,color="Step_100_row_VII"),shape=16,size=1)+

    geom_point(aes(y=VII,color="VII"),shape=17,size=2)+

    geom_errorbar(aes(ymin=VII-VII_SD,ymax=VII+VII_SD),width=0.05)+


scale_color_manual("",values=c("Step_100_row_VII"="pink","VII"="blue"),breaks=c("Ste
p_100_row_VII","VII"),labels=c("Interpolated VII","Real VII"))+

    theme_classic()+

    labs(

      title = "Interpolated and Real VII Data",

      subtitle = as.character(Batch_IDs[l,]),

      x = "DPI",

      y = "VII Titer",

    )

  print(a)
```

```
}

dev.off()


Final_File_Name<-'Interpolated_OUR_Data_1DPI_TRB_07Jun2023.xlsx'

write_xlsx(Final_Data,Final_File_Name)
```
